# Efficient Chemoenzymatic Synthesis of N‐Glycans with a β1,4‐Galactosylated Bisecting GlcNAc Motif

**DOI:** 10.1002/cbic.202000268

**Published:** 2020-08-19

**Authors:** Michael Weiss, Dimitri Ott, Theodoros Karagiannis, Markus Weishaupt, Mathäus Niemietz, Steffen Eller, Marie Lott, Mónica Martínez‐Orts, Ángeles Canales, Nahid Razi, James C. Paulson, Carlo Unverzagt

**Affiliations:** ^1^ University of Bayreuth Bioorganic Chemistry Universitätsstraße 30 95447 Bayreuth Germany; ^2^ Dpto. Química Orgánica I Fac. Ciencias Químicas Universidad Complutense de Madrid Avd. Complutense s/n 28040 Madrid Spain; ^3^ Depts. of Molecular Medicine and Immunology and Microbiology The Scripps Research Institute 10550 N. Torrey Pines Road, La Jolla CA 92037 USA

**Keywords:** N-glycans, glycosylation, glycobiology, immunoglobulin, paramagnetic NMR spectroscopy

## Abstract

In human serum immunoglobulin G (IgG), a rare modification of biantennary complex N‐glycans lead to a β1,4‐galactosylated bisecting GlcNAc branch. We found that the bisecting GlcNAc on a biantennary core‐fucosylated N‐glycan was enzymatically galactosylated under stringent reaction conditions. Further optimizations led to an efficient enzymatic approach to this particular modification for biantennary substrates. Notably, tri‐ and tetra‐antennary complex N‐glycans were not converted by bovine galactosyltransferase. An N‐glycan with a galactosylated bisecting GlcNAc was linked to a lanthanide binding tag. The pseudo‐contact shifts (PCS) obtained from the corresponding Dy‐complex were used to calculate the conformational preferences of the rare N‐glycan. Besides two extended conformations only a single folded conformation was found.

## Graphical abstract

**From analytical oddity to conformational study**. A rare N‐glycan with a galactosylated bisecting GlcNAc present in human serum immunoglobulin (IgG1) was obtained by chemoenzymatic synthesis from **A** and derivatized with a lanthanide tag (**B**). Paramagnetic NMR studies revealed a conformational behavior deviating from that of the non‐bisected biantennary N‐glycan.

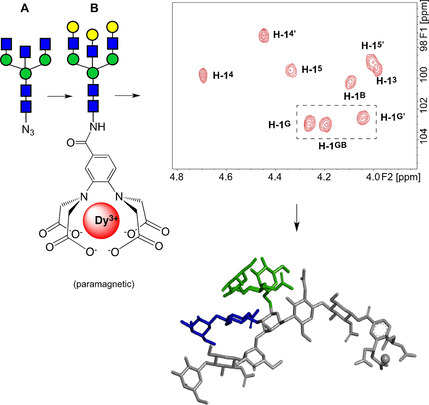

## Introduction

**Scheme 1:**
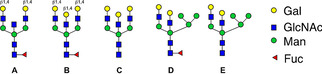
Biantennary N‐glycan with unmodified bisecting GlcNAc (**A**); complex N‐glycans found on human serum IgG bearing a β1,4‐galactosylated bisecting GlcNAc motif (**B**, **C**); hybrid N‐glycans from 19 A glycoprotein with a galactosylated bisecting GlcNAc motif (**D**, **E**).

Immunoglobulins are one of the best‐studied classes of glycoproteins due to their high therapeutic value. The N‐glycans of immunoglobulins are known to affect the biological activity of antibodies, for example, by modulating the interactions of the Fc part.^[1]^ Thus, the glycosylation pattern of therapeutic antibodies is thoroughly characterized. Originating from an unexpected side reaction during the enzymatic galactosylation of a synthetic bisected N‐glycan we became aware that galactosylated bisecting GlcNAc moieties are present in human serum IgG. The Gal‐β1,4‐bisecting GlcNAc motif was initially discovered by Nishimura^[2]^ and later confirmed by Rudd^[3]^ (Scheme 1). A similar motif was also found on bisected hybrid N‐glycans of the 19 A glycoprotein derived from a lectin‐resistant HEK cell line.^[3]^ In cells lacking GlcNAc‐transferase II the truncated bisected N‐glycans leads to a Gal‐β1,4‐bisecting GlcNAc motif, which can be further modified.^[4]^ An enzymatic galactosylation of a single GlcNAc β1,2‐linked to the central β‐mannose was found for a synthetic N‐glycan bearing the LEC 14 antigen.^[5]^

## Results and Discussion

As part of a collaboration with the Consortium for Functional Glycomics (CFG), we synthesized various bisected complex‐type N‐glycans and functionalized them with a bifunctional spacer for glycan microarray printing.^[6]^ The bisected N‐glycans^[7]^ were synthesized chemically following a modular approach and deprotected to the free N‐glycans.

**Scheme 2:**
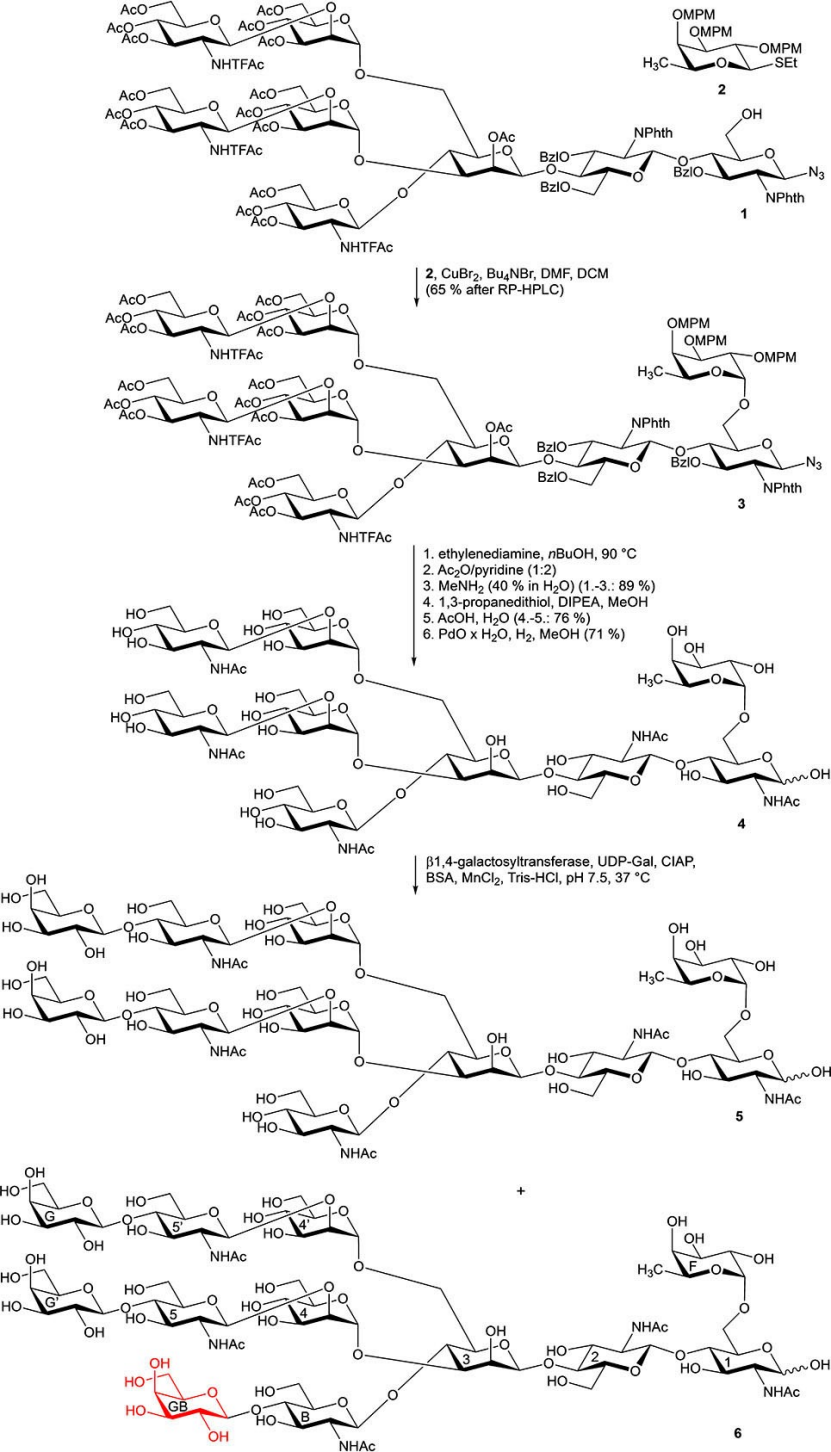
Synthesis of N‐glycan **3**, deprotection to hemiacetal **4** and enzymatic galactosylation to **5** and **6**. The numbering of the residues for NMR assignments is shown for **6**.

In the case of the core fucosylated bisected compound **4**, precursor **1** was obtained from three building blocks^[8]^ and was subsequently fucosylated using thiofucoside **2** ^[9]^ (Scheme 2). The low stereoselectivity (α/β ratio 5.4 : 1) required the separation of the unnatural β‐anomer **3** **b** by preparative HPLC. Subsequently, the protecting groups of nonasaccharide **3** were removed in a multistep procedure. After treatment with ethylenediamine in *n*‐butanol an acetylation/deacetylation sequence yielded a benzylated nonasaccharide azide intermediate, which was reduced to the anomeric amine using propanedithiol.^[10]^ Complete removal of residual thiol was crucial prior to acidic hydrolysis of the glycosylamine. The final catalytic hydrogenation gave the free N‐glycan **4** in good overall yield. The enzymatic galactosylation of **4** was carried out using UDP‐Gal, bovine galactosyltransferase and calf alkaline phosphatase (CIAP).^[11]^ Analysis of the reaction mixture after three days by MALDI‐TOF‐MS showed the desired galactosylated N‐glycan **5** but also a side product (ca. 23 %) with the mass of an additional galactose unit and we assumed that the additional galactose might be attached to the bisecting GlcNAc (**6**). We noticed that relative to previous galactosylations of biantennary bisected N‐glycan‐conjugates^[12]^ the concentration of the N‐glycan acceptor (**4**) was higher (4x) as well as the relative amount of galactosyltransferase (2x). Using even higher concentrations of N‐glycan acceptor **4** and repeated addition of galactosyltransferase gave nearly complete conversion to the trigalactosylated product **6**. On a preparative scale **4** (3.7 mg; 19 mM) was incubated with 800 mU of galactosyltransferase added in three portions over 9 days, which led to complete conversion according to MALDI‐TOF‐MS. The trigalactosylated compound **6** was isolated by gel filtration in a yield of 74 %. Using a lower concentration of **4** in combination with lower amounts of galactosyltransferase furnished the selectively digalactosylated compound **5** in 78 % yield.

Both compounds **5** and **6** were characterized by a set of 2D NMR experiments revealing that the third galactose was indeed transferred to O‐4 of the bisecting GlcNAc moiety. This was evident from the ^1^H signal of H‐4^B^ shifting from 3.06 to 3.3 ppm and the ^13^C signal of C‐4^B^ moving from 72.7 to 81.7 ppm indicative for a glycosylation at C4^B^. The glycans **5** and **6** were equipped with a bifunctional linker and added to the repertoire of the CFG glycan array (data not shown).

**Scheme 3:**
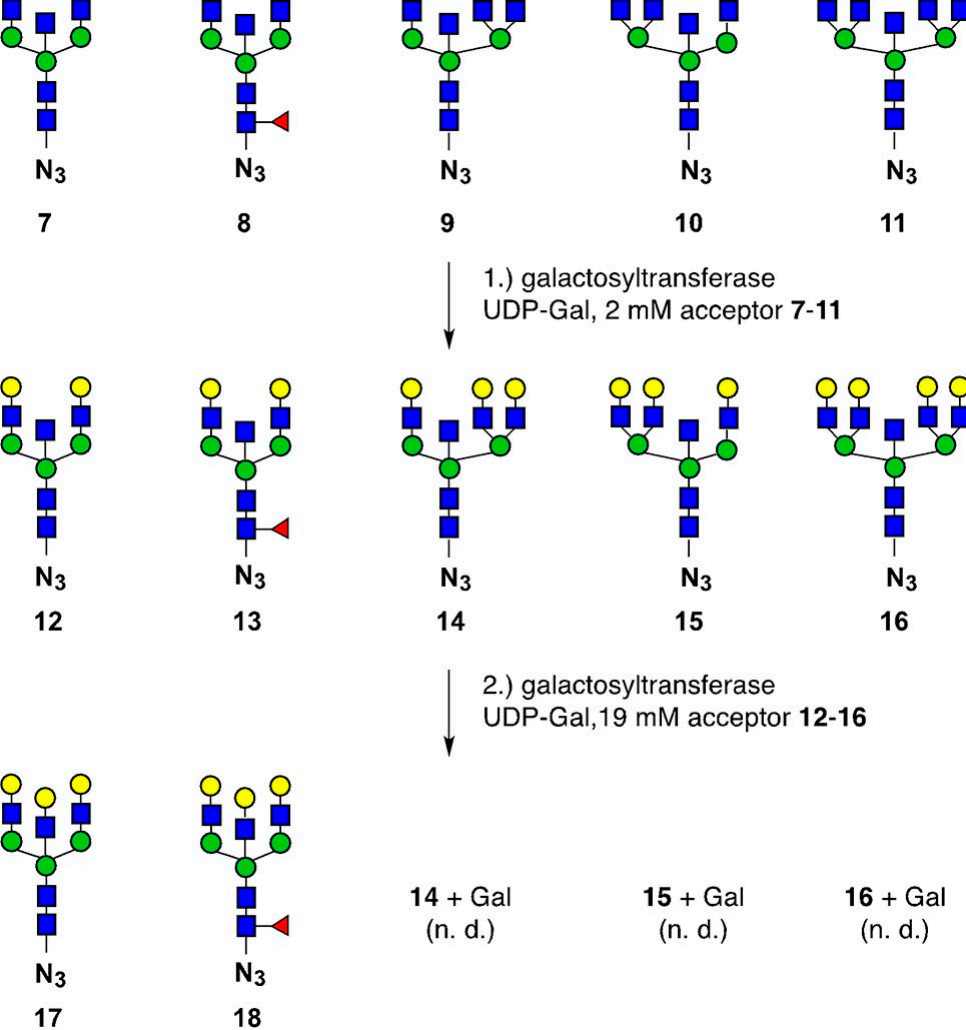
The panel of bisected N‐glycan azides (**7**–**11**) was enzymatically galactosylated under dilute (1.) and subsequently under stringent (2.) conditions. Transfer of an additional galactose to the bisecting GlcNAc was observed only in the case of the biantennary N‐glycans **17** and **18**. (n. d.=not detected).

Next the scope of the enzymatic elongation of a bisecting GlcNAc was investigated on an analytical scale using a series of fully synthetic bisected N‐glycan azides (Scheme 3). The panel of potential substrates consisted of the unsubstituted bisected N‐glycan **7**,^[7]^ the corresponding core‐fucosylated glycan **8** ^[8]^ and bisected compounds with three or four antennae (**9**–**11**).^[7]^ When attempting to apply the stringent conditions of galactosylation directly to the acceptors (19 mM **7**–**11**), strong substrate inhibition was found, which only led to sluggish and incomplete galactosylation after several days (HPLC data not shown). In contrast, dilute conditions (2 mM acceptor **7**–**11**) gave complete galactosylation of the GlcNAc termini within 1 day (**12**–**16**). The galactosylated compounds were submitted to stringent galactosylation conditions (19 mM acceptor **12**–**16**). Out of the five compounds only the biantennary N‐glycans **12** and **13** gave the corresponding product with a galactosylated bisecting GlcNAc (**17**–**18**) whereas no conversion according to LC–MS was found for the compounds with three or four antennae (**9**–**11**). The galactosylation of a bisecting GlcNAc appears to be particularly sensitive to additional steric hindrance and might not be possible in the case of complex N‐glycans with more than two antennae. According to the HPLC peak areas of the galactosylations after 3 days, the transfer of the third galactose occurs significantly more slowly (∼1/5) in the presence of a core fucose (**13**) compared with the unsubstituted bisected compound (**12**) (see S4 in the Supporting Information).

**Scheme 4:**
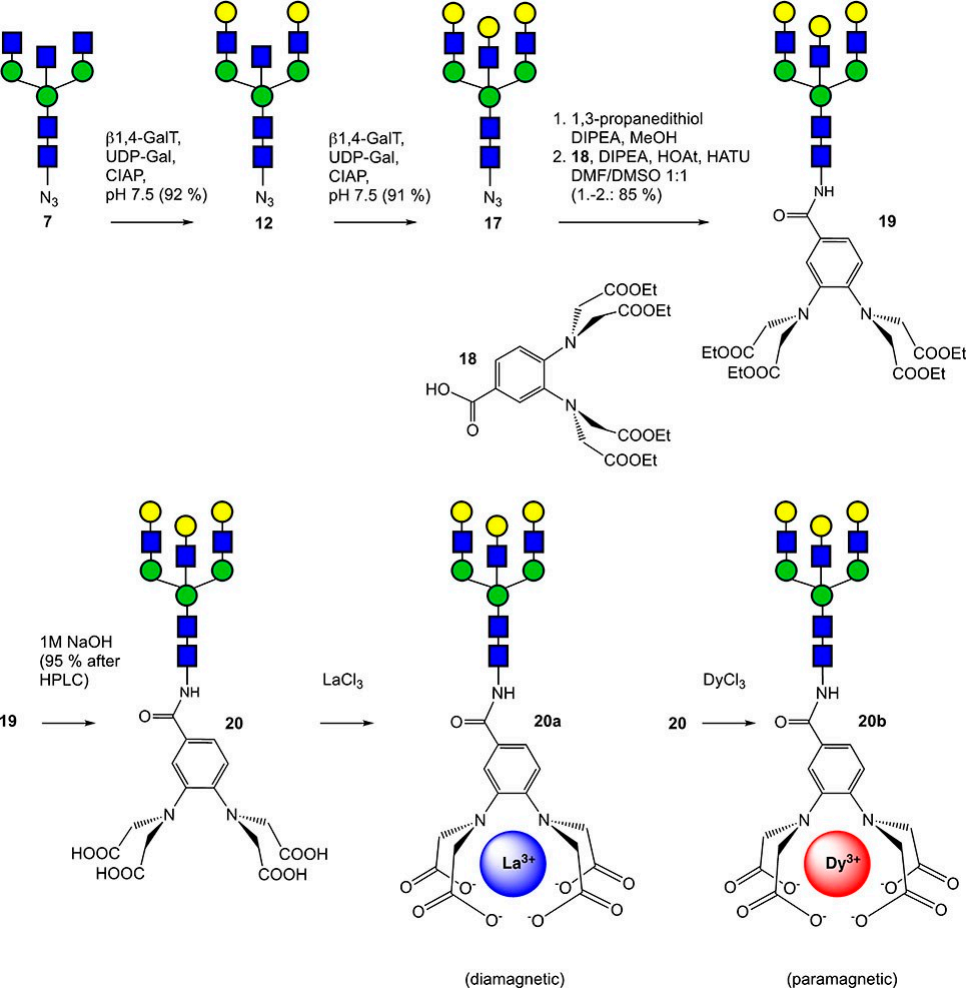
Synthesis of the trigalactosylated bisected N‐glycan azide **17**, conjugation with lanthanide binding tag **18**, deprotection and formation of the diamagnetic complex **20** **a** and the paramagnetic complex **20** **b**.

Because only the biantennary digalactosylated N‐glycan azides were accepted by galactosyltransferase, we assumed that the galactosylated bisecting GlcNAc residue might affect the conformational freedom of the neighboring antennae. We decided to investigate the conformational preferences of the N‐glycan **17** by attaching a suitable lanthanide binding tag (**19**) and subsequently measure the pseudo contact shifts of paramagnetic N‐glycan complexes to calculate the preferred conformations.^[13]^ On a preparative scale the bisected octasaccharide azide **7** was first converted to the digalactosylated compound **12** (92 % yield) and subsequently to the trigalactosylated undecasaccharide **17** (91 % after RP‐HPLC). The azide of **17** was reduced and coupled with the lanthanide binding tag **18**. Purification of the conjugate **19** by RP‐HPLC was important at this stage since a small percentage (∼12 %) of α‐configured product also formed. The ethyl esters of **19** were saponified in 1 M NaOH followed by a purification of **20** by RP‐HPLC and complexation with either LaCl_3_ or DyCl_3_ in a D_2_O‐imidazole buffer (Scheme 4.^[13b]^

**Scheme 5:**
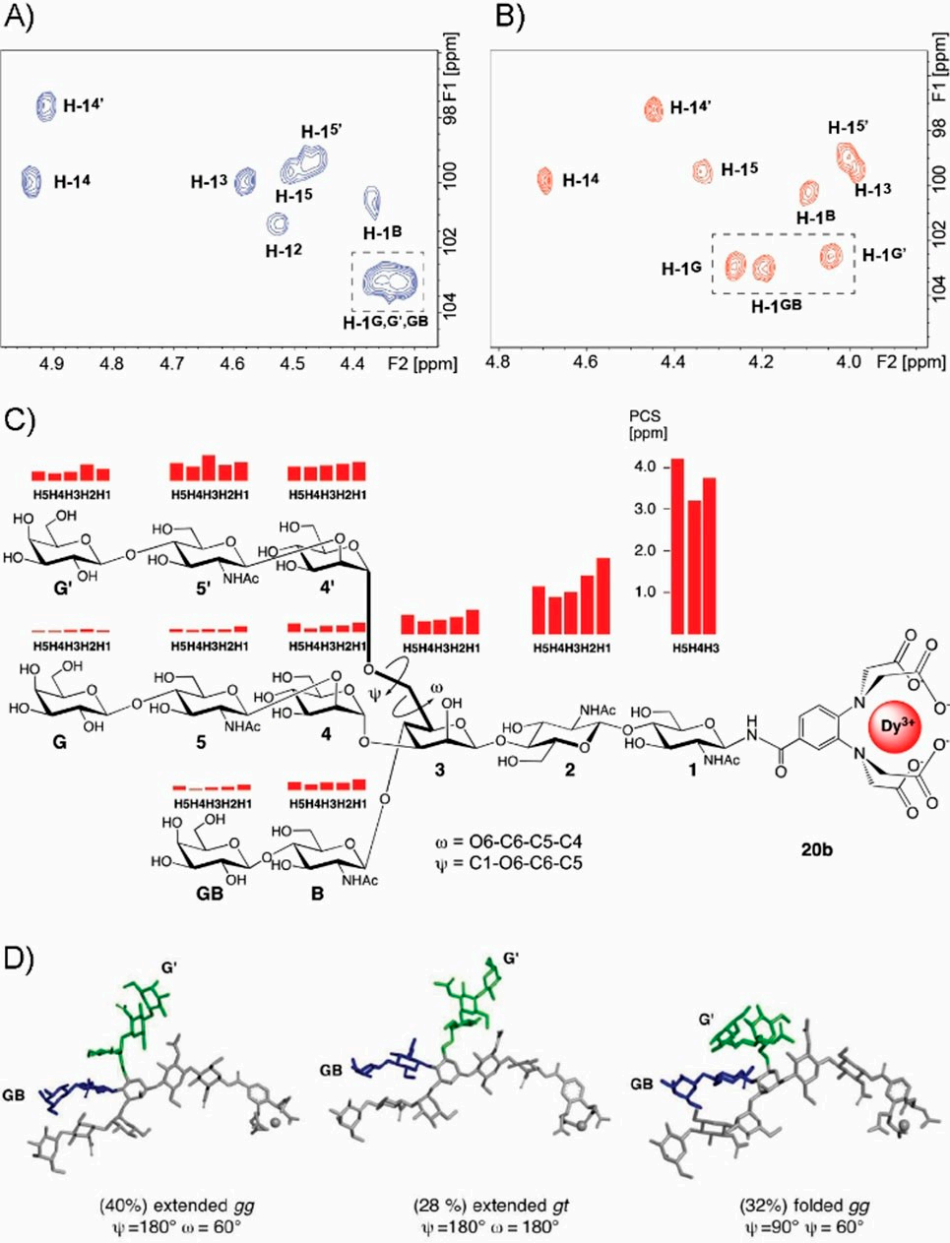
A), B) HSQC sections of the anomeric region of the diamagnetic (**20** **a**) and paramagnetic complex (**20** **b**); the three galactose signals are within the dotted lines. C) PCS of paramagnetic complex **20** **b**. D) Relative percentages of the main conformations calculated from PCS.

The conformational behavior of the trigalactosylated bisected N‐glycan **17** was characterized by using paramagnetic NMR.^[14]^ We have previously implemented this approach to determine the conformation of a non‐bisected biantennary N‐glycan and the same methodology has been employed in this work.^[13a]^ The good HSQC‐signal dispersion obtained for the paramagnetic dysprosium complex **20** **b** allowed full resolution of the anomeric signals of GlcNAc residues 5 and 5’ and the three galactoses (G, G’ and GB), which overlapped in the diamagnetic complex **20** **a** (Scheme 5A, B). In total 58 PCSs were obtained and used for conformation analysis (Scheme 5C). The PCS depends on the distance and the orientation of the NMR nuclei relative to the paramagnetic metal ion and thus provides structural information. The experimental PCs were correlated with the back‐calculated values from the different minimum energy conformations of the N‐glycan by using Mspin software.^[15]^ Iterative calculations were carried out with Mspin to obtain the number of conformers and the population of each conformer that better fit the experimental PCS values. The best correlation between experimental and calculated data was obtained considering three interconverting main conformations for the Man^4’^α1,6‐Man^3^ linkage, extended *gauche–gauche* (*gg*), extended *gauche–trans* (*gt*) and folded *gg* conformation, with populations of 40, 28, and 32 %, respectively (Scheme 5D).

The main conformations are the extended *gg* and *gt* conformers as also found in the corresponding non‐bisected biantennary N‐glycan.^[13a]^ However, a higher population (32 %) of a single folded conformation (*ψ*=90°) was obtained for the galactosylated bisected glycan compared with the non‐bisected biantennary structure, where two folded conformations were found (10 % *ψ*=60° and 10 % *ψ*=90°). Notably, the galactosylated bisected branch affects the preferred conformations of the N‐glycan leading to a threefold increase of a single folded geometry of the α1,6 arm. The extended *gt* conformation served as a starting point for a preliminary model where the bisecting GlcNAc of **12** could be placed in the acceptor site of bovine galactosyltransferase (PDB ID: 1TW5)^[16]^ with only minor adjustments of the 1,3 arm (see Schemes S7 and S8 in the Supporting Information). We are currently investigating the conformational preferences of complex N‐glycans with the typical bisecting GlcNAc‐modification lacking the additional galactose unit.

In summary a rare N‐glycan identified in human serum IgG could be synthesized by using stringent conditions for the enzymatic galactosylation of the bisecting GlcNAc moiety. This particular conversion appears to be limited to biantennary‐N‐glycans. The conformational analysis of a conjugate of the glycan bearing the galactosylated bisecting GlcNAc motif using paramagnetic NMR revealed two extended conformers but only a single folded geometry.

## Conflict of interest

The authors declare no conflict of interest.

## Supporting information

As a service to our authors and readers, this journal provides supporting information supplied by the authors. Such materials are peer reviewed and may be re‐organized for online delivery, but are not copy‐edited or typeset. Technical support issues arising from supporting information (other than missing files) should be addressed to the authors.

Supplementary

## References

[1a] T. Shinkawa , K. Nakamura , N. Yamane , E. Shoji-Hosaka , Y. Kanda , M. Sakurada , K. Uchida , H. Anazawa , M. Satoh , M. Yamasaki , N. Hanai , K. Shitara , J. Biol. Chem. 2003, 278, 3466–3473; 12427744 10.1074/jbc.M210665200

[1b] P. Sondermann , A. Pincetic , J. Maamary , K. Lammens , J. V. Ravetch , Proc. Natl. Acad. Sci. USA 2013, 110, 9868–9872; 23697368 10.1073/pnas.1307864110 PMC3683708

[1c] T. Li , D. J. Di Lillo , S. Bournazos , J. P. Giddens , J. V. Ravetch , L. X. Wang , Proc. Natl. Acad. Sci. USA 2017, 114, 3485–3490; 28289219 10.1073/pnas.1702173114 PMC5380036

[1d] R. M. Anthony , F. Nimmerjahn , D. J. Ashline , V. N. Reinhold , J. C. Paulson , J. V. Ravetch , Science 2008, 320, 373–376. 18420934 10.1126/science.1154315 PMC2409116

[2] Y. Takegawa , K. Deguchi , H. Nakagawa , S. Nishimura , Anal. Chem. 2005, 77, 6062–6068. 16159142 10.1021/ac050843e

[3] D. J. Harvey , M. Crispin , C. Scanlan , B. B. Singer , L. Lucka , V. T. Chang , C. M. Radcliffe , S. Thobhani , C. T. Yuen , P. M. Rudd , Rapid Commun. Mass Spectrom. 2008, 22, 1047–1052. 18327885 10.1002/rcm.3470

[4] Y. Wang , J. Tan , M. Sutton-Smith , D. Ditto , M. Panico , R. M. Campbell , N. M. Varki , J. M. Long , J. Jaeken , S. R. Levinson , A. Wynshaw-Boris , H. R. Morris , D. Le , A. Dell , H. Schachter , J. D. Marth , Glycobiology 2001, 11, 1051–1070. 11805078 10.1093/glycob/11.12.1051

[5] I. Prahl , C. Unverzagt , Angew. Chem. Int. Ed. 2002, 41, 4259–4262; 10.1002/1521-3773(20021115)41:22<4259::AID-ANIE4259>3.0.CO;2-A 12434356

[6] O. Blixt , S. Head , T. Mondala , C. Scanlan , M. E. Huflejt , R. Alvarez , M. C. Bryan , F. Fazio , D. Calarese , J. Stevens , N. Razi , D. J. Stevens , J. J. Skehel , I. van Die , D. R. Burton , I. A. Wilson , R. Cummings , N. Bovin , C. H. Wong , J. C. Paulson , Proc. Natl. Acad. Sci. USA 2004, 101, 17033–17038. 15563589 10.1073/pnas.0407902101 PMC534418

[7] M. Mönnich , S. Eller , T. Karagiannis , L. Perkams , T. Luber , D. Ott , M. Niemietz , J. Hoffman , J. Walcher , L. Berger , M. Pischl , M. Weishaupt , C. Wirkner , R. G. Lichtenstein , C. Unverzagt , Angew. Chem. Int. Ed. 2016, 55, 10487–10492; 10.1002/anie.201604190 27443163

[8] T. Luber , M. Niemietz , T. Karagiannis , M. Mönnich , D. Ott , L. Perkams , J. Walcher , L. Berger , M. Pischl , M. Weishaupt , S. Eller , J. Hoffman , C. Unverzagt , Angew. Chem. Int. Ed. 2018, 57, 14543–14549. 10.1002/anie.201807742 30144245

[9] D. Ott , J. Seifert , I. Prahl , M. Niemietz , J. Hoffman , J. Guder , M. Mönnich , C. Unverzagt , Eur. J. Org. Chem. 2012, 5054–5068.

[10] C. Unverzagt , Angew. Chem. Int. Ed. Engl. 1996, 35, 2350–2353;

[11] C. Unverzagt , H. Kunz , J. C. Paulson , J. Am. Chem. Soc. 1990, 112, 9308–9309.

[12a] S. André , C. Unverzagt , S. Kojima , M. Frank , J. Seifert , C. Fink , K. Kayser , C. W. von der Lieth , H. J. Gabius , Eur. J. Biochem. 2004, 271, 118–134; 14686925 10.1046/j.1432-1033.2003.03910.x

[12b] S. André , T. Kozar , R. Schuberth , C. Unverzagt , S. Kojima , H. J. Gabius , Biochemistry 2007, 46, 6984–6995. 17497937 10.1021/bi7000467

[13a] A. Canales , A. Mallagaray , J. Pérez-Castells , I. Boos , C. Unverzagt , S. André , H. J. Gabius , F. J. Cañada , J. Jiménez-Barbero , Angew. Chem. Int. Ed. 2013, 52, 13789–13793; 10.1002/anie.201307845 24346952

[13b] A. Canales , I. Boos , L. Perkams , L. Karst , T. Luber , T. Karagiannis , G. Domínguez , F. J. Cañada , J. Pérez-Castells , D. Häussinger , C. Unverzagt , J. Jiménez-Barbero , Angew. Chem. Int. Ed. 2017, 56, 14987–14991; 10.1002/anie.201709130 PMC5813150 28991403

[14a] S. Yamamoto , T. Yamaguchi , M. Erdelyi , C. Griesinger , K. Kato , Chem. Eur. J. 2011, 17, 9280–9282; 21774005 10.1002/chem.201100856

[14b] T. Yamaguchi , Y. Sakae , Y. Zhang , S. Yamamoto , Y. Okamoto , K. Kato , Angew. Chem. Int. Ed. 2014, 53, 10941–10944; 10.1002/anie.201406145 25196214

[15a] http://www.mestrelab.com Program Mspin;

[15b] A. Navarro-Vazquez , Magn. Reson. Chem. 2012, 50, 73–9. 10.1002/mrc.3905 23280663

[16] B. Ramakrishnan , E. Boeggeman , P. K. Qasba , Biochemistry 2004, 43, 12513–12522. 15449940 10.1021/bi049007+

