# Supplementary material for: Efficient Chemoenzymatic Synthesis of N‐Glycans with a β1,4‐Galactosylated Bisecting GlcNAc Motif
Source: Chembiochem. 2020 Aug 19;21(22):3212–5. doi: 10.1002/cbic.202000268 (PMC7723014; doi:10.1002/cbic.202000268)
Supplement: Supplementary file 1 — Supplementary [file CBIC-21-3212-s001.pdf]

ChemBioChem

Supporting Information

Efficient Chemoenzymatic Synthesis of N-Glycans with a β1,4-Galactosylated Bisecting GlcNAc Motif

Michael Weiss, Dimitri Ott, Theodoros Karagiannis, Markus Weishaupt, Mathäus Niemietz, Steffen Eller, Marie Lott, Mónica Martínez-Orts, Ángeles Canales, Nahid Razi, James C. Paulson, and Carlo Unverzagt*

Supporting Information for: cbic.202000268

General methods

Solvents were dried according to standard methods. Molecular sieves were activated prior to use by heating under high vacuum. Optical rotations were measured with a Perkin–Elmer 241 polarimeter at 589 nm. NMR spectra were recorded with Bruker Avance 360, AMX 500 and DMX 500 instruments. Coupling constants are reported in Hz. The compounds were characterized by the ^{1}H and ^{13}C resonances from a set of 1D and 2D NMR experiments.[1] The resonances were assigned according to Scheme S1. ESI-TOF mass spectra were recorded with a Micromass LCT instrument coupled to an Agilent 1100 HPLC or a Waters ACQUITY UPLC H Class System with a photodiode array detector using solvent A (H_2O + 0.1 % HCOOH) and B (CH_3CN + 0.1 % HCOOH). HR-ESI mass spectra were recorded with a Thermo Q Exactive Orbitrap coupled to a Dionex UHPLC. Flash chromatography was performed manually on silica gel 60, (230–400 mesh, Merck Darmstadt) or on a GRACE Reveleris® iES-Flashchromatography system with an ELS-detector. Size-exclusion chromatography was performed on a Pharmacia Äkta Purifier 100. The reactions were monitored by thin layer chromatography on coated aluminum plates (silica gel 60 GF_{254}, Merck Darmstadt). Spots were detected by UV light or by charring with a 1:1 mixture of 2 n H_2SO_4/0.2% resorcinol monomethyl ether in ethanol.

Bovine GlcNAc β-1,4-galactosyltransferase was obtained from Sigma. Calf intestinal alkaline phosphatase was from Roche.

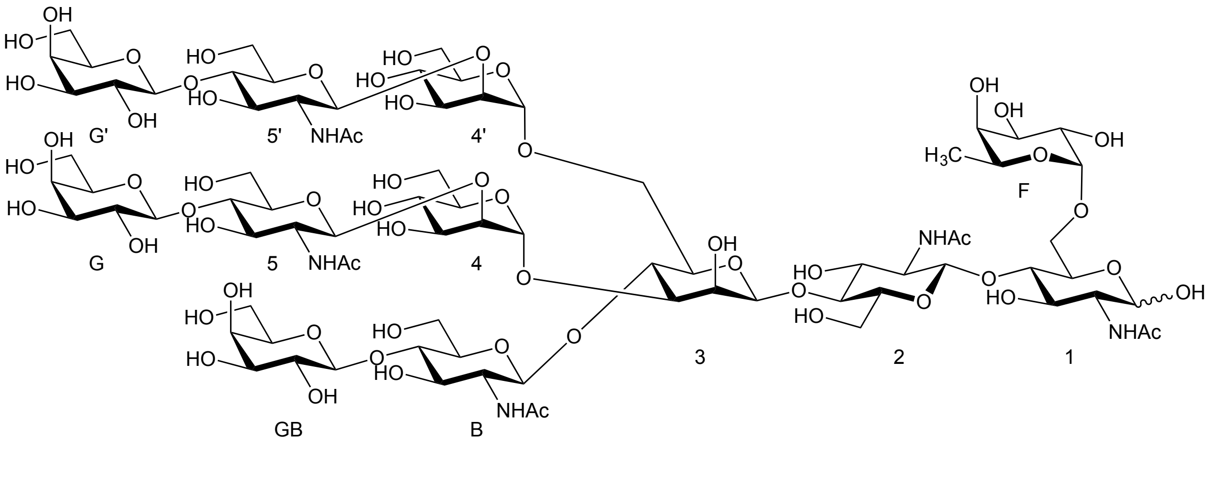

Scheme S1: *Assignment of NMR-signals.*

Fucosylation of 1 and deprotection of 3

O-(3,4,6-Tri-O-acetyl-2-deoxy-2-trifluoracetamido-β-D-glucopyranosyl)-(1→2)-O-(3,4,6-tri-O-acetyl-α-D-mannopyranosyl)-(1→3)-[O-(3,4,6-tri-O-acetyl-2-deoxy-2-trifluoracetamido-β-D-glucopyranosyl)-(1→4)]-[O-(3,4,6-tri-O-acetyl-2-deoxy-2-trifluoracetamido-β-D-glucopyranosyl)-(1→2)-O-(3,4,6-tri-O-acetyl-α-D-mannopyranosyl)-(1→6)]-O-(2-O-acetyl-β-D-mannopyranosyl)-(1→4)-O-(3,6-di-O-benzyl-2-phthalimido-β-D-glucopyranosyl)-(1→4)-[O-2,3,4-tri-O-(methoxybenzyl)-α-L-fucopyranosyl-(1→6)]-3-O-benzyl-2-deoxy-2-phthalimido-β-D-glucopyranosylazide ***3***

Octasaccharide azide **1** (30.0 mg, 10.6 μmol), thiofucoside **2** (60.4 mg, 106.1 μmol) and ground molecular sieves 4 Å (110 mg) were suspended in dry dichloromethane (446 μL) and DMF (234 μL) under an argon atmosphere. Copper(II)bromide (61.6 mg, 275.8 μmol) and tetrabutylammonium bromide (51.3 mg, 159.1 μmol) were added. After stirring for 4 d (TLC: dichloromethane/methanol 20:1) the reaction was diluted with dry dichloromethane and was quenched with triethylamine (37 μL, 264.2 μmol) and ethanethiol (20.4 μL, 275.9 μmol). The suspension was filtered over celite and extracted with 2 M potassium hydrogen carbonate. The organic layer was dried over $MgSO_4$, concentrated in vacuum and dried under high vacuum. The residue was purified by flash chromatography (cyclohexane/acetone 2:1) to furnish the crude fucosylated nonasaccharide as a mixture of anomers (α/β = 5.4:1, 32.5 mg). The mixture was purified by RP-HPLC (Macherey-Nagel Nucleogel® RP 100-10; 25 x 300 mm; methanol; flow rate: 10 mL/min; detection: 214, 254, 280 nm) furnishing 25.1 mg of α-anomer **3** (7.53 μmol, 71.0 %). R_f = 0.50 (dichloromethane/methanol, 20:1); $[\alpha]_D^{22}$ = -23.2 (dichloromethane), $C_{153}H_{171}F_9N_8O_{65}$ (3333.0), ESI-MS (100 % acetonitrile): $M_{calcd.}$ = 3331.0 M_{found} = 3349.8 $(M+H_3O)^+$.

^{1}H-NMR (500 MHz, [D6]-DMSO): δ = 9.77 (d, $J_{NH,2}$ = 8.4 Hz, 1H, NH), 9.57 (d, $J_{NH,2}$ = 8.4 Hz, 1H, NH), 9.48 (d, $J_{NH,2}$ = 8.4 Hz, 1H, NH), 7.89-7.55 (m, 8H, NPht), 7.38-7.16 (m, 14H, Ar), 6.95-6.76 (m, 13H, Ar), 5.40-5.13 (m, 7H, H-1^2, H-3^B, H-$3^{5'}$, H-$4^{4'}$, H-2^3, H-1^1, H-3^5), 5.10-4.86 (m, 6H, H-4^4, H-4^B, H-$3^{4'}$, H-3^4, H-1^4, CH_2O), 4.86-4.23 (m, 20H, H-$1^{4'}$, CH_2O, H-$4^{5'}$, H-4^5, CH_2O, H-1^5, H-1^B, CH_2O, CH_2O, CH_2O, CH_2O, CH_2O, H-1^F, H-1^3, H-$1^{5'}$, CH_2O, CH_2O H-$6a^5$, CH_2O, CH_2O), 4.23-3.78 (m, 23H, H-3^1, H-$2^{4'}$, H-3^2, H-2^B, H-$6a^4$, H-4^1, H-4^2, H-2^4, H-$6a^B$, H-2^2, H-$6b^5$, H-5^4, H-$6a^{5'}$, H-$6a^{4'}$, H-$5^{4'}$, H-2^5, H-$6b^4$, H-$2^{5'}$, H-5^5, H-4^3,

H-5^{B}, H-2^{1}, H-6b^{B}), 3.78-3.40 (m, 21H, OCH_3, H-5^{F}, H-6a^{2}, H-6a^{3}, H-3^{F}, H-6b^{3}, H-2^{F}, H-5^{2}, H-6b^{2}, H-4^{F}, H-6b$^{5'}$, H-3^{3}, H-6a^{1}), 3.25-3.02 (m, 3 H, H-6b^{1}, H-5^{3}, H-5$^{5'}$), 2.09-1.77 (m, 48H, OAc), 0.92 (d, $J_{5,6}$ = 6.4 Hz, 3 H, H-6^{F}),

^{13}C-NMR (125 MHz, [D6]-DMSO): δ = 170.1, 170.0, 169.9, 169.8, 169.7, 169.6, 169.4, 169.3, 169.2, 169.1 (C=O OAc), 167.6, 167.3 (C=O NPht), 158.6, 158.5 (C-4 Mpm), 156.6 (q, $^{2}J_{C,F}$ = 36.1 Hz, C=O TFAc), 156.4 (q, $^{2}J_{C,F}$ = 36.2 Hz, C=O TFAc), 138.1, 138.0 (C-1 Ar), 134.9, 134.5 (C-4/5 NPht), 130.9, 130.8, 130.7, 130.5 (C-1 Mpm, C-1/2 NPht), 129.5, 129.4, 129.1, 128.3, 128.1, 128.0, 127.8, 127.7, 127.5, 126.6, 126.4 (C-2/6 Mpm, Ar), 123.5, 123.4 (C-3/6 NPht), 115.8 (q, $^{1}J_{C,F}$ = 288.5 Hz, CF_3), 113.2 (q, $^{1}J_{C,F}$ = 288.5 Hz, CF_3), 113.5, 113.4 (C-3/5 Mpm), 99.5 (C-1^{B}, $^{1}J_{C\text{-}1,H\text{-}1}$ = 168.1 Hz), 98.4 (C-1^{5}, $^{1}J_{C\text{-}1,H\text{-}1}$ = 164.2 Hz), 97.9 (C-1$^{5'}$, $^{1}J_{C\text{-}1,H\text{-}1}$ = 166.5 Hz), 97.7 (C-1^{4}, $^{1}J_{C\text{-}1,H\text{-}1}$ = 176.6 Hz), 96.8 (C-1^{F}, $^{1}J_{C\text{-}1,H\text{-}1}$ = 175.3 Hz), 96.4 (C-1^{2}, $^{1}J_{C\text{-}1,H\text{-}1}$ = 169.6 Hz), 96.4 (C-1^{3}, $^{1}J_{C\text{-}1,H\text{-}1}$ = 163.5 Hz), 96.2 (C-1$^{4'}$, $^{1}J_{C\text{-}1,H\text{-}1}$ = 174.3 Hz), 84.3 (C-1^{1}, $^{1}J_{C\text{-}1,H\text{-}1}$ = 168.0 Hz), 78.0 (C-3^{F}), 77.3 (C-4^{F}), 77.0 (C-4^{2}), 76.7 (C-3^{2}), 75.4 (C-3^{3}), 75.2 (C-4^{1}), 75.2 (C-3^{1}), 74.1 (C-5^{2}), 73.9 (CH_2O), 73.7 (CH_2O), 73.7 (C-4^{3}), 73.7 (C-5^{3}), 73.5 (CH_2O), 73.4 (C-2^{F}), 73.3 (C-2^{4}), 72.8 (C-2$^{4'}$), 72.5 (CH_2O), 71.6 (CH_2O), 71.4 (C-3^{5}), 71.2 (C-3$^{5'}$), 71.0 (C-5^{5}), 70.9 (CH_2O), 70.8(C-3^{B}), 70.8 (C-5^{B}), 70.6 (C-5$^{5'}$), 70.2(C-2^{3}), 70.0(C-3$^{4'}$), 69.3 (C-4^{5}), 68.6 (C-3^{4}), 68.5 (C-5^{4}), 68.1 (C-4$^{5'}$), 67.8 (C-5$^{4'}$), 67.6 (C-6^{2}), 67.6 (C-4^{B}), 66.3 (C-6^{3}), 65.6 (C-5^{F}), 65.6 (C-4^{4}), 64.7 (C-4$^{4'}$), 63.5 (C-6^{1}), 62.9 (C-6^{B}), 62.7 (C-6$^{4'}$), 61.9 (C-6^{4}), 61.5 (C-6$^{5'}$), 60.8 (C-6^{5}), 55.5 (C-2^{2}), 55.0 (OCH_3), 55.0 (OCH_3), 54.9 (C-2^{1}), 54.8 (OCH_3), 54.7 (C-2^{B}), 54.0 (C-$^{5'}$), 53.9 (C-5^{5}), 20.7, 20.6, 20.5, 20.4, 20.3, 20.2, 20.1, 20.0 (OAc).

O-(2-Acetamido-2-deoxy-β-D-glucopyranosyl)-(1→2)-O-(α-D-mannopyranosyl)-(1→3)-[O-(2-acetamido-2-deoxy-β-D-glucopyranosyl)-(1→4)]-{O-(2-acetamido-2-deoxy-β-D-glucopyranosyl)-(1→2)-O-(α-D-mannopyranosyl)-(1→6)}-O-(β-D-mannopyranosyl)-(1→4)-O-(2-acetamido-2-deoxy-β-D-glucopyranosyl)-(1→4)-[O-(α-L-fucopyranosyl-(1→6)]-O-2-acetamido-2-deoxy-D-glucopyranose ***4***

The fucosylated nonasaccharide azide **3** (102 mg, 30.6 µmol) was dissolved in *n*-butanol (6.12 mL) and ethylenediamine (1.43 mL, 21.4 mmol) and stirred for 18 h at 90 °C. The volatiles were evaporated in vacuo and residual reagents were removed by addition of toluene and azeotropic distillation (3x). After drying in high vacuum the residue was dissolved in acetic anhydride/pyridine (1:2, 5.5 mL) and kept at room temperature for 16 h. The volatiles were

evaporated in vacuo followed by the addition of toluene and azeotropic distillation (3x). The residue was dried under high vacuum and subsequently dissolved in methylamine (40 % in water, 6.1 mL) and kept at room temperature for 20 h. The mixture was concentrated in vacuum and lyophilized from water. The residue was purified by solid-phase extraction (Waters Sep-Pak® C18 Classic, 3 x 330 mg, loading with water and stepwise elution with 7.5 mL of 10, 20, 30, 40, 50, 60, 70, 100 % acetonitrile/water, product eluted at 40 and 50 % acetonitrile/water) (63 mg, 27.1 µmol, 88.6 %).

A solution of the residue (52.0 mg, 22.4 µmol) in 2.6 mL of dry MeOH was treated with DIPEA (60 µL, 344.5 µmol) and 1,3-propanedithiol (161 µL, 1.60 mmol) under an argon atmosphere. After 5 h the mixture was dried under high vacuum and lyophilized from 2 mL of water. The residue was purified by solid-phase extraction (Waters Sep-Pak® C18 Classic, 1 x 330 mg, loading with water and stepwise elution elution with 5 mL of 10, 20, 30, 40, 50, 60, 70, 100 % acetonitrile/water, elution of glycosylamine at 30 and 40 % acetonitrile). The lyophilized glycosylamine (46.6 mg, 20.3 µmol) was dissolved in water (570 µL) and acetic acid (570 µL). After 20 h the mixture was lyophilized. The benzylated hemiacetal was purified by gel filtration (Sephadex® LH 20, 25 x 1000 mm, methanol, 1 mL/min, retention time 230 min) (39.2 mg, 17.1 µmol, 76.3 %).

Palladium(II) oxide hydrate (64.9 mg, 530 µmol) was suspended in dry methanol/acetic acid (10:1, 6.3 mL) under a hydrogen atmosphere. The mixture was stirred at room temperature for 15 min. The benzylated hemiacetal (39.0 mg, 17.0 µmol) was dissolved in dry methanol/acetic acid (10:1, 6.3 mL) and was added to the suspension of the palladium catalyst. The mixture was stirred under a hydrogen atmosphere for 66 h. After centrifugation the supernatant was decanted. The catalyst was washed four times with 10 % acetic acid in methanol. The combined organic layers were concentrated and the residue was purified by gel filtration (Bio-Gel P-4 Gel Fine, 25 × 750 mm, water, 1.5 mL/min, retention time 110 min). The fractions containing **4** were dried and the residue was purified by solid-phase extraction (Waters Sep-Pak® C18 Classic, 330 mg, loading with water and stepwise elution elution with 0, 5, 10, 20, 30, 40, 50, 60, 70, 100 % acetonitrile/water, elution of product at 0 and 5 % acetonitrile/water) to furnish the fucosylated nonasaccharide **4** (20.2 mg, 12.1 µmol, 71.2 %). R_f = 0.43 (*i*-propanol/1 M ammonium acetate 2:1), $[\alpha]_D^{25}$ = -30.0 (0.1, water), $C_{64}H_{107}N_5O_{45}$ (1666.54), ESI-MS (water): $M_{calcd.}$ = 1665.62; M_{found} = 856.05 $(M+2Na)^{2+}$, 1689.15 $(M+Na)^{+}$.

^{1}H-NMR (500 MHz, D_2O): δ = 9.97 (m, 1H, H-1^1 α), 4.86 (d, $J_{1,2}$ < 1 Hz, 1H, H-1^4), 4.81 (d, $J_{1,2}$ < 1 Hz, 1H, H-$1^{4'}$), 4.69 (m, 1H, H-1^F), 4.49-4.48 (m, 2H, H-1^3, H-1^1 β), 4.46 (d, $J_{1,2}$ =

7.6 Hz, 1H, H-1^{2}), 4.36-4.34 (m, 2H, H-$1^{5'}$, H-1^{5}), 4.26 (d, $J_{1,2}$ = 7.7 Hz, 1H, H-1^{B}), 4.05 (m, 1H, H-2^{4}), 3.97 (m, 1H, H-2^{3}), 3.94 (m, 1H, H-$2^{4'}$), 3.93-3.84 (m, 2H, H-5^{F}, H-4^{3}), 3.82-3.62 (m, 15H, H-3^{F}, H-$6a^{B}$, H-$6a^{4}$, H-$6a^{4'}$, H-$6a^{5'}$, H-3^{4}, H-$6a^{3}$, H-$6b^{3}$, H-3^{1}, H-2^{1} α-hemiacetal, H-$6a^{5}$, H-3^{3}, H-$6a^{2}$, H-$3^{4'}$, H-$6a^{1}$), 3.61-3.45 (m, 17H, H-4^{F}, H-2^{F}, H-2^{2}, H-$6b^{5'}$, H-4^{1}, H-3^{2}, H-4^{2}, H-$6b^{5}$, H-$2^{5'}$, H-5^{4}, H-$6b^{1}$, H-2^{1} β-hemiacetal, H-2^{B}, H-$6b^{B}$, H-2^{5}, H-5^{1}, H-$6b^{2}$), 3.44-3.33 (m, 7H, H-$5^{4'}$, H-$6b^{4}$, H-$6b^{4'}$, H-$3^{5'}$, H-5^{2}, H-3^{B}, H-5^{3}), 3.32-3.18 (m, 8H, H-$4^{5'}$, H-3^{5}, H-4^{4}, H-$4^{4'}$, H-4^{5}, H-$5^{5'}$, H-5^{B}, H-5^{5}), 3.06 (dd, $J_{3,4}$ = $J_{4,5}$ = 8.8 Hz, 1H, H-4^{B}), 1.90, 1.87, 1.86, 1.85, 1.84 (5 s, 15H, NAc), 1.03-1.00 (m, 3H, H-6^{F}),

^{13}C-NMR (125 MHz, D_2O): δ = 176.2, 176.2, 176.1, 176.0, 176.0, 175.9 (C=O NAc), 102.7 (C-1^{2} β, $J_{C-1,H-1}$ = 165.1 Hz), 102.2 (C-1^{B} β, $J_{C-1,H-1}$ = 163.4 Hz), 101.7 (C-1^{3} β, $J_{C-1,H-1}$ = 161.7 Hz), 101.5 (C-$1^{5'}$ β, $J_{C-1,H-1}$ = 163.9 Hz), 101.5 (C-1^{4} α, $J_{C-1,H-1}$ = 173.7 Hz), 101.2 (C-1^{5} β, $J_{C-1,H-1}$ = 162.0 Hz), 101.1 (C-1^{F} α, $J_{C-1,H-1}$ = 171.4 Hz; α-hemiacetal), 100.9 (C-1^{F} β, $J_{C-1,H-1}$ = 171.4 Hz; β-hemiacetal), 99.2 (C-$1^{4'}$ α, $J_{C-1,H-1}$ = 172.6 Hz), 96.5 (C-1^{1} β, $J_{C-1,H-1}$ = 164.1 Hz), 92.0 (C-1^{1} α, $J_{C-1,H-1}$ = 172.6 Hz), 80.7 (C-4^{1}), 80.3 (C-4^{2}), 80.2 (C-3^{3}), 78.3 (C-5^{B}), 78.1 (C-2^{4}), 77.7 (C-$2^{4'}$), 77.4 (C-5^{5}), 77.3 (C-$5^{5'}$), 76.1 (C-5^{2}), 75.8 (C-5^{3}), 75.1, 75.0, 74.9 (C-3^{B}, C-3^{5}, C-$3^{5'}$, C-5^{4}, C-$5^{4'}$), 74.5 (C-5^{1}), 73.5 (C-3^{2}), 73.4 (C-4^{F}), 73.2 (C-4^{3}), 72.7 (C-4^{B}), 71.9 (C-2^{3}), 71.4 (C-$4^{5'}$), 71.3 (C-4^{5}), 71.0 (C-3^{4}), 71.0 (C-$3^{4'}$), 70.9 (C-3^{F}), 70.7 (C-3^{1}), 69.8 (C-2^{F}), 69.1 (C-4^{4}), 68.9 (C-$4^{4'}$), 68.6 (C-6^{1}), 68.4 (C-5^{F}), 68.2 (C-6^{2}), 67.0 (C-6^{3}), 63.5 (C-6^{4}), 63.4 (C-$6^{4'}$), 63.2 (C-6^{B}), 62.2 (C-$6^{5'}$), 61.4 (C-6^{5}), 57.7 (C-2^{B}), 56.9, 56.8, 56.7 (C-2^{1} β-hemiacetal, C-2^{2}, C-2^{5}, C-$2^{5'}$), 55.3 (C-2^{1} α-hemiacetal), 24.0, 24.0, 24.0, 23.8, 23.8, 23.5 (CH_3 NAc), 17.0 (C-6^{F}; β-hemiacetal), 16.9 (C-6^{F}; α-hemiacetal).

Synthesis of trigalactoside 6

O-(β-D-Galactopyranosyl)-(1→4)-O-(2-acetamido-2-deoxy-β-D-glucopyranosyl)-(1→2)-O-(α-D-mannopyranosyl)-(1→3)-[O-(β-D-galactopyranosyl)-(1→4)-O-(2-acetamido-2-deoxy-β-D-glucopyranosyl)-(1→4)]-{O-(β-D-galactopyranosyl)-(1→4)-O-(2-acetamido-2-deoxy-β-D-glucopyranosyl)-(1→2)-O-(α-D-mannopyranosyl)-(1→6)}-O-(β-D-mannopyranosyl)-(1→4)-O-(2-acetamido-2-deoxy-β-D-glucopyranosyl)-(1→4)-[O-(α-L-fucopyranosyl-(1→6)]-2-acetamido-2-deoxy-D-glucopyranose ***6***

Nonasaccharide **4** (3.68 mg, 2.21 µmol) was dissolved in 242 µL of Tris hydrochloride (50 mM, pH 7.5) containing UDP-Gal (5.58 mg, 8.86 µmol), 10 µg of bovine serum albumin, 1.20 µmol of $MnCl_2$, 174 mU of bovine GlcNAc β-1,4-galactosyltransferase (EC 2.4.1.22) and 24 U of calf intestinal alkaline phosphatase (EC 3.1.3.1). The reaction mixture was incubated for 4 d at 37 °C to furnish a mixture of digalactoside **5** and trigalactoside **6**, which was lyophilized. The dried residue was purified by gel filtration (Sephadex G25 Fine, 8 x 280 mm, 0.1 M ammonium hydrogen carbonate) yielding a mixture of **4** and **5** (4.47 mg). The mixture was dissolved in 115 µL of Tris hydrochloride (50 mM, pH 7.5) containing UDP-Gal (5.79 mg, 9.19 µmol), 17 µg of bovine serum albumin, 0.58 µmol of $MnCl_2$, 315 mU of bovine GlcNAc β-1,4-galactosyltransferase (EC 2.4.1.22) and 44 U of calf intestinal alkaline phosphatase (EC 3.1.3.1). After incubation for 66 h at 37 °C the reaction mixture still contained small amounts of digalactoside **5** and more UDP-Gal (5.79 mg, 9.19 µmol), bovine GlcNAc β-1,4-galactosyltransferase (315 mU) and calf intestinal alkaline phosphatase (10 U) was added. The reaction mixture was incubated for 2 d at 37 °C leading to complete conversion to trigalactoside **6**. The mixture was lyophilized and the dried residue was purified by gel filtration (Hi Load Superdex 30, 16 × 600 mm, 0.1 M ammonium hydrogen carbonate, flow rate 0.75 mL/min, retention time 101 min) to furnish the trigalactoside **6** (3.54 mg, 1.64 µmol, 74.2 %). R_f (digalactoside **5**) = 0.34 (*i*-propanol/30 % NH_4OH / water 2:3:1), R_f (trigalactoside **6**) = 0.24 (*i*-propanol/28-30 % NH_4OH in Wasser/ water 2:3:1), $[\alpha]_D^{24}$ = -15.0 (0.34, water), $C_{82}H_{137}N_5O_{60}$ (2152.96), MALDI-TOF: $M_{calcd.}$ = 2151.78; M_{found} = 2175 $(M+Na)^+$, 2191 $(M+K)^+$.

a)

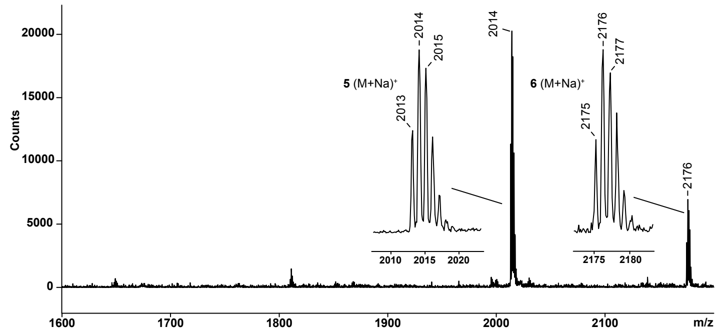

b)

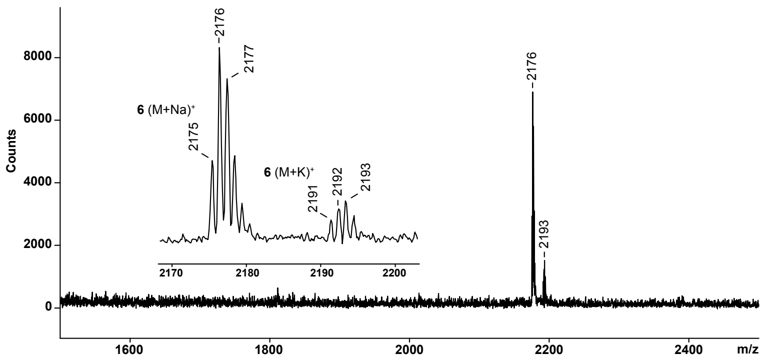

***Scheme S2**: a) MALDI-TOF-MS of a mixture of **5** and **6** obtained by galactosylation of **4** [9 mM] for 3 days; b) MALDI-TOF-MS of trigalactosylated **6**.*

^{1}H-NMR (360 MHz, D_2O): δ = 4.97 (m, 1H, H-1^{1}α), 4.85 (d, $J_{1,2}$ < 1 Hz, 1H, H-1^4), 4.82 (d, $J_{1,2}$ < 1 Hz, 1H, H-1$^{4'}$), 4.71-4.69 (m, 1H, H-1^F), 4.50-4.48 (m, 2H, H-1^3, H-1^{1}β), 4.46 (d, $J_{1,2}$ = 8.4 Hz, 1H, H-1^2), 4.42-4.37 (m, 2H, H-1^5, H-1$^{5'}$), 4.29-4.22 (m, 4H, H-1^B, H-1^G, H-1$^{G'}$, H-1^{GB}), 4.07 (m, 1H, H-2^4), 3.99 (m, 1H, H-2^3), 3.95-3.84 (m, 4H, H-2$^{4'}$, H-5^F, H-4^3, H-6a^B), 3.83-3.50 (m, 45H, H-3^F, H-6a^5, H-6a$^{5'}$, H-6a^4, H-6a$^{4'}$, H-4^G, H-4$^{G'}$, H-4^{GB}, H-6a^3, H-6b^3, H-3^4, H-2^{1}α, H-6b^5, H-3^3, H-6a^2, H-6b$^{5'}$, H-3$^{4'}$, H-6a^1, H-2^2, H-3^1, H-4^F, H-2^F, H-2^5, H-4^1, H-6b^B, H-6a^G, H-6b^G, H-6a$^{G'}$, H-6b$^{G'}$, H-6a^{GB}, H-6b^{GB}, H-3^5, H-4^2, H-3^2, H-2$^{5'}$, H-3^B, H-3$^{5'}$, H-4^5, H-4$^{5'}$, H-6b^1, H-2^B, H-5^1, H-6b^2, H-5^4, H-2^{1}β), 3.49-3.39 (m, 10H, H-3^G, H-3$^{G'}$, H-3^{GB}, H-5^G, H-5$^{G'}$, H-5^{GB}, H-5^5, H-5$^{4'}$, H-6b^4, H-6b$^{4'}$), 3.38-3.24 (m, 10H, H-5^2, H-5^B, H-5$^{5'}$, H-2^G, H-2$^{G'}$, H-2^{GB}, H-5^3, H-4^B, H-4^4, H-4$^{4'}$), 1.90, 1.87, 1.85 (3 s, 9H, NAc), 1.84 (s, 6H, NAc), 1.03-1.00 (m, 3H, H-6^F),

^{13}C-NMR (90.6 MHz, D_2O, δ determined from an HSQC-Spektrum): δ = 104.6 (C-1^{GB}β, $J_{C-1,H-1}$ = 161.4 Hz), 104.4 (C-1^{G}β, $J_{C-1,H-1}$ = 161.4 Hz), 104.4 (C-1$^{G'}$β, $J_{C-1,H-1}$ = 161.4 Hz), 102.5 (C-1^{2}β, $J_{C-1,H-1}$ = 164.0 Hz), 101.9 (C-1^{B}β, $J_{C-1,H-1}$ = 162.8 Hz), 101.5 (C-1^{3}β, $J_{C-1,H-1}$ = 160.9 Hz), 101.4 (C-1^{4}α, $J_{C-1,H-1}$ = 170.9 Hz), 101.1 (C-1^{5}β, $J_{C-1,H-1}$ = 162.4 Hz), 100.9 (C-1^{F}α, $J_{C-1,H-1}$ = 170.9 Hz), 100.8 (C-1$^{5'}$β, $J_{C-1,H-1}$ = 161.4 Hz), 99.0 (C-1$^{4'}$α, $J_{C-1,H-1}$ = 170.6 Hz), 96.3 (C-1^{1}β, $J_{C-1,H-1}$ = 161.7 Hz), 91.8 (C-1^{1}α, $J_{C-1,H-1}$ = 170.8 Hz), 81.7 (C-4^B), 80.3 (C-3^3), 80.0 (C-4^1), 80.0 (C-4^2), 80.0 (C-4^5), 80.0 (C-4$^{5'}$), 77.8 (C-2^4), 77.6 (C-2$^{4'}$), 76.6 (C-3^B), 76.6 (C-3^5), 76.6 (C-3$^{5'}$), 76.5 (C-5^B), 75.9 (C-5^2), 75.9 (C-5^3), 75.9 (C-5^5), 75.9 (C-5$^{5'}$), 74.8 (C-5$^{4'}$), 73.9 (C-3^G), 73.9 (C-3$^{G'}$), 73.9 (C-3^{GB}), 73.9 (C-5^G), 73.9 (C-5$^{G'}$), 73.9 (C-5^{GB}), 73.9 (C-5^4), 73.5 (C-5^1), 73.3 (C-3^2), 73.2 (C-4^F), 72.9 (C-4^3), 72.3 (C-2^G), 72.3 (C-2$^{G'}$), 72.3 (C-2^{GB}), 71.7 (C-2^3), 70.7 (C-3^1), 70.7 (C-3^4), 70.7 (C-3$^{4'}$), 70.6 (C-3^F), 70.0 (C-4^G), 70.0 (C-4$^{G'}$), 70.0 (C-4^{GB}), 69.7 (C-2^F), 68.9 (C-4^4), 68.8 (C-4$^{4'}$), 68.5 (C-6^1), 68.4 (C-6^2), 68.2 (C-5^F), 66.5 (C-6^3), 63.2 (C-6^4), 63.2 (C-6$^{4'}$), 62.3 (C-6^B), 62.2 (C-6^G), 62.2 (C-6$^{G'}$), 62.2 (C-6^{GB}), 61.4 (C-6^5), 61.3 (C-6$^{5'}$), 57.6 (C-2^{1}β), 56.6 (C-2^B), 56.5 (C-2^2), 56.3 (C-2^5), 56.3 (C-2$^{5'}$), 55.1 (C-2^{1}α), 23.8, 23.7, 23.6 (NAc), 16.7 (C-6^F).

Synthesis of digalactoside 5

O-(β-D-Galactopyranosyl)-(1→4)-O-(2-acetamido-2-deoxy-β-D-glucopyranosyl)-(1→2)-O-(α-D-mannopyranosyl)-(1→3)-[O-(2-acetamido-2-deoxy-β-D-glucopyranosyl)-(1→4)]-{O-(β-D-galactopyranosyl)-(1→4)-O-(2-acetamido-2-deoxy-β-D-glucopyranosyl)-(1→2)-O-(α-D-mannopyranosyl)-(1→6)}-O-(β-D-mannopyranosyl)-(1→4)-O-(2-acetamido-2-deoxy-β-D-glucopyranosyl)-(1→4)-[O-(α-L-fucopyranosyl-(1→6)]-2-acetamido-2-deoxy-D-gluco-pyranose ***5***

Nonasaccharide **4** (2.10 mg, 1.26 μmol) was dissolved in 504 μL of sodium cacodylate (20 mM, pH 7.5) containing UDP-Gal (2.38 mg, 3.78 μmol), 0.38 mg of bovine serum albumin, 0.52 μmol of $MnCl_2$, 38 mU of bovine GlcNAc β-1,4-galactosyltransferase and 14.3 U of calf intestinal alkaline phosphatase. The reaction mixture was incubated for 18 h at 37 °C and lyophilized. The residue was purified by gel filtration (Sephadex G25 fine, 8 x 280 mm, 0.1 M ammonium hydrogen carbonate) and lyophilized. The crude digalactoside **5** (2.56 mg) was purified by gel filtration (Hi Load Superdex 30, 16 × 600 mm, 0.1 M ammonium hydrogen carbonate, flow rate 0.75 mL/min, retention time 102 min) to furnish the digalactoside **5** (1.96 mg, 0.98 μmol, 77.8 %). R_f (nonasaccharide **4**) = 0.29 (*i*-propanol/30 % NH_4OH/water 3:3:1), R_f (digalactoside **5**) = 0.11 (*i*-propanol/30 % NH_4OH/ water 3:3:1), $[\alpha]_D^{23}$ = -32.7 (0.10, water), $C_{76}H_{127}N_5O_{55}$ (1990.82), MALDI-TOF: $M_{calcd.}$ = 1989.73; M_{found} = 2013 $(M+Na)^+$,

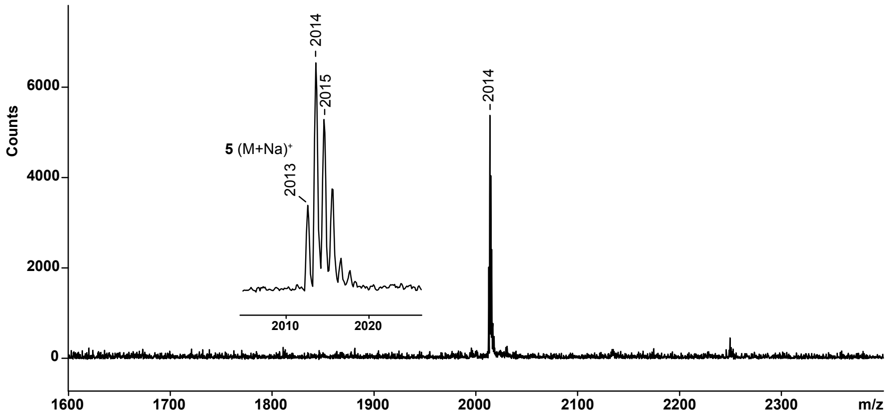

Scheme S3: *MALDI-TOF-MS of digalactosylated* ***5***.

^{1}H-NMR (500 MHz, D_2O): δ = 4.97 (m, 1H, H-1^1α), 4.86 (d, $J_{1,2}$ < 1 Hz, 1H, H-1^4), 4.82 (d, $J_{1,2}$ < 1 Hz, 1H, H-$1^{4'}$), 4.73-4.69 (m, 1H, H-1^F), 4.50-4.48 (m, 2H, H-1^3, H-1^1β), 4.46 (d, $J_{1,2}$ = 7.7 Hz, 1H, H-1^2), 4.40-4.37 (m, 2H, H-1^5, H-$1^{5'}$), 4.28-4.26 (m, 3H, H-1^G, H-$1^{G'}$, H-1^B),

4.06 (m, 1H, H-2^4), 3.98 (m, 1H, H-2^3), 3.96-3.85 (m, 3H, H-$2^{4'}$, H-5^F, H-4^3), 3.83-3.63 (m, 17H, H-3^F, H-$6a^B$, H-$6a^5$, H-$6a^4$, H-$6a^{4'}$, H-4^G, H-$4^{G'}$, H-3^4, H-$6a^3$, H-$6b^3$, H-$2^1\alpha$, H-3^3, H-$6a^2$, H-$6b^5$, H-$6a^{5'}$, H-$6a^1$, H-$3^{4'}$), 3.62-3.31 (m, 39, H-2^2, H-3^1, H-4^F, H-2^F, H-4^1, H-2^5, H-3^5, H-3^2, H-$6a^G$, H-$6a^{G'}$, H-$6b^G$, H-$6b^{G'}$, H-$3^{5'}$, H-4^2, H-4^5, H-$4^{5'}$, H-$6b^1$, H-$6b^{5'}$, H-$2^{5'}$, H-5^1, H-5^4, H-$2^1\beta$, H-$6b^2$, H-2^B, H-$6b^B$, H-3^G, H-$3^{G'}$, H-5^G, H-$5^{G'}$, H-$5^{4'}$, H-5^5, H-$6b^4$, H-$6b^{4'}$, H-5^2, H-3^B, H-$5^{5'}$, H-2^G, H-$2^{G'}$, H-5^3), 3.30-3.25 (m, 2H, H-$4^{4'}$, H-4^4), 3.20 (m, 1H, H-5^B), 3.06 (dd, $J_{3,4}$ = $J_{4,5}$ = 8.8 Hz, 1H, H-4^B), 1.90, 1.87, 1.86, 1.84 (4 s, 15H, NAc), 1.03-1.00 (m, 3H, H-6^F),

^{13}C-NMR (125 MHz, D_2O): δ = 176.2, 176.1, 176.1, 176.0 (C=O NAc), 104.6, 104.5 (C-$1^G\beta$, C-$1^{G'}\beta$, $J_{C\text{-}1,H\text{-}1}$ = 164.1 Hz), 102.7 (C-$1^2\beta$, $J_{C\text{-}1,H\text{-}1}$ = 167.6 Hz), 102.2 (C-$1^B\beta$, $J_{C\text{-}1,H\text{-}1}$ = 161.5 Hz), 101.7 (C-$1^3\beta$, $J_{C\text{-}1,H\text{-}1}$ = 161.3 Hz), 101.5 (C-$1^4\alpha$, $J_{C\text{-}1,H\text{-}1}$ = 171.9 Hz), 101.3 (C-$1^5\beta$, $J_{C\text{-}1,H\text{-}1}$ = 162.1 Hz), 101.2 (C-$1^F\alpha$, $J_{C\text{-}1,H\text{-}1}$ = 170.6 Hz; α-GlcNAc), 101.0 (C-$1^{5'}\beta$, $J_{C\text{-}1,H\text{-}1}$ = 162.3 Hz), 100.9 (C-$1^F\alpha$, $J_{C\text{-}1,H\text{-}1}$ = 170.6 Hz; β-GlcNAc), 99.3 (C-$1^{4'}\alpha$, $J_{C\text{-}1,H\text{-}1}$ = 171.4 Hz), 96.5 (C-$1^1\beta$, $J_{C\text{-}1,H\text{-}1}$ = 162.5 Hz), 92.1 (C-$1^1\alpha$, $J_{C\text{-}1,H\text{-}1}$ = 173.9 Hz), 80.7, 80.4, 80.2, 80.0, 79.7 (C-3^3, C-4^1, C-4^2, C-4^5, C-$4^{5'}$), 78.3 (C-5^B), 78.0 (C-2^4), 77.9 (C-$2^{4'}$), 76.9 (C-3^5), 76.9 (C-$3^{5'}$), 76.3, 76.2, 75.9, 75.5, 75.1, 75.0, 74.9 (C-3^B, C-5^2, C-5^3, C-5^4, C-$5^{4'}$, C-5^5, C-$5^{5'}$), 74.1, 74.0, 73.8, 73.6, 73.4, 73.2 (C-3^G, C-$3^{G'}$, C-3^2, C-4^F, C-5^1, C-5^G, C-$5^{G'}$), 73.0 (C-4^3), 72.7 (C-4^B), 72.6, 72.5 (C-2^G, C-$2^{G'}$), 71.9 (C-2^3), 71.1, 70.9, 70.7 (C-3^1, C-3^4, C-$3^{4'}$, C-3^F), 70.1 (C-4^G), 70.1 (C-$4^{G'}$), 69.8 (C-2^F), 69.2 (C-$4^{4'}$), 68.9 (C-4^4), 68.7 (C-6^1), 68.7 (C-6^2), 68.4 (C-5^F), 66.8 (C-6^3), 63.5 (C-6^4), 63.5 (C-$6^{4'}$), 63.2 (C-6^B), 62.6, (C-6^G), 62.6 (C-$6^{G'}$), 61.6 (C-6^5), 61.5 (C-$6^{5'}$), 57.7 (C-2^B), 56.8 (C-2^2), 56.8 (C-$2^1\beta$), 56.4 (C-$2^{5'}$), 56.4 (C-2^5), 55.4 (C-$2^1\alpha$), 24.1, 24.0, 23.7 (NAc), 17.0 (C-6^F; β-GlcNAc), 16.9 (C-6^F; α-GlcNAc).

Enzymatic galactosylation of 7-11

A master mix (total volume 50 μL) was prepared containing 29 μL of 5 mM Tris hydrochloride buffer (pH 7.5), 3 μL of bovine serum albumin (20 mg/mL), 0.6 μL of $MnCl_2$ (100 mM), 0.6 μL of NaN_3 (100 mM), 0.6 μL of alkaline phosphatase (1 U/μL) and 16.3 μL of bovine GlcNAc β-1,4-galactosyltransferase (10 U/mL). The glycan azides **7-11** (100 nmol, 1 eq., final concentration 2 mM) were dissolved in 5 mM Tris hydrochloride buffer (pH 7.5), UDP-Gal (50 mg/mL) in 5 mM Tris hydrochloride buffer (pH 7.5) and 5 μL of the master mix

were added (volumes see Table S1). The reactions were incubated for 24 h at 37 °C leading to complete conversion to the galactosylated glycan azides **12-16**.

Table S1.

Compound	5 mM tris buffer (pH 7.5)	UDP-Gal in 5 mM tris buffer (pH 7.5)
**7** (154 µg, 2 mM)	40 µL	5 µL (0.42 µmol, 4 eq.)
**8** (169 µg, 2 mM)	40 µL	5 µL (0.42 µmol, 4 eq.)
**9** (175 µg, 2 mM)	37.5 µL	7.5 µL (0.64 µmol, 6 eq.)
**10** (175 µg, 2 mM)	37.5 µL	7.5 µL (0.64 µmol, 6 eq.)
**11** (195 µg, 2 mM)	34.5 µL	10.5 µL (0.84 µmol, 8 eq.)

12: $C_{70}H_{116}N_8O_{50}$ (1868.68), ESI-MS: $M_{calc.}$ = 1891.67 $(M+Na)^+$; M_{found} = 1891.93 $(M+Na)^+$,
13: $C_{76}H_{126}N_8O_{54}$ (2014.74), ESI-MS: $M_{calc.}$ = 2037.73 $(M+Na)^+$; M_{found} = 2037.92 $(M+Na)^+$,
14: $C_{84}H_{139}N_9O_{60}$ (2233.81), ESI-MS: $M_{calc.}$ = 2256.80 $(M+Na)^+$; M_{found} = 2256.96 $(M+Na)^+$,
15: $C_{84}H_{139}N_9O_{60}$ (2233.81), ESI-MS: $M_{calc.}$ = 2256.80 $(M+Na)^+$; M_{found} = 2256.96 $(M+Na)^+$,
16: $C_{98}H_{162}N_{10}O_{70}$ (2598.94), ESI-MS: $M_{calc.}$ = 2621.93 $(M+Na)^+$; M_{found} = 2622.27 $(M+Na)^+$.

Enzymatic galactosylation of 12-16

After complete conversion to the galactosylated glycan azides **12-16** 25 µL of each reaction mixture (50 nmol, 1 eq.) were lyophilized. To the dried residue were added 0.28 µL of 5 mM Tris hydrochloride (pH 7.5), 0.19 µL (4.2 eq.) of UDP-Gal (750 mg/mL) in 5 mM Tris hydrochloride (pH 7.5) and 2.34 µL of master mix (as above). The reactions (final concentrations of N-glycans **12-16** = 19 mM) were incubated at 37 °C.
17: $C_{76}H_{126}N_8O_{55}$ (2030.73), ESI-MS: $M_{calc.}$ = 2053.72 $(M+Na)^+$; M_{found} = 2053.92 $(M+Na)^+$,
18: $C_{82}H_{136}N_8O_{59}$ (2176.79), ESI-MS: $M_{calc.}$ = 2199.78 $(M+Na)^+$; M_{found} = 2200.00 $(M+Na)^+$.

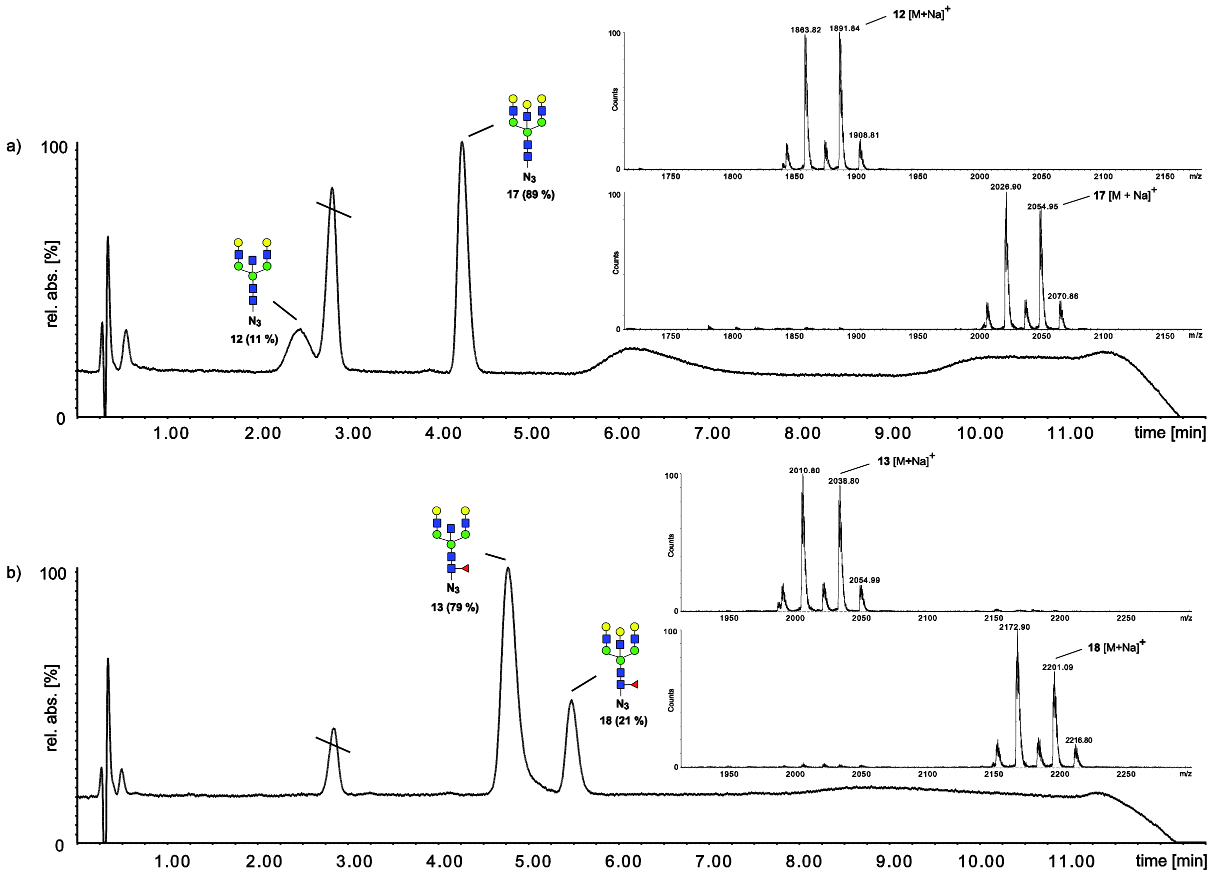

Scheme S4: *LC-MS monitoring (Hypercarb, 250 Å, 3 µm, 10 - 20 % acetonitrile/water + 0.1 % formic acid) of the galactosylation reaction of a) acceptor **12** and b) acceptor **13** after 3 d. Product ratios were determined by integration of the HPLC peaks.*

Preparative galactosylation of 7

18.4 mg (11.9 µmol, 1 eq) of octasaccharide azide **7** (final concentration 2 mM) were dissolved in 3.86 mL of water and 298 µL of sodium cacodylate buffer (500 mM, pH 7.4). Subsequently, 21.8 mg (35.7 µmol, 3 eq,) of UDP-Gal in 363 µL of water, 59.5 µL of $MnCl_2$ (100 mM), 9.2 µL of NaN_3 (1 M), 297 µL of bovine serum albumin (20 mg/mL) and 59.5 µL of alkaline phosphatase (1 U/µL) were added. The reaction was started by adding 36 µL of bovine GlcNAc β-1,4-galactosyltransferase (10 U/mL) and incubated at 30 °C. After 24 h the reaction was stopped by freezing at -20 °C. The reaction mixture was purified by size exclusion chromatography (Superdex 30, 25 x 780 mm, 0.1 M ammonium hydrogen carbonate, 2 mL/min) and lyophilized. The residue was purified by RP-HPLC (YMC Hydrosphere C18, 20 x 50 mm, 5 µm, 7 mL/min, 0-4 % acetonitrile/water with 0.1 % formic acid, retention time 21 min.). The fractions containing the decasaccharide azide **12** were

lyophilized (20.5 mg, 11 µmol, 92.4 %). $C_{70}H_{116}N_8O_{50}$ (1868.6780), HR-MS: $M_{calc.}$ = 1869.6853 $(M+H)^+$, 1891.6672 $(M+Na)^+$; M_{found} = 1869.6789 $(M+H)^+$, 1891.6592 $(M+Na)^+$;

^{1}H-NMR (500 MHz, D_2O): δ = 5.09 (d, $J_{1,2}$ < 1.0 Hz, 1H, H-1^4), 5.04 (d, $J_{1,2}$ < 1.0 Hz, 1H, H-1$^{4'}$), 4.80-4.76 (m, 1H, H-1^1), 4.72 (d, $J_{1,2}$ < 1.0 Hz, 1H, H-1^3), 4.66-4.58 (m, 3H, H-1^2, H-1^5, H-1$^{5'}$), 4.53-4.46 (m, 3H, H-1^G, H-1$^{G'}$, H-1^B), 4.30-4.28 (m, 1H, H-2^4), 4.22-4.19 (m, 1H, H-2^3), 4.18-4.16 (m, 1H, H-2$^{4'}$), 4.11 (dd, $J_{1,2}$ = $J_{2,3}$ = 9.8 Hz, 1H, H-4^3), 4.04-3.47 (m, 54H, H-6a^B, H-6a^5, H-6a$^{5'}$, H-6a^4, H-6a$^{4'}$, H-4^G, H-4$^{G'}$, H-6a^3, H-6b^3, H-3^4, H-6b^5, H-6b$^{5'}$, H-3^3, H-6a^2, H-6a^1, H-3$^{4'}$,H-2^2, H-6a^G, H-6a$^{G'}$, H-6b^G, H-6b$^{G'}$, H-4^2, H-2^5, H-2$^{5'}$, H-5^G, H-5$^{G'}$, H-6b^2, H-2^1, H-3^2, H-4^5, H-4$^{5'}$, H-3^5, H-3$^{5'}$, H-5^4, H-2^B, H-6b^B, H-6b^1, H-3^G, H-3$^{G'}$, H-4^1, H-3^1, H-5$^{4'}$, H-6b^4, H-6b$^{4'}$, H-5^5, H-5^1, H-5^2, H-3^B, H-5^3, H-2^G, H-2$^{G'}$, H-5$^{5'}$, H-4^4, H-4$^{4'}$), 3.46-3.40 (m, 1H, H-5^B), 3.29 (dd, $J_{3,4}$ = $J_{4,5}$ = 9.3 Hz, 1H, H-4^B), 2.12 (s, 3H, Ac), 2.10-2.06 (m, 11H, Ac);

^{13}C-NMR (125 MHz, D_2O): δ = 176.3, 176.2, 176.1, 176.1, 176.0 (C=O NAc), 104.5 (C-1^{G}β, $^1J_{C-1,H-1}$ = 164.1 Hz), 104.4 (C-1$^{G'}$β, $^1J_{C-1,H-1}$ = 164.1 Hz), 102.9 (C-1^{2}β, $^1J_{C-1,H-1}$ = 164.1 Hz), 102.0 (C-1^{B}β, $^1J_{C-1,H-1}$ = 161.5 Hz), 101.5 (C-1^{3}β, $^1J_{C-1,H-1}$ = 162.7 Hz), 101.4 (C-1^{4}α, $^1J_{C-1,H-1}$ = 172.0 Hz), 101.2 (C-1^{5}β, $^1J_{C-1,H-1}$ = 162.1 Hz), 100.8 (C-1$^{5'}$β, $^1J_{C-1,H-1}$ = 162.1 Hz), 99.2 (C-1$^{4'}$α, $^1J_{C-1,H-1}$ = 171.8 Hz), 90.0 (C-1^{1}β, $^1J_{C-1,H-1}$ = 159.8 Hz), 80.3 (C-3^3), 80.2 (C-4^1), 80.0 (C-4^2), 79.7 (C-4^5), 79.5 (C-4$^{5'}$), 78.2 (C-5^B), 77.9 (C-5^1), 77.9 (C-2^4), 77.7 (C-2$^{4'}$), 76.8 (C-5^G), 76.8 (C-5$^{G'}$), 76.2 (C-5^5), 76.1 (C-5^2), 76.0 (C-5$^{5'}$), 75.8 (C-5^3), 75.0 (C-5^4), 74.8 (C-5$^{4'}$), 74.8 (C-3^B), 74.0 (C-3^G), 73.9 (C-3$^{G'}$), 73.7 (C-3^1), 73.7 (C-3^5), 73.5 (C-3$^{5'}$), 73.1 (C-3^2), 72.8 (C-4^3), 72.6 (C-4^B), 72.4 (C-2^G), 72.4 (C-2$^{G'}$), 71.8 (C-2^3), 70.9 (C-3$^{4'}$), 70.8 (C-3^4), 70.0 (C-4^G), 70.0 (C-4$^{G'}$), 69.0 (C-4^4), 68.8 (C-4$^{4'}$), 66.7 (C-6^3), 63.4 (C-6^4), 63.3 (C-6$^{4'}$), 63.1 (C-6^B), 62.5 (C-6^G), 62.5 (C-6$^{G'}$), 61.4 (C-6^5), 61.4 (C-6$^{5'}$), 61.3 (C-6^1), 61.3 (C-6^2), 57.6 (C-2^B), 56.7 (C-2^2), 56.3 (C-2^5), 56.2 (C-2$^{5'}$), 56.0 (C-2^1), 23.9, 23.8, 23.7, 23.6, 23.6 (NAc).

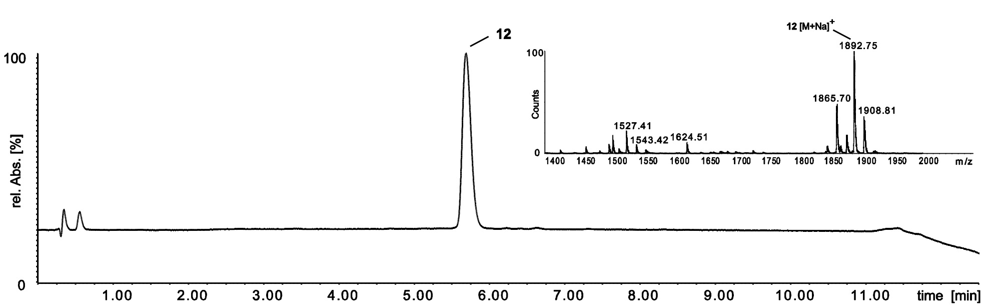

***Scheme S4**: LC-MS of purified digalactosylated **12**.*

Enzymatic galactosylation of 12

7.0 mg (3.74 µmol, 1 eq) of decasaccharide azide **12** (final concentration 19 mM) were dissolved in 64 µL of Tris hydrochloride buffer (50 mM, pH 7.5). Subsequently, 9.80 mg (15.71 µmol, 4.2 eq,) of UDP-Gal in 64 µL of Tris hydrochloride buffer (50 mM, pH 7.5), 9.82 µL of bovine serum albumin (20 mg/mL), 2.18 µL of $MnCl_2$ (100 mM), 1.09 µL of NaN_3 (100 mM) and 2.18 µL of alkaline phosphatase (1 U/µL) were added. The reaction was started by adding 54 µL of bovine GlcNAc β-1,4-galactosyltransferase (10 U/mL) and incubated for 90 h at 37 °C. After 90 h, 9.80 mg (15.71 µmol, 4.2 eq,) of UDP-Gal in 5 µL of Tris hydrochloride buffer (50 mM, pH 7.5), 54 µL of bovine GlcNAc β-1,4-galactosyltransferase (10 U/mL) and 0.49 µL of alkaline phosphatase (1 U/µL) were added. The reaction mixture was incubated for 2 d at 37 °C and subsequently purified by size exclusion chromatography (Superdex 30, 16 x 600 mm, 0.1 M ammonium hydrogen carbonate, 1 mL/min, retention time 82 min) and lyophilized. The residue was purified by RP-HPLC (YMC Hydrosphere C18, 10 x 150 mm, 5 µm, 2.5 mL/min, 0-3 % acetonitrile/water with 0.1 % formic acid, retention time 81 min.). The fractions containing the undecasaccharide azide **17** were lyophilized (6.36 mg, 3.40 µmol, 91 %). R_f (digalactoside **12**) = 0.53 (*i*-propanol/1 M ammonium acetate 2:1), R_f (trigalactoside **17**) = 0.43 (*i*-propanol/1 M ammonium acetate 2:1), $C_{76}H_{126}N_8O_{55}$ (2030.7309), HR-MS: $M_{calc.}$ = 2031.7382 $(M+H)^+$, 2053.7201 $(M+Na)^+$; M_{found} = 2031.7447 $(M+H)^+$, 2053.7174 $(M+Na)^+$;

^{1}H-NMR (500 MHz, D_2O): δ = 4.93 (d, $J_{1,2}$ < 1 Hz, 1H, H-1^4), 4.91 (d, $J_{1,2}$ < 1 Hz, 1H, H-$1^{4'}$), 4.64 (m, 1H, H-1^1β), 4.57 (d, $J_{1,2}$ < 1 Hz, 1H, H-1^3), 4.50 (d, $J_{1,2}$ < 1 Hz, 1H, H-1^5), 4.49-4.47 (m, 2H, H-1^2, H-1^5), 4.36-4.31 (m, 4H, H-1^B, H-1^G, H-1^{GB}, H-$1^{G'}$), 4.16 (m, 1H, H-2^4), 4.07 (m, 1H, H-2^3), 4.02 (m, 1H, H-$2^{4'}$), 3.98-3.94 (m, 2H, H-4^3, H-$6a^B$), 3.88-3.34 (m, 61H, H-$6a^5$, H-$6a^{5'}$, H-$6a^4$, H-$6a^{4'}$, H-4^G, H-4^{GB}, H-$4^{G'}$, H-3^4, H-$6a^3$, H-$6b^3$, H-$6b^5$, H-3^3, H-$6a^2$, H-$6b^{5'}$, H-$3^{4'}$, H-$6a^1$, H-2^2, H-4^2, H-4^5, H-$4^{5'}$, H-2^1, H-3^1, H-3^2, H-2^5, H-$6b^B$, H-$6a^G$, H-$6b^G$, H-$6a^{G'}$, H-$6b^{G'}$, H-$6a^{GB}$, H-$6b^{GB}$, H-3^5, H-$6b^1$, H-2^B, H-3^B, H-$2^{5'}$, H-$3^{5'}$, H-5^G, H-5^{GB}, H-$5^{G'}$, H-$6b^2$, H-5^4, H-3^G, H-3^{GB}, H-$3^{G'}$, H-4^1, H-5^5, H-$5^{4'}$, H-5^1, H-5^3, H-5^B, H-$6b^4$, H-$6b^{4'}$, H-5^2, H-$5^{5'}$, H-2^G, H-2^{GB}, H-$2^{G'}$, H-4^B, H-4^4, H-$4^{4'}$), 1.97, 1.95, 1.93, 1.92 (4 s, 15H, NAc)

^{13}C-NMR (125 MHz, D_2O): δ = 174.7, 174.6, 174.6, 174.5, 174.5 (C=O, NAc), 102.9 (C-1^Gβ, $J_{C\text{-}1,H\text{-}1}$ = 165.6 Hz), 102.9 (C-1^{GB}β, $J_{C\text{-}1,H\text{-}1}$ = 165.6 Hz), 102.9 (C-$1^{G'}$β, $J_{C\text{-}1,H\text{-}1}$ = 165.6 Hz),

101.3 (C-1^{2}β, $J_{C-1,H-1}$ = 159.9 Hz), 100.4 (C-1^{B}β, $J_{C-1,H-1}$ = 162.4 Hz), 100.0 (C-1^{3}β, $J_{C-1,H-1}$ = 162.3 Hz), 99.9 (C-1^{4}α, $J_{C-1,H-1}$ = 170.7 Hz), 99.5 (C-1^{5}β, $J_{C-1,H-1}$ = 159.7 Hz), 99.2 (C-1$^{5'}$β, $J_{C-1,H-1}$ = 159.1 Hz), 97.6 (C-1$^{4'}$α, $J_{C-1,H-1}$ = 170.7 Hz), 88.4 (C-1^{1}β, $J_{C-1,H-1}$ = 163.0 Hz), 80.2 (C-4^{B}), 79.0 (C-3^{3}), 78.6 (C-4^{1}), 78.3 (C-4^{2}), 78.3 (C-4^{5}), 78.3 (C-4$^{5'}$), 76.3 (C-5^{1}), 76.3 (C-2^{4}), 76.1 (C-2$^{4'}$), 75.6 (C-5^{B}), 75.3 (C-5^{G}), 75.3 (C-5^{GB}), 75.3 (C-5$^{G'}$), 74.6 (C-5^{3}), 74.6 (C-5^{5}), 74.4 (C-5$^{5'}$), 74.4 (C-5^{2}), 73.5 (C-5^{4}), 73.3 (C-5$^{4'}$), 72.3 (C-3^{G}), 72.3 (C-3^{GB}), 72.3 (C-3$^{G'}$), 72.0 (C-3^{1}), 72.0 (C-3^{2}), 72.0 (C-3^{5}), 72.0 (C-3^{B}), 72.0 (C-3$^{5'}$), 71.4 (C-4^{3}), 70.9 (C-2^{G}), 70.9 (C-2^{GB}), 70.9 (C-2$^{G'}$), 70.3 (C-2^{3}), 69.4 (C-3$^{4'}$), 69.3 (C-3^{4}), 68.4 (C-4^{G}), 68.4 (C-4^{GB}), 68.4 (C-4$^{G'}$), 68.3 (C-6^{1}), 68.3 (C-6^{2}), 67.4 (C-4^{4}), 67.4 (C-4$^{4'}$), 65.1 (C-6^{3}), 61.8 (C-6^{4}), 61.8 (C-6$^{4'}$), 60.9 (C-6^{B}), 60.9 (C-6^{G}), 60.8 (C-6$^{G'}$), 60.1 (C-6^{GB}), 59.8 (C-6^{5}), 59.7 (C-6$^{5'}$), 55.2 (C-2^{2}), 54.7 (C-2^{1}β), 54.7 (C-2^{5}), 54.7 (C-2^{B}), 54.7 (C-2$^{5'}$), 23.1, 22.3, 22.3, 22.3, 22.0 (NAc).

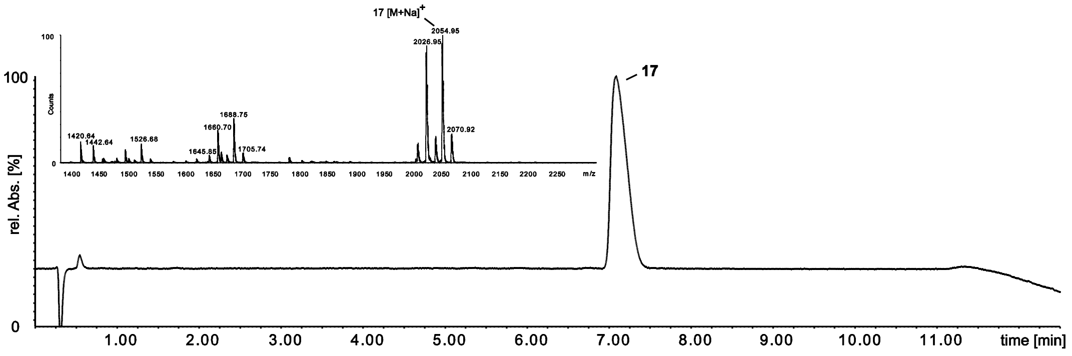

***Scheme S5**: LC-MS of purified trigalactosylated **17**.*

Synthesis of 20

A solution of undecasaccharide azide **17** (5.44 mg, 2.68 µmol) in 535 µL of dry MeOH was treated with DIPEA (13.6 µL, 80.32 µmol) and 1,3-propanedithiol (48.4 µL, 481.86 µmol) under an argon atmosphere. After 3 h the glycosylamine was dried under high vacuum. The dried residue was precipitated by addition of diethylether (4 mL). After centrifugation (5300 rpm, 5 min) and removal of the supernatant the procedure was repeated twice. The final precipitate was dried for 2 h under high vacuum. The residue was dissolved in 141.7 µL of DMF/DMSO (1:1) and a solution of compound **18** (3.97 mg, 8.03 µmol), HATU (3.05 mg, 8.03 µmol), HOAt (1.09 mg, 8.03 µmol) and DIPEA (4.4 µL, 26.78 µmol) in 126 µL of DMF/DMSO (1:1) was added. After 5 min DIPEA (3.2 µL, 19.48 µmol) was added. After 17.5 h the mixture was dried under high vacuum for 3 h. The dried residue was precipitated by addition of diethylether (4 mL). After centrifugation (5300 rpm, 5 min) and removal of the

supernatant the procedure was repeated twice. The final precipitate was dried for 2 h under high vacuum. The dried residue was purified by RP-HPLC (YMC Hydrosphere C18, 10 x 150 mm, 5 µm, 2.5 mL/min, 25-45 % acetonitrile/water with 0.1 % formic acid, retention time 32 min.) to furnish the protected conjugate **19** (5.66 mg, 2.28 µmol, 85 %). After lyophilization the protected conjugate **19** (3.6 mg, 1.45 µmol) was dissolved in water (619 µL) and 1 M NaOH (206 µL) was added dropwise. After 2 h the mixture was neutralized with 1 M HCl and lyophilized. The dried residue was purified by RP-HPLC (YMC Hydrosphere C18, 10 x 150 mm, 5 µm, 2.5 mL/min, 0-15 % acetonitrile/water with 0.1 % formic acid, retention time 45 min.) to furnish the deprotected conjugate **20** (3.27 mg, 1.38 µmol, 95 %).

R_f (glycosylamine) = 0.14 (*i*-propanol/1 M ammonium acetate 2:1), R_f (protected conjugate **19**) = 0.71 (*i*-propanol/1 M ammonium acetate 1.5:1), R_f (deprotected conjugate **20**) = 0.19 (*i*-propanol/1 M ammonium acetate 1.5:1), $C_{91}H_{142}N_8O_{64}$ (2370.8103), HR-MS: $M_{calc.}$ = 2371.8176 $(M+H)^+$, 2393.7995 $(M+Na)^+$; M_{found} = 2371.8140 $(M+H)^+$, 2393.7934 $(M+Na)^+$;

20: ^{1}H-NMR (500 MHz, D_2O): δ = 7.38 (s, 1H, H-Ar), 7.27 (d, $J_{1,2}$ = 8.4 Hz, 1H, H-Ar), 7.04 (d, $J_{1,2}$ = 8.6 Hz, 1H, H-Ar), 5.09 (d, $J_{1,2}$ = 9.4 Hz, 1H, H-1^1β), 4.94 (d, $J_{1,2}$ < 1 Hz, 1H, H-1^4), 4.91 (d, $J_{1,2}$ < 1 Hz, 1H, H-$1^{4'}$), 4.58 (d, $J_{1,2}$ < 1 Hz, 1H, H-1^3), 4.53-4.49 (m, 3H, H-1^2, H-1^5, H-$1^{5'}$), 4.37-4.34 (m, 4H, H-1^B, H-1^G, H-1^{GB}, H-$1^{G'}$), 4.29-4.28 (m, 2H, CH_2, CH_2), 4.25-4.23 (m, 2H, CH_2, CH_2), 4.16 (m, 1H, H-2^4), 4.07 (m, 1H, H-2^3), 4.03 (m, 1H, H-$2^{4'}$), 3.96 (m, 2H, H-4^3, $6a^B$), 3.90-3.36 (m, 65H, H-2^1, H-$6a^5$, H-$6a^{5'}$, H-$6a^4$, H-$6a^{4'}$, CH_2, CH_2, H-4^G, H-4^{GB}, H-$4^{G'}$, H-$6a^3$, H-$6b^3$, H-$6b^5$, H-3^4, H-3^3, H-3^1, H-2^2, H-$6a^2$, H-$6b^{5'}$, H-$3^{4'}$, H-3^2, H-2^5, H-$6b^B$, H-$6a^G$, H-$6b^G$, H-$6a^{G'}$, H-$6b^{G'}$, H-$6a^{GB}$, H-$6b^{GB}$, H-3^5, H-$6b^1$, H-3^B, H-$2^{5'}$, H-$3^{5'}$, H-4^1, H-$6a^1$, H-4^2, H-4^5, H-2^B, H-$4^{5'}$, H-5^G, H-5^{GB}, H-$5^{G'}$, H-$6b^2$, H-5^4, H-3^G, H-3^{GB}, H-$3^{G'}$, H-5^1, H-5^2, H-$5^{4'}$, H-5^5, CH_2, CH_2, H-5^B, H-2^G, H-2^{GB}, H-$2^{G'}$, H-5^3, H-$6b^4$, H-$6b^{4'}$, H- $5^{5'}$, H-4^B, H-4^4, H-$4^{4'}$), 1.98, 1.95, 1.94, 1.87 (4s, 15H, NAc)

^{13}C-NMR (125 MHz, D_2O): δ = 175.1, 174.9, 174.8, 174.6, 174.5 (C=O, NAc), 122.7, 120.6, 120.3 (C-Ar), 103.0 (C-1^Gβ, $J_{C\text{-}1,H\text{-}1}$ = 161.4 Hz), 103.0 (C-1^{GB}β, $J_{C\text{-}1,H\text{-}1}$ = 161.4 Hz), 103.0 (C-$1^{G'}$β, $J_{C\text{-}1,H\text{-}1}$ = 161.4 Hz), 101.2 (C-1^2β, $J_{C\text{-}1,H\text{-}1}$ = 164.0 Hz), 100.5 (C-1^Bβ, $J_{C\text{-}1,H\text{-}1}$ = 160.3 Hz), 100.0 (C-1^4α, $J_{C\text{-}1,H\text{-}1}$ = 170.3 Hz), 100.0 (C-1^3β, $J_{C\text{-}1,H\text{-}1}$ = 161.0 Hz), 99.6 (C-1^5β, $J_{C\text{-}1,H\text{-}1}$ = 156.7 Hz), 99.6 (C-$1^{5'}$β, $J_{C\text{-}1,H\text{-}1}$ = 159.7 Hz), 97.6 (C-$1^{4'}$α, $J_{C\text{-}1,H\text{-}1}$ = 171.2 Hz), 80.3 (C-4^B), 79.2 (C-1^1β, $J_{C\text{-}1,H\text{-}1}$ = 156.7 Hz), 79.0 (C-3^3), 78.4 (C-4^1), 78.4 (C-4^2), 78.4 (C-4^5), 78.4 (C-$4^{5'}$), 76.2 (C-5^1), 76.2 (C-2^4), 76.1 (C-$2^{4'}$), 75.7 (C-5^B), 75.2 (C-5^G), 75.2 (C-5^{GB}), 75.2 (C-$5^{G'}$), 74.5 (C-5^2), 74.5 (C-5^5), 74.4 (C-5^3), 74.4 (C-$5^{5'}$), 73.6 (C-5^4), 73.3 (C-$5^{4'}$), 72.3 (C-3^G),

72.3 (C-3^{GB}), 72.3 (C-3$^{G'}$), 72.2 (C-3^{1}), 72.1 (C-3^{2}), 72.1 (C-3^{5}), 72.1 (C-3^{B}), 72.1 (C-3$^{5'}$), 71.2 (C-4^{3}), 70.8 (C-2^{G}), 70.8 (C-2^{GB}), 70.8 (C-2$^{G'}$), 70.3 (C-2^{3}), 69.5 (C-3$^{4'}$), 69.2 (C-3^{4}), 68.5 (C-4^{G}), 68.5 (C-4^{GB}), 68.5 (C-4$^{G'}$), 68.3 (C-6^{1}), 68.3 (C-6^{2}), 67.3 (C-4^{4}), 67.3 (C-4$^{4'}$), 65.1 (C-6^{3}), 61.8 (C-6^{4}), 61.8 (C-6$^{4'}$), 60.7 (C-6^{B}), 60.7 (C-6^{G}), 60.6 (C-6$^{G'}$), 60.1 (C-6^{GB}), 59.8 (C-6^{5}), 59.7 (C-6$^{5'}$), 55.5 (C-2^{B}), 55.2 (C-2^{2}), 54.8 (C-2^{5}), 54.8 (C-2$^{5'}$), 54.0 (C-2^{1}), 22.4, 22.3, 22.2, 22.1, 21.9 (NAc).

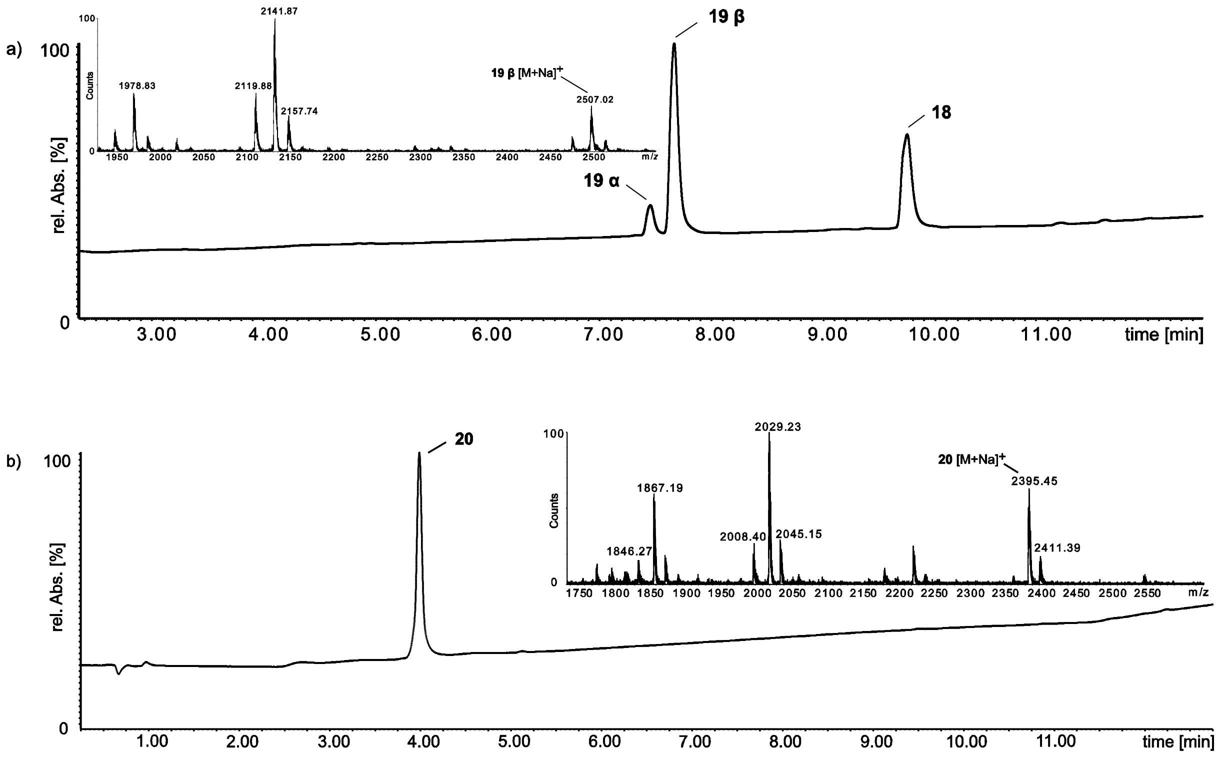

***Scheme S6**: LC-MS of a) crude conjugate **19** and b) purified deprotected conjugate **20**.*

Complexation of 20

0.5 mg of **20** (0.21 µmol, 1 eq., final concentration 0.4 mM) were dissolved in 450 µL of D_2O and 50 µL of 500 mM D_2O-imidazole buffer (pH 7.0). Either 8.3 µL (0.21 µmol, 1 eq.) of lanthanum trichloride heptahydrate in D_2O (9.4 mg/mL), or 8.3 µL (0.21 µmol, 1 eq.) of dysprosium trichloride hexahydrate in D_2O (9.5 mg/mL) were added to prepare the complexes **20a** and **20b**.

20a: ^{1}H-NMR (500 MHz, D_2O): δ = 7.81 (s, 1H, H-Ar), 7.61 (d, $J_{1,2}$ = 9.0 Hz, 1H, H-Ar), 7.60 (d, $J_{1,2}$ = 9.6 Hz, 1H, H-Ar), 5.14 (d, $J_{1,2}$ = 9.5 Hz, 1H, H-1^{1}β), 4.94 (d, $J_{1,2}$ < 1 Hz, 1H, H-1^{4}), 4.91 (d, $J_{1,2}$ < 1 Hz, 1H, H-1$^{4'}$), 4.58 (d, $J_{1,2}$ < 1 Hz, 1H, H-1^{3}), 4.53-4.49 (m, 3H, H-1^{2},

H-1^{5}, H-1$^{5'}$), 4.37-4.34 (m, 4H, H-1^{B}, H-1^{G}, H-1^{GB}, H-1$^{G'}$), 4.16 (m, 1H, H-2^{4}), 4.07 (m, 1H, H-2^{3}), 4.03 (m, 1H, H-2$^{4'}$), 3.96 (m, 2H, H-4^{3}, H-6a^{B}), 3.90-3.36 (m, 65H, H-2^{1}, H-6a^{5}, H-6a$^{5'}$, H-6a^{4}, H-6a$^{4'}$, CH_2, CH_2, H-4^{G}, H-4^{GB}, H-4$^{G'}$, H-6a^{3}, H-6b^{3}, H-6b^{5}, H-3^{4}, H-3^{3}, H-3^{1}, H-2^{2}, H-6a^{2}, H-6b$^{5'}$, H-3$^{4'}$, H-3^{2}, H-2^{5}, H-6b^{B}, H-6a^{G}, H-6b^{G}, H-6a$^{G'}$, H-6b$^{G'}$, H-6a^{GB}, H-6b^{GB}, H-3^{5}, H-6b^{1}, H-3^{B}, H-2$^{5'}$, H-3$^{5'}$, H-4^{1}, H-6a^{1}, H-4^{2}, H-4^{5}, H-2^{B}, H-4$^{5'}$, H-5^{G}, H-5^{GB}, H-5$^{G'}$, H-6b^{2}, H-5^{4}, H-3^{G}, H-3^{GB}, H-3$^{G'}$, H-5^{1}, H-5^{2}, H-5$^{4'}$, H-5^{5}, CH_2, CH_2, H-5^{B}, H-2^{G}, H-2^{GB}, H-2$^{G'}$, H-5^{3}, H-6b^{4}, H-6b$^{4'}$, H-5$^{5'}$, H-4^{B}, H-4^{4}, H-4$^{4'}$), 1.98, 1.94, 1.87 (5s, 15H, NAc)

^{13}C-NMR (125 MHz, D_2O): δ = 175.1, 174.9, 174.8, 174.6, 174.5 (C=O, NAc), 128.2, 126.9, 125.8 (C-Ar), 103.0 (C-1^{G}β, $J_{C\text{-}1,H\text{-}1}$ = 161.4 Hz), 103.0 (C-1^{GB}β, $J_{C\text{-}1,H\text{-}1}$ = 161.4 Hz), 103.0 (C-1$^{G'}$β, $J_{C\text{-}1,H\text{-}1}$ = 161.4 Hz), 101.2 (C-1^{2}β, $J_{C\text{-}1,H\text{-}1}$ = 164.0 Hz), 100.5 (C-1^{B}β, $J_{C\text{-}1,H\text{-}1}$ = 160.3 Hz), 100.0 (C-1^{4}α, $J_{C\text{-}1,H\text{-}1}$ = 170.3 Hz), 100.0 (C-1^{3}β, $J_{C\text{-}1,H\text{-}1}$ = 161.0 Hz), 99.6 (C-1^{5}β, $J_{C\text{-}1,H\text{-}1}$ = 156.7 Hz), 99.6 (C-1$^{5'}$β, $J_{C\text{-}1,H\text{-}1}$ = 159.7 Hz), 97.6 (C-1$^{4'}$α, $J_{C\text{-}1,H\text{-}1}$ = 171.2 Hz), 80.3 (C-4^{B}), 79.2 (C-1^{1}β, $J_{C\text{-}1,H\text{-}1}$ = 156.7 Hz), 79.0 (C-3^{3}), 78.4 (C-4^{1}), 78.4 (C-4^{2}), 78.4 (C-4^{5}), 78.4 (C-4$^{5'}$), 76.2 (C-5^{1}), 76.2 (C-2^{4}), 76.1 (C-2$^{4'}$), 75.7 (C-5^{B}), 75.2 (C-5^{G}), 75.2 (C-5^{GB}), 75.2 (C-5$^{G'}$), 74.5 (C-5^{2}), 74.5 (C-5^{5}), 74.4 (C-5^{3}), 74.4 (C-5$^{5'}$), 73.6 (C-5^{4}), 73.3 (C-5$^{4'}$), 72.3 (C-3^{G}), 72.3 (C-3^{GB}), 72.3 (C-3$^{G'}$), 72.2 (C-3^{1}), 72.1 (C-3^{2}), 72.1 (C-3^{5}), 72.1 (C-3^{B}), 72.1 (C-3$^{5'}$), 71.2 (C-4^{3}), 70.8 (C-2^{G}), 70.8 (C-2^{GB}), 70.8 (C-2$^{G'}$), 70.3 (C-2^{3}), 69.5 (C-3$^{4'}$), 69.2 (C-3^{4}), 68.5 (C-4^{G}), 68.5 (C-4^{GB}), 68.5 (C-4$^{G'}$), 68.3 (C-6^{1}), 68.3 (C-6^{2}), 67.3 (C-4^{4}), 67.3 (C-4$^{4'}$), 65.1 (C-6^{3}), 61.8 (C-6^{4}), 61.8 (C-6$^{4'}$), 60.7 (C-6^{B}), 60.7 (C-6^{G}), 60.6 (C-6$^{G'}$), 60.1 (C-6^{GB}), 59.8 (C-6^{5}), 59.7 (C-6$^{5'}$), 55.5 (C-2^{B}), 55.2 (C-2^{2}), 54.8 (C-2^{5}), 54.8 (C-2$^{5'}$), 54.0 (C-2^{1}), 22.4, 22.3, 22.2, 22.1, 21.9 (NAc).

20b: ^{1}H-NMR (500 MHz, D_2O): δ = 4.70 (d, $J_{1,2} < 1$ Hz, 1H, H-1^{4}), 4.45 (d, $J_{1,2} < 1$ Hz, 1H, H-1$^{4'}$), 4.34 (d, $J_{1,2}$ = 7.0 Hz, 1H, H-1^{5}), 4.27 (d, $J_{1,2}$ = 7.8 Hz, 1H, H-1^{G}), 4.19 (d, $J_{1,2}$ = 7.8 Hz, 1H, H-1^{GB}), 4.09-3.98 (m, 5H, H-1^{B}, H-1$^{G'}$, H-1$^{5'}$, H-1^{3}, H-2^{4}), 3.80-3.75 (m, 4H, H-6a^{B}, H-6b^{B}, H-4^{G}, H-4^{GB}), 3.65-3.23 (m, 29H, H-2^{3}, H-4^{3}, H-3^{4}, H-2$^{4'}$, H-4$^{G'}$, H-5^{G}, H-2^{5}, H-3^{5}, H-4^{5}, H-5^{GB}, H-3^{G}, H-3^{GB}, H-3^{B}, H-2^{B}, H-5^{5}, H-3^{3}, H-6a^{3}, H-5$^{G'}$, H-5^{4}, H-6b^{3}, H-3$^{4'}$, H-2^{GB}, H-3$^{G'}$, H-2^{G}, H-4$^{5'}$, H-4^{4}, H-2$^{5'}$, H-4^{B}, H-5^{B}), 3.15 (m, 1H, H-5$^{4'}$), 3.02-2.95 (m, 5H, H-4$^{4'}$, H-3$^{5'}$, H-2$^{G'}$, H-5$^{5'}$, H-5^{3}) 2.74 (m, 2H, H-1^{2}, H-4^{2}), 2.64 (m, 1H, H-3^{2}), 2.39-2.36 (m, 2H, H-5^{2}, H-2^{2}), 1.80, 1.67, 1.46, 0.76 (4s, 12H, NAc)

^{13}C-NMR (125 MHz, D_2O): δ = 103.0 (C-1^{GB}β, $J_{C\text{-}1,H\text{-}1}$ = 161.2 Hz), 102.9 (C-1^{G}β, $J_{C\text{-}1,H\text{-}1}$ = 161.1 Hz), 102.5 (C-1$^{G'}$β, $J_{C\text{-}1,H\text{-}1}$ = 161.4 Hz), 100.3 (C-1^{B}β, $J_{C\text{-}1,H\text{-}1}$ = 162.8 Hz), 99.8 (C-

$1^{4}\alpha$, $J_{C\text{-}1,H\text{-}1}$ = 170.9 Hz), 99.5 (C-$1^{2}\beta$, $J_{C\text{-}1,H\text{-}1}$ = 164.0 Hz), 99.5 (C-$1^{5}\beta$, $J_{C\text{-}1,H\text{-}1}$ = 162.4 Hz), 99.4 (C-$1^{3}\beta$, $J_{C\text{-}1,H\text{-}1}$ = 162.5 Hz), 98.9 (C-$1^{5'}\beta$, $J_{C\text{-}1,H\text{-}1}$ = 161.4 Hz), 97.3 (C-$1^{4'}\alpha$, $J_{C\text{-}1,H\text{-}1}$ = 170.6 Hz), 80.0 (C-4^{B}), 78.6 (C-3^{3}), 78.3 (C-4^{5}), 78.1 (C-$4^{5'}$), 77.3 (C-4^{2}), 76.1 (C-2^{4}), 75.7 (C-$2^{4'}$), 75.5 (C-5^{B}), 75.2 (C-5^{G}), 75.2 (C-5^{GB}), 75.1 (C-4^{1}), 74.9 (C-$5^{G'}$), 74.5 (C-5^{5}), 74.2 (C-$5^{5'}$), 73.9 (C-5^{3}), 73.4 (C-5^{2}), 73.3 (C-5^{4}), 72.9 (C-$5^{4'}$), 72.2 (C-3^{G}), 72.2 (C-3^{GB}) 72.2 (C-$3^{G'}$), 72.1 (C-3^{1}), 71.8 (C-$3^{5'}$), 71.8 (C-3^{B}), 71.6 (C-3^{5}), 71.0 (C-3^{2}), 70.8 (C-4^{3}), 70.7 (C-2^{G}), 70.7 (C-2^{GB}), 70.5 (C-$2^{G'}$), 69.7 (C-2^{3}), 69.2 (C-3^{4}), 69.1 (C-$3^{4'}$), 68.6 (C-5^{1}), 68.4 (C-4^{G}), 68.3 (C-4^{GB}), 68.1 (C-$4^{G'}$), 67.4 (C-4^{4}), 66.9 (C-$4^{4'}$), 64.6 (C-6^{3}), 60.6 (C-6^{B}), 55.2 (C-2^{B}), 54.7 (C-2^{5}), 54.2 (C-$2^{5'}$), 53.8 (C-2^{2}), 22.1, 21.9, 21.3, 20.8 (NAc).

1H NMR, 500 MHz, D2O

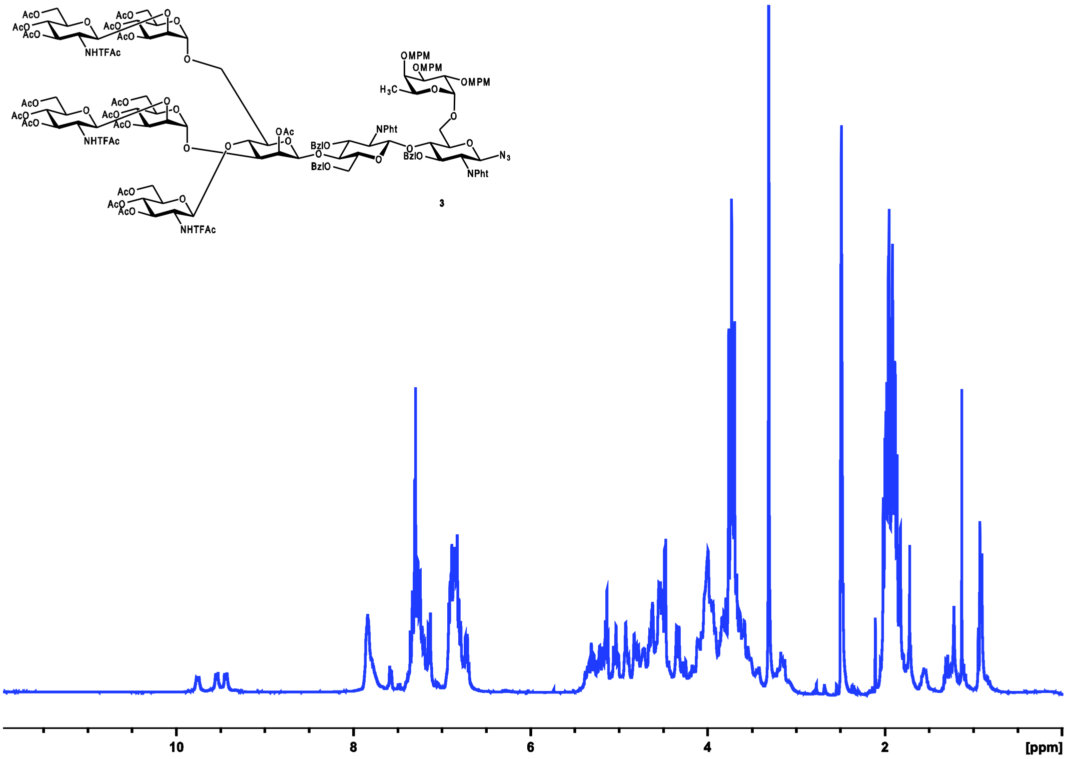

13C NMR, jmod, 125 MHz, D2O

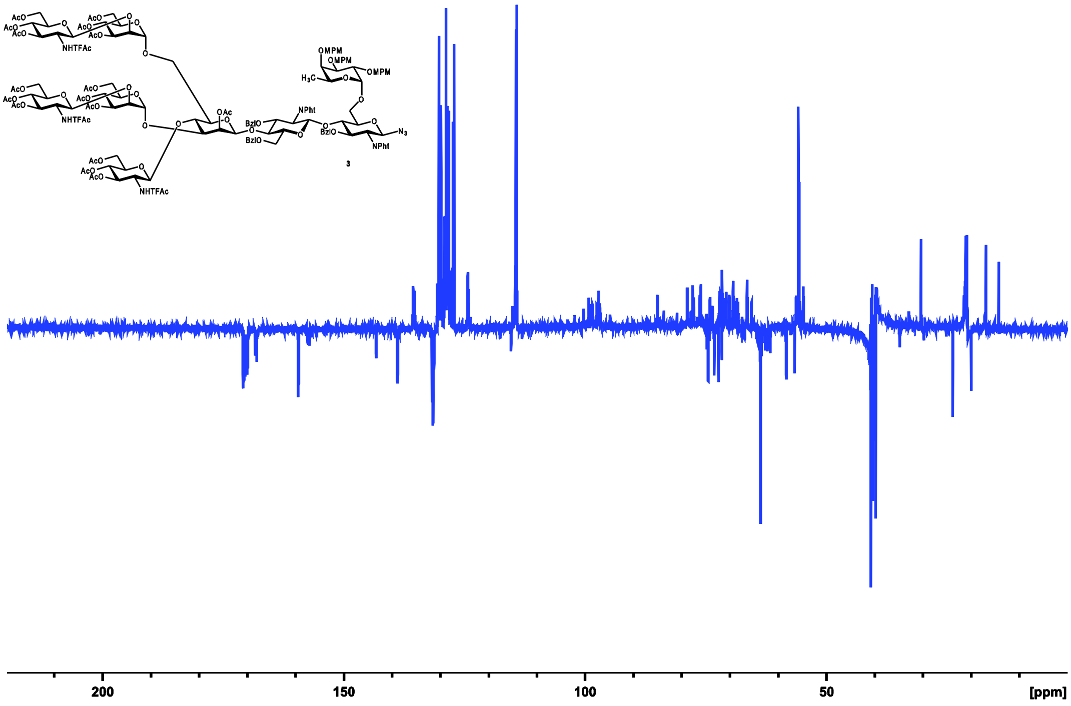

1H NMR, 500 MHz, D2O

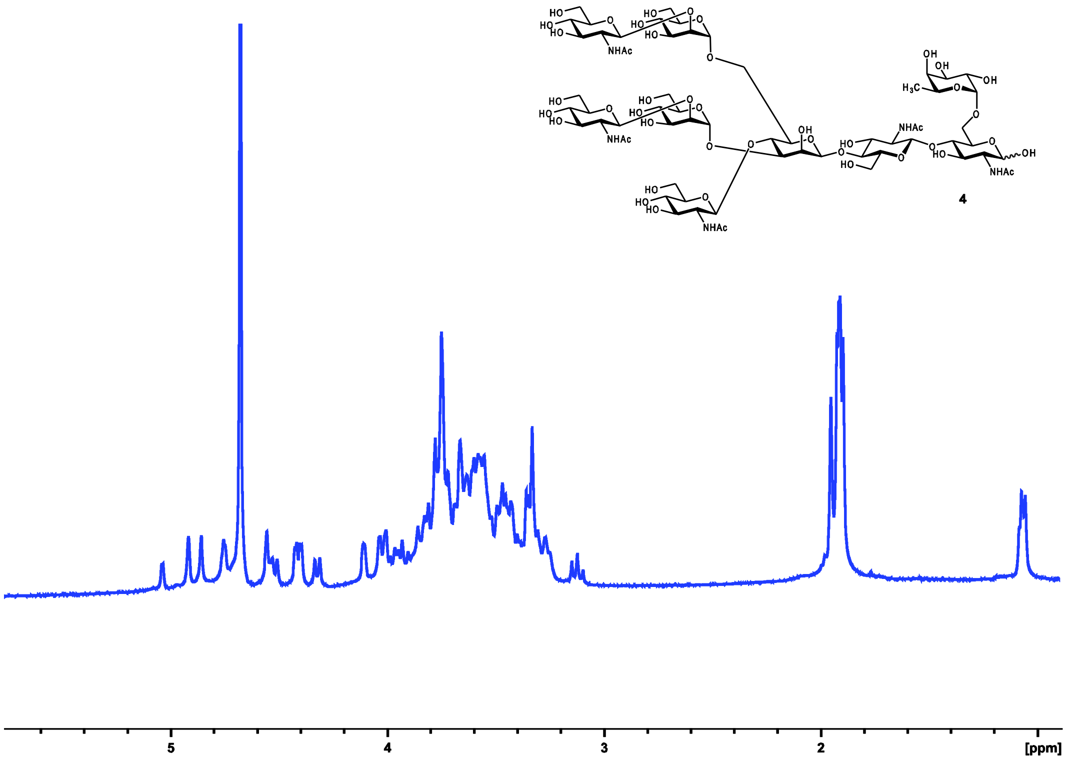

13C NMR, jmod, 125 MHz, D2O

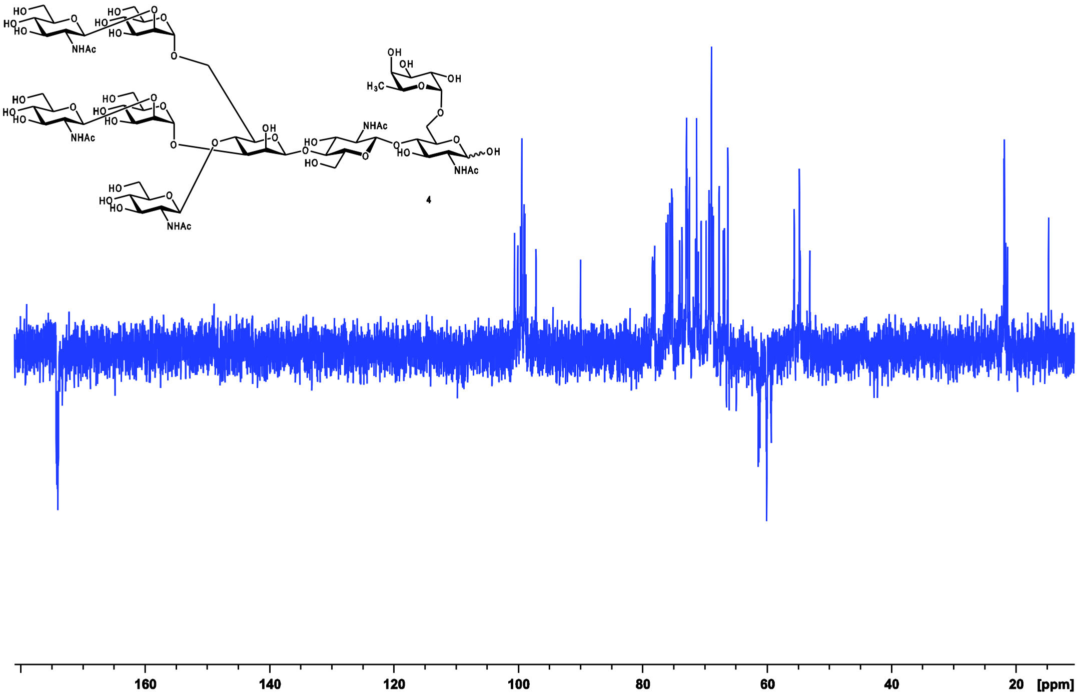

1H NMR, 500 MHz, D2O

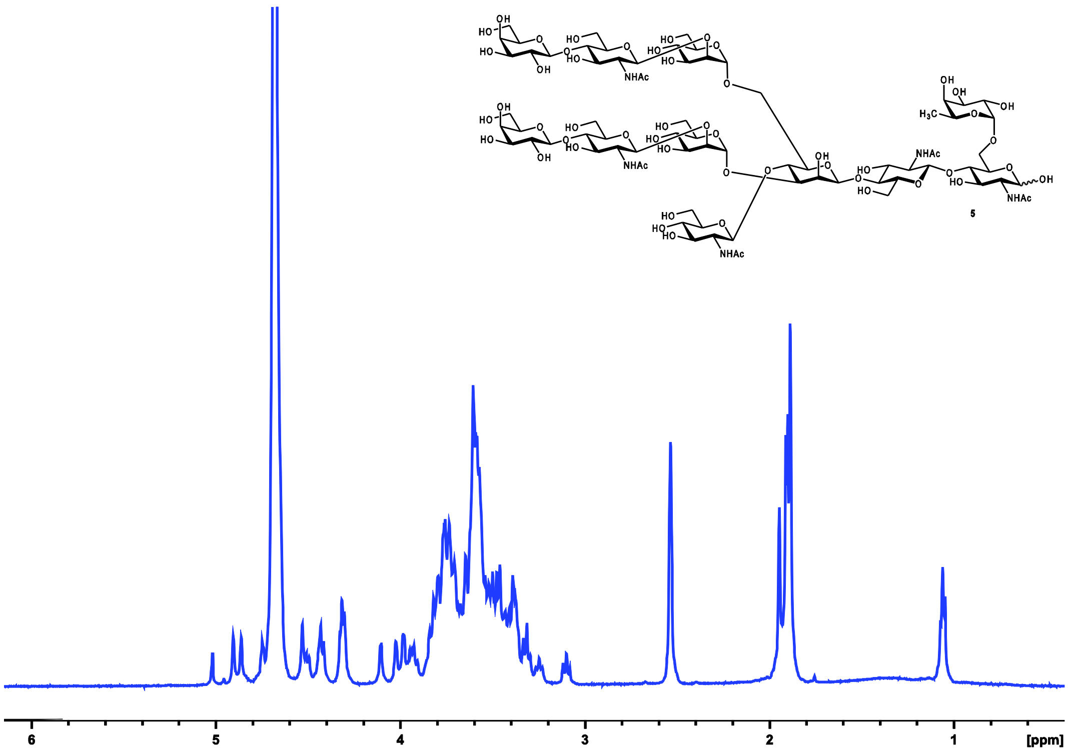

13C NMR, jmod, 125 MHz, D2O

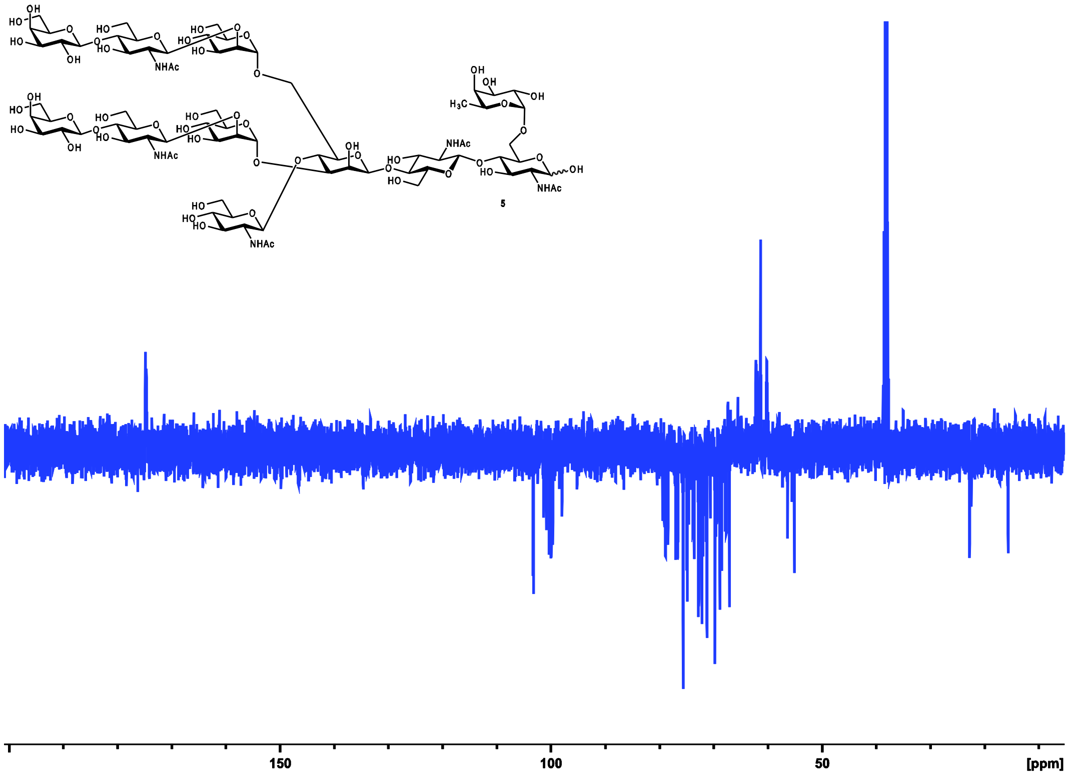

1H NMR, 500 MHz, D2O

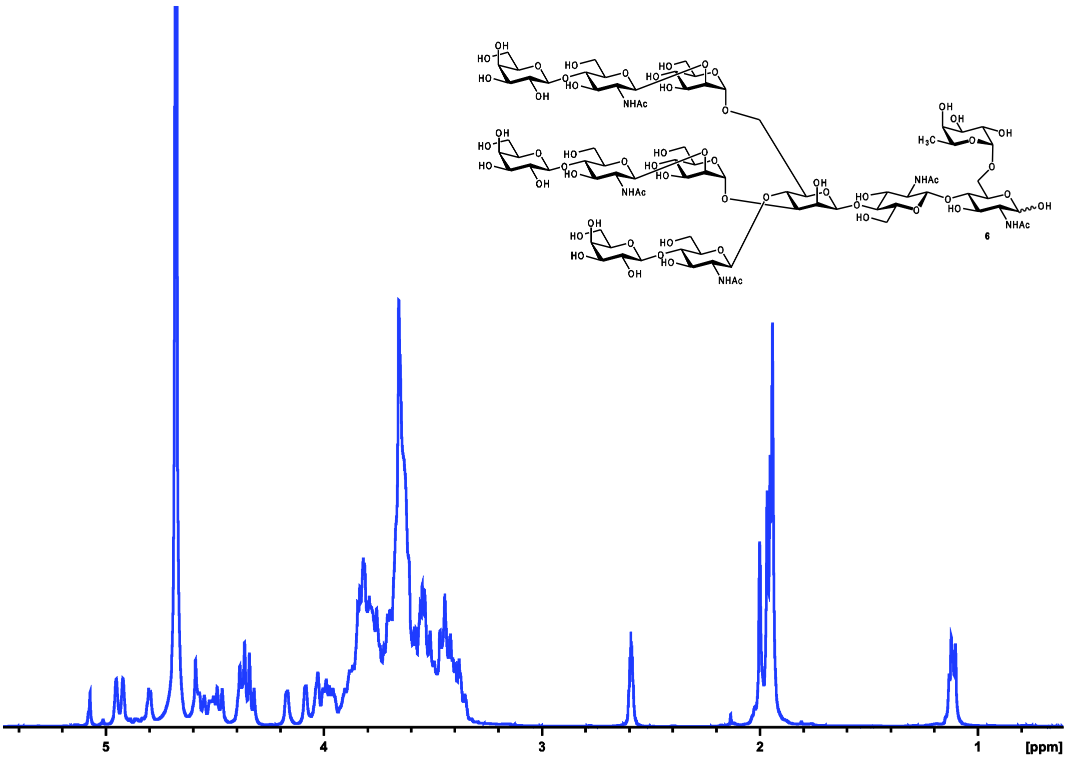

13C NMR, jmod, 125 MHz, D2O

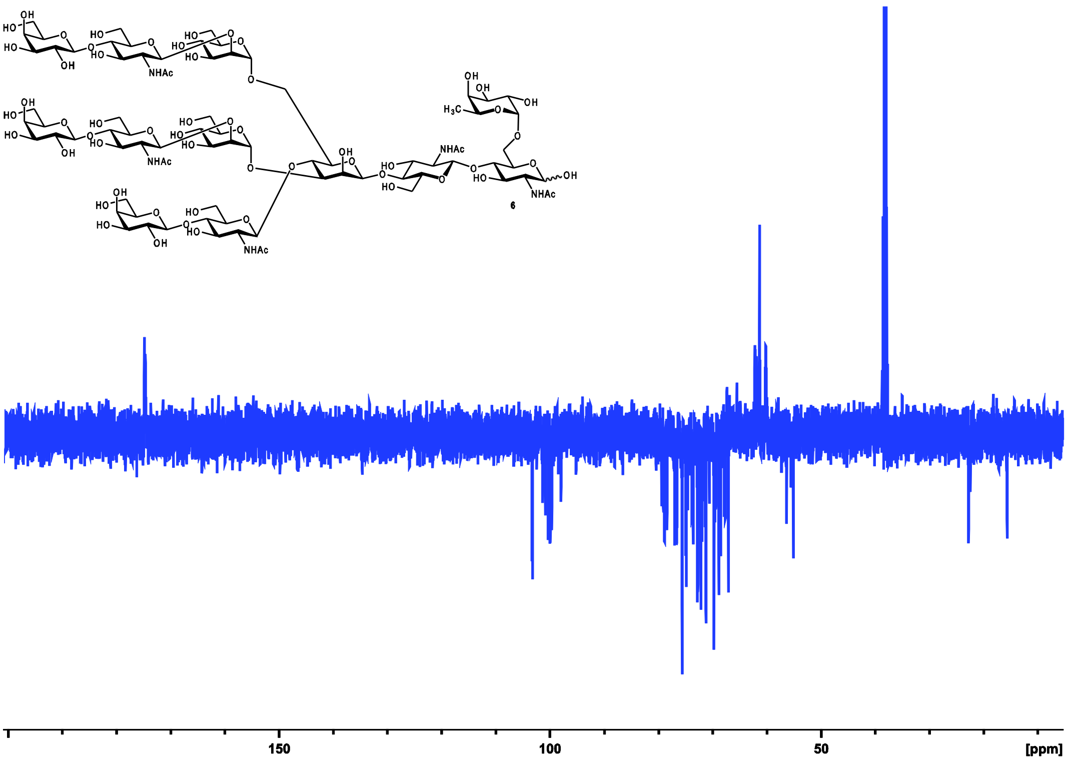

1H NMR, 500 MHz, D2O

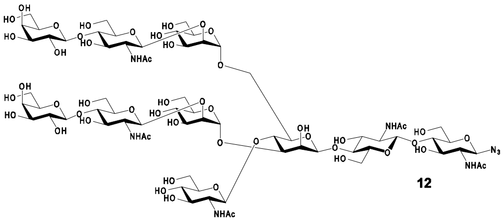

12

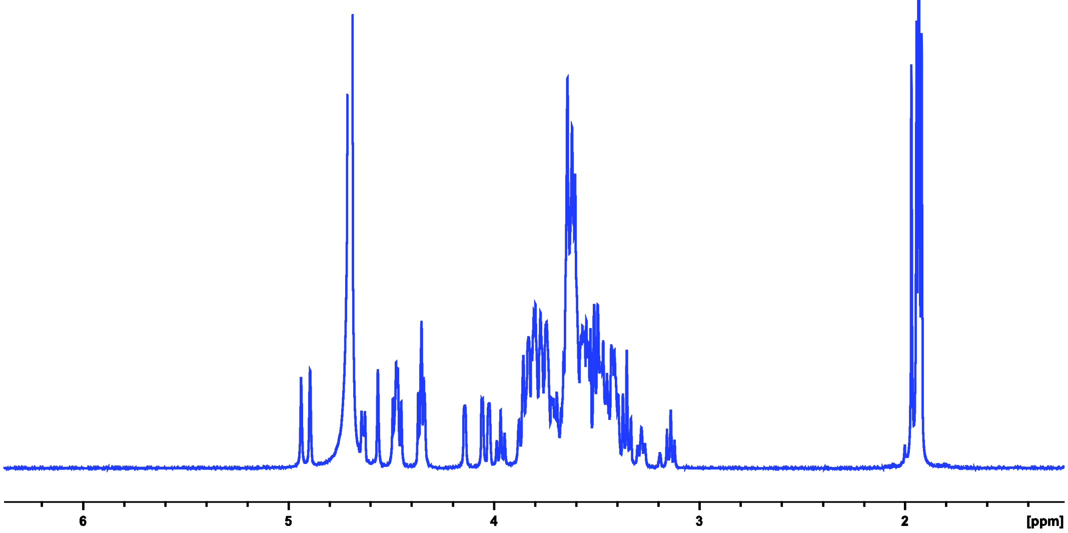

13C NMR, jmod, 125 MHz, D2O

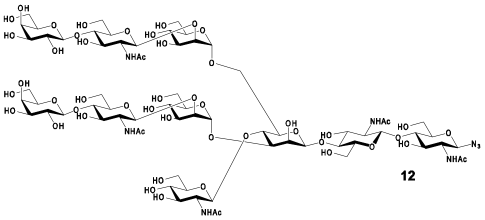

12

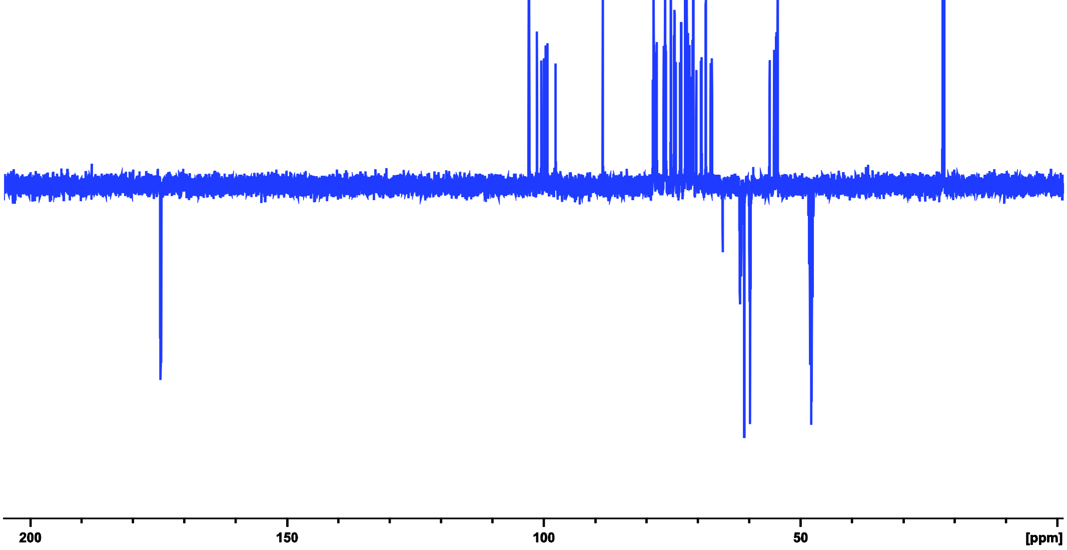

Phase-sensitive HSQC NMR, 500 MHz, D2O

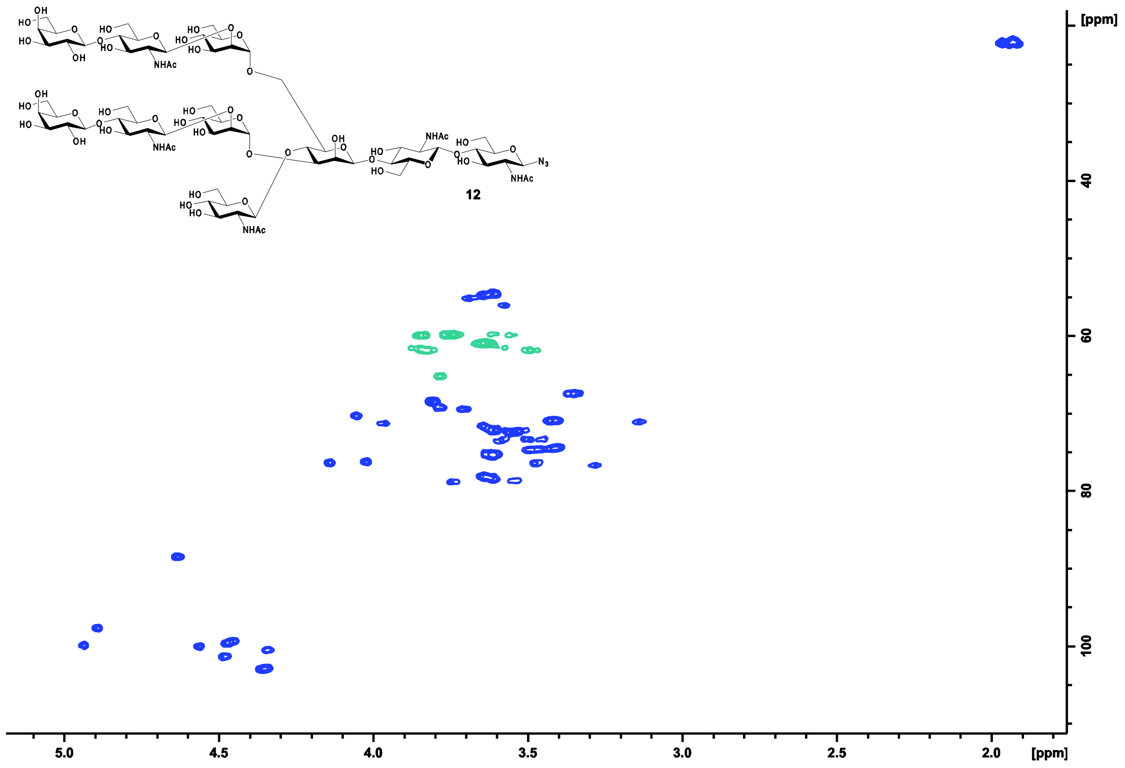

1H NMR, 500 MHz, D2O

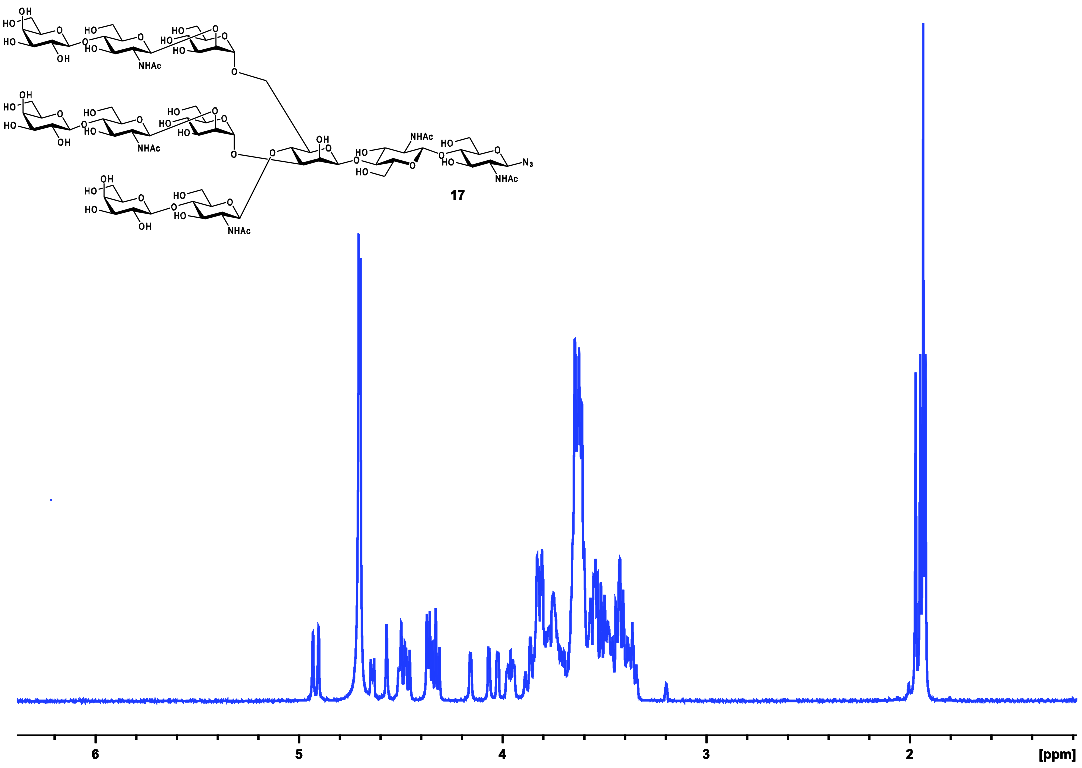

13C NMR, jmod, 125 MHz, D2O

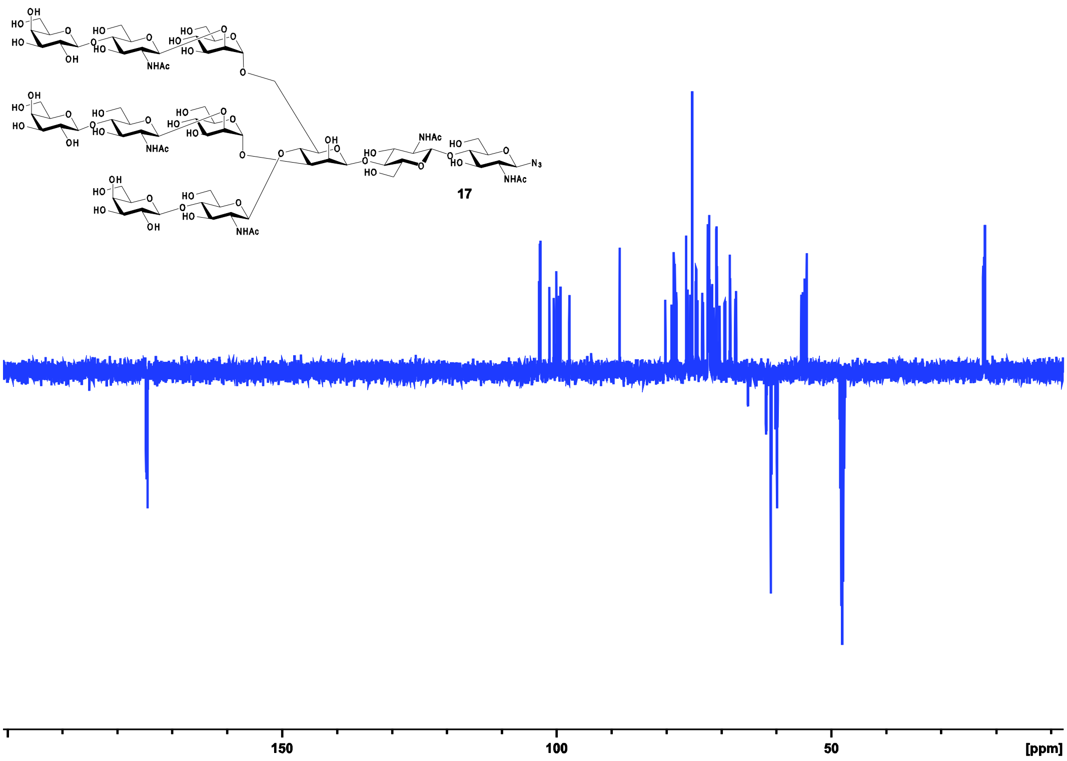

Phase-sensitive HSQC NMR, 500 MHz, D2O

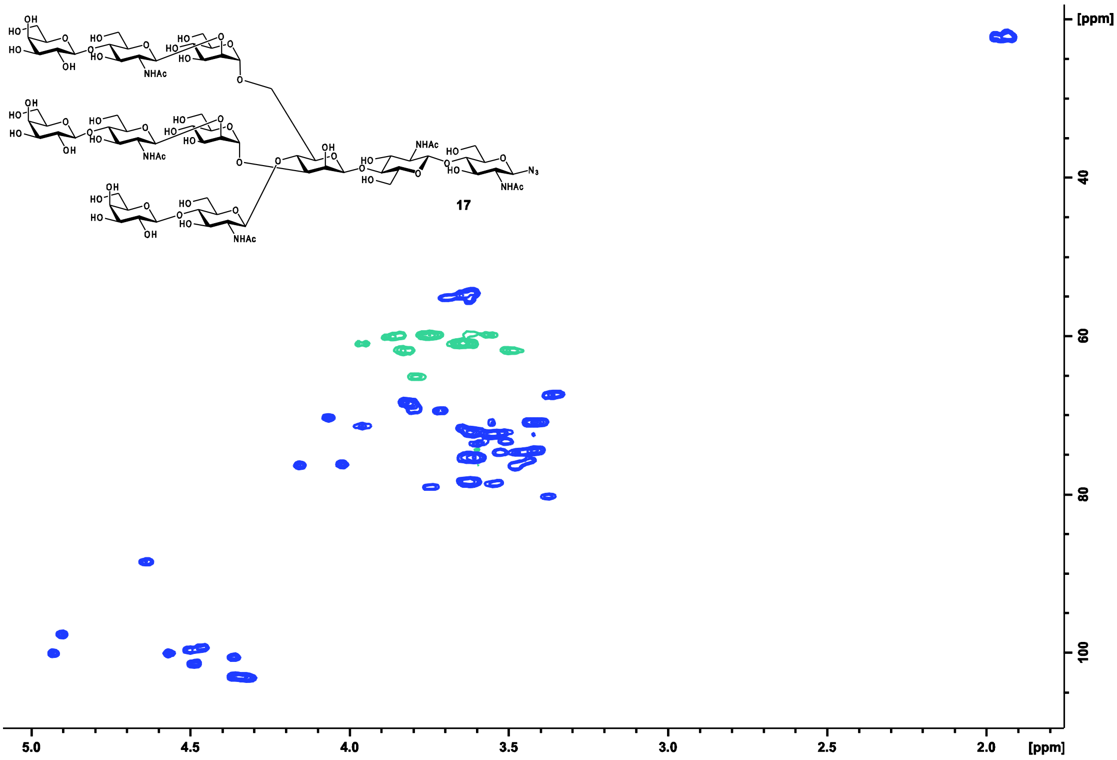

1H NMR, 500 MHz, D2O

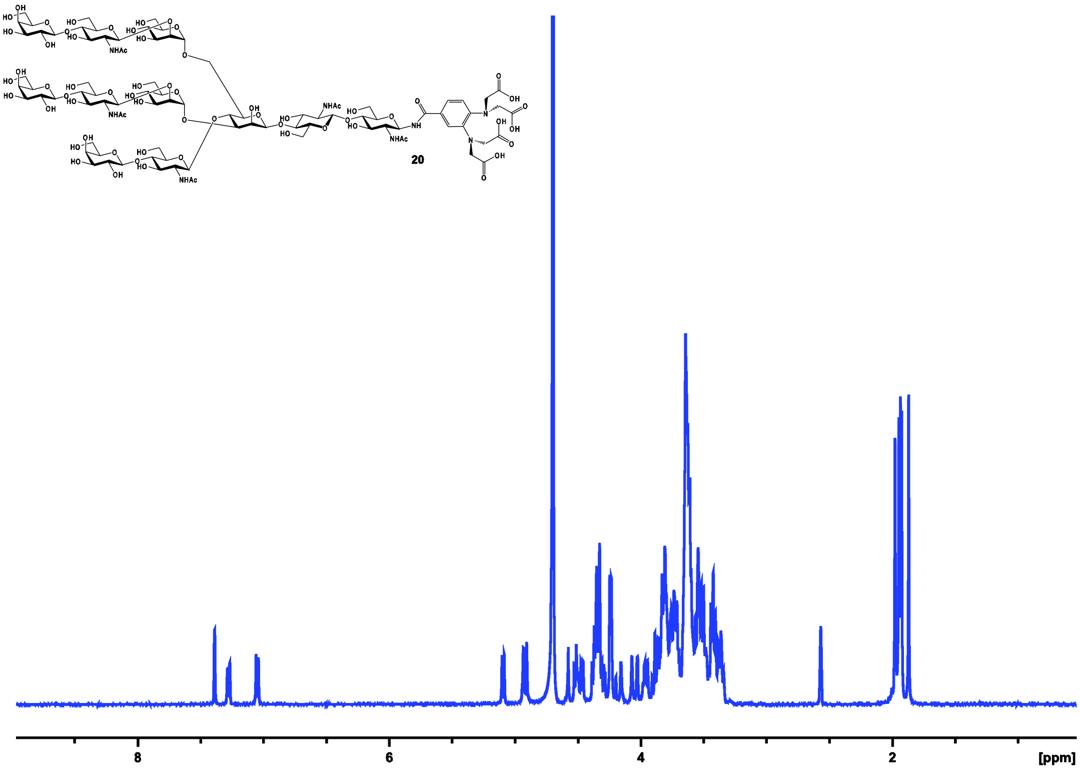

13C NMR, jmod, 125 MHz, D2O

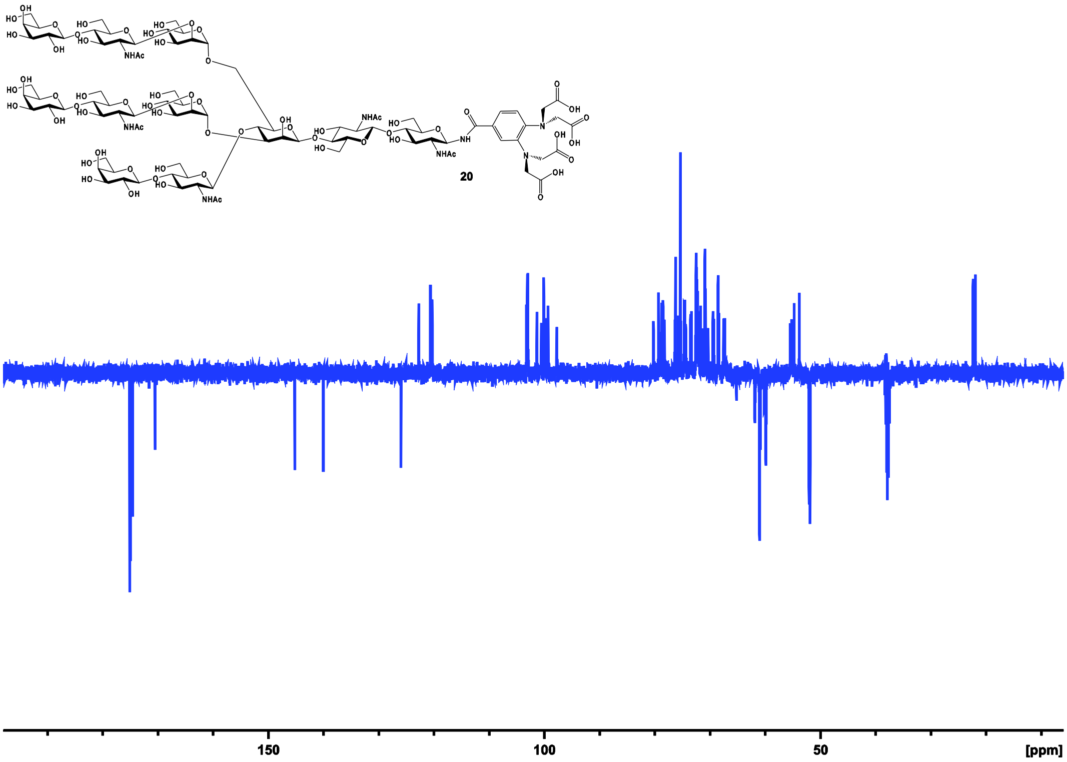

Phase-sensitive HSQC NMR, 500 MHz, D2O

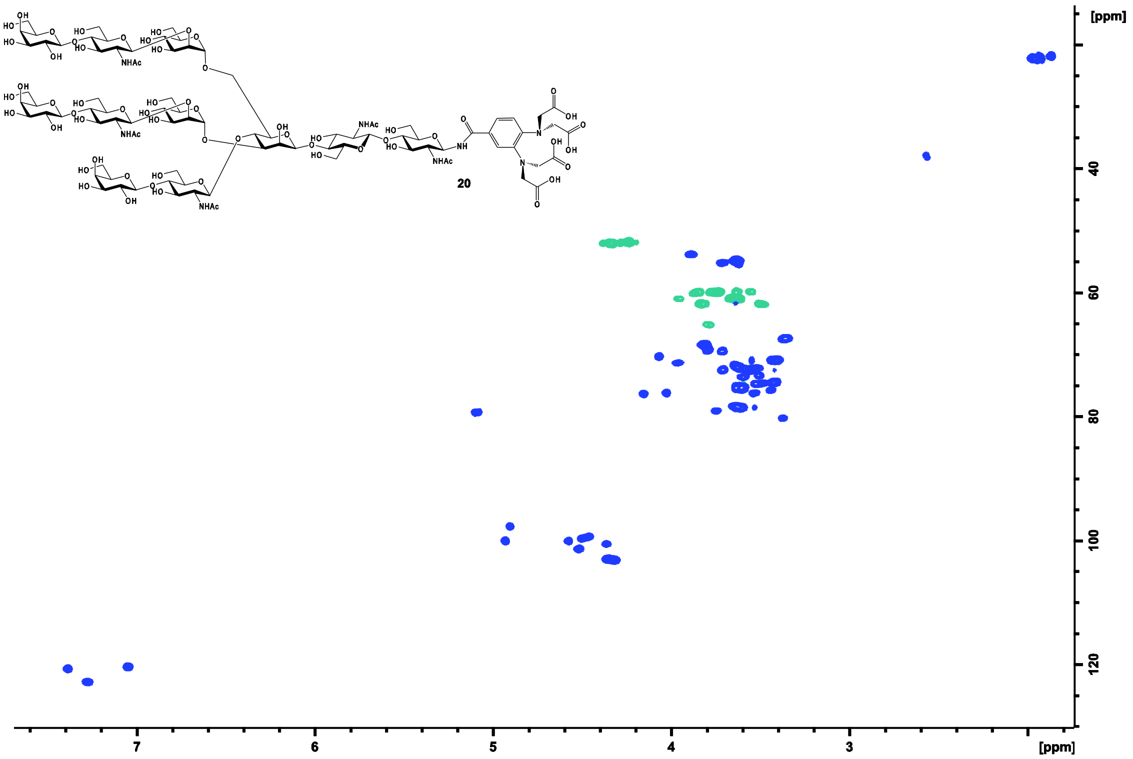

1H NMR, 500 MHz, D2O

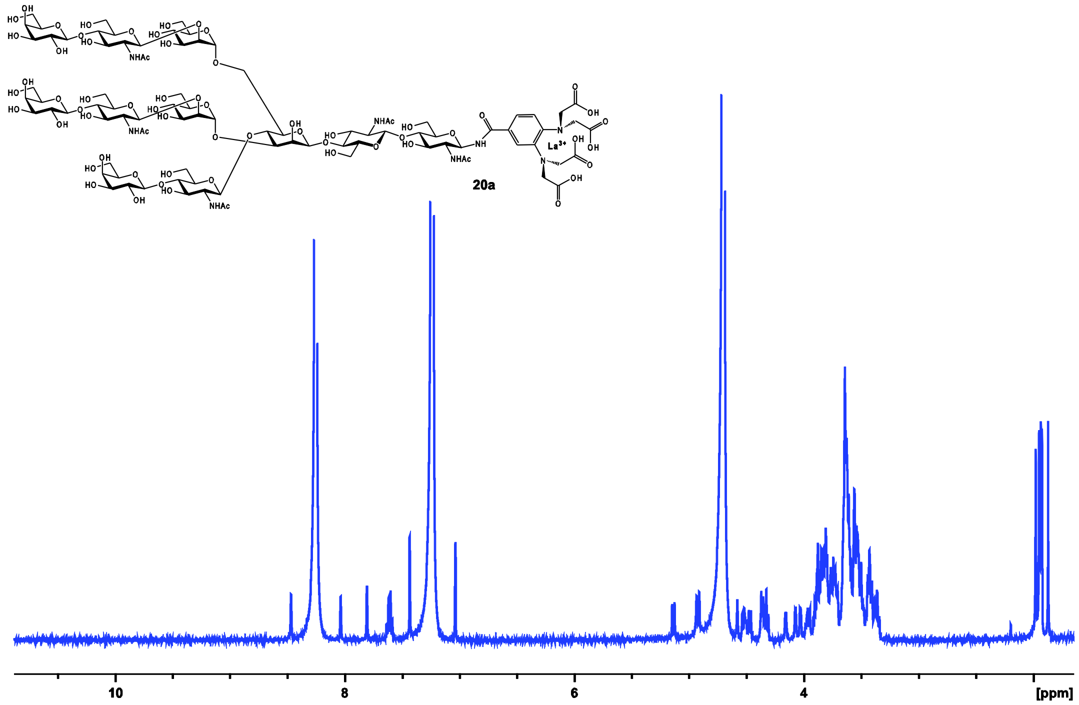

Phase-sensitive HSQC NMR, 500 MHz, D2O

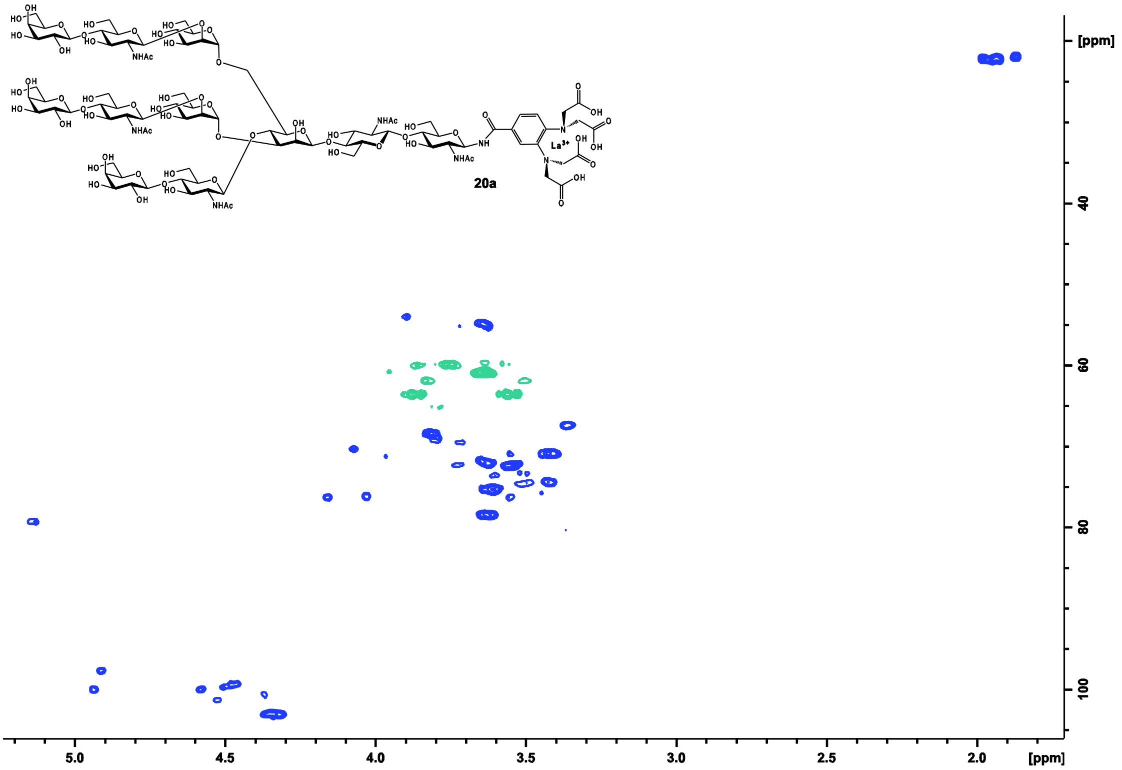

1H NMR, 500 MHz, D2O

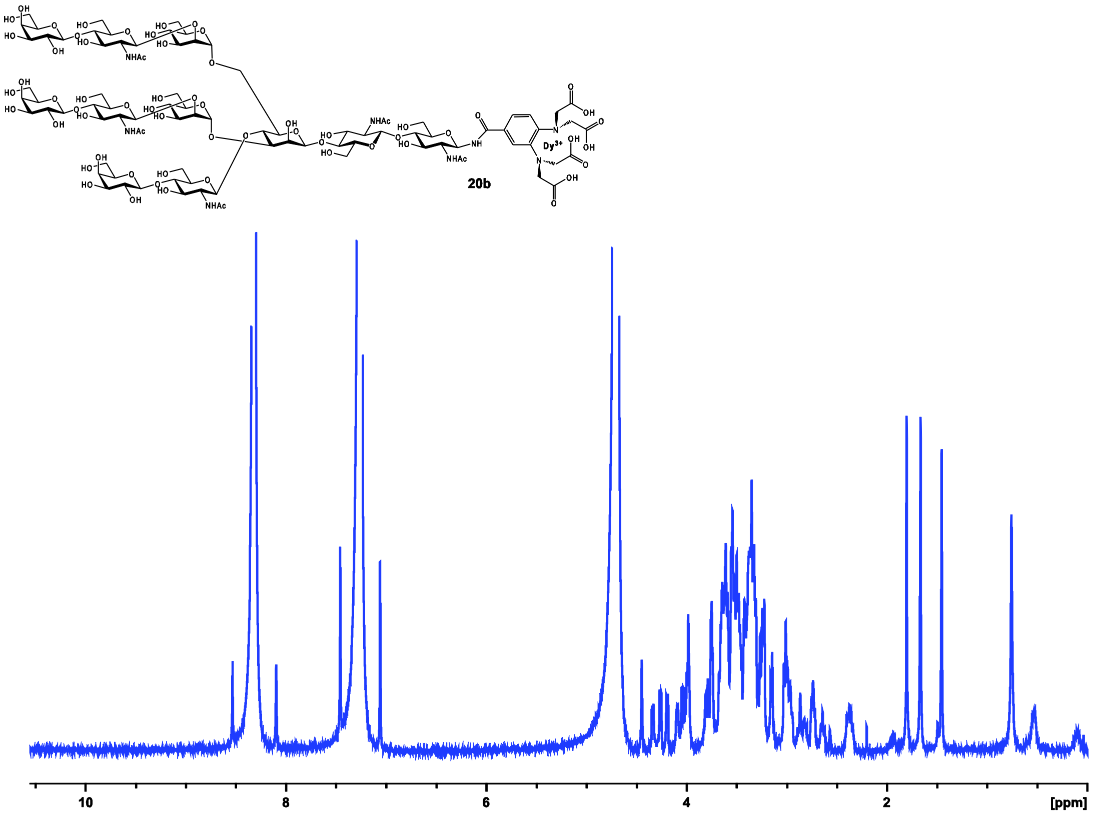

Phase-sensitive HSQC NMR, 500 MHz, D2O

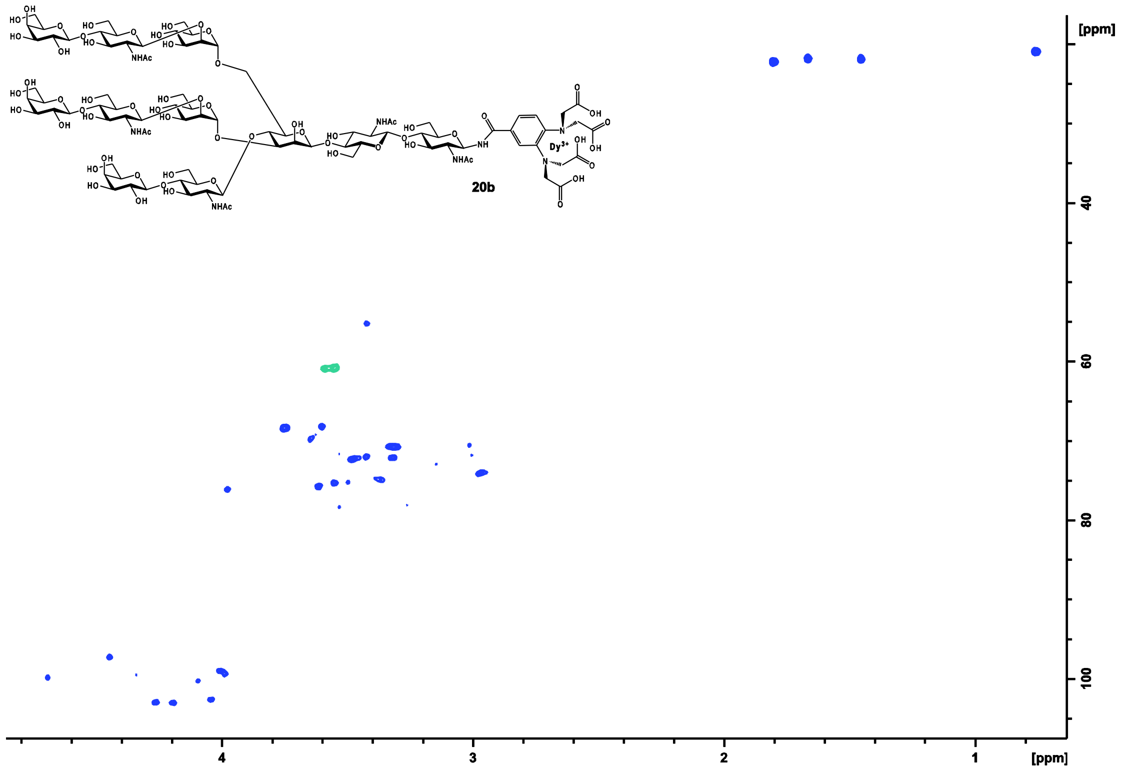

Pseudo contact shifts analysis

^{1}H-^{13}C HSQC NMR experiments were acquired for complexes **20a** and **20b,** on a Bruker 500 MHz spectrometer with 40 scans, at 293 K. 2048 points and 256 points were acquired for F2 and F1 dimensions, respectively. Pseudo contact shifts were obtained as the difference in chemical shift of each signal in the HSQCs of diamagnetic complex (**20a**) and paramagnetic complex (**20b**). The signal assignment of complexes **20a** and **20b** and the PCS values are given below.

Chemical shifts of lanthanum complex **20a**.

**Sugar Residue**	**Nucleus**	δ_H **(ppm)**	δ_C **(ppm)**	**Sugar Residue**	**Nucleus**	δ_H **(ppm)**	δ_C **(ppm)**
**GlcNAc1**	H1	5.14	79.24	**Man4**	H3	3.80	69.17
	H2	3.90	53.96		H4	3.36	67.31
	H3	3.73	72.24		H5	3.60	73.55
	H4	3.63	78.38		H6-H6′	---	---
	H5	3.55	76.24	**Man4′**	H1	4.91	97.63
	H6-H6′	---	---		H2	4.03	76.06
	Ac	1.87	21.88		H3	3.72	69.46
**GlcNAc2**	H1	4.53	101.24		H4	3.36	67.31
	H2	3.73	55.23		H5	3.51	73.29
	H3	3.64	72.05		H6-H6′	---	---
	H4	3.63	78.38	**GlcNAc5**	H1	4.49	99.56
	H5	3.51	74.47		H2	3.64	54.84
	H6-H6′	---	---		H3	3.64	72.05
	Ac	1.98	22.16		H4	3.63	78.38
**Man3**	H1	4.58	99.97		H5	3.51	74.47
	H2	4.07	70.33		H6-H6′	---	---
	H3	3.75	78.98		Ac	1.94	22.13
	H4	3.96	71.24	**GlcNAcB**	H1	4.37	100.53
	H5	3.42	74.39		H2	3.63	55.47
	H6-H6′	3.80	65.10		H3	3.64	72.05
**Man4**	H1	4.94	99.98		H4	3.38	80.27
	H2	4.16	76.24		H5	3.45	75.69
**Sugar**	**Nucleus**	δ_H **(ppm)**	δ_C **(ppm)**				

Residue			
**GlcNAcB**	H6-H6´	3.96	60.65
	Ac	1.94	22.13
**GlcNAc5´**	H1	4.49	99.56
	H2	3.64	54.84
	H3	3.64	72.05
	H4	3.63	78.38
	H5	3.42	74.39
	H6-H6´	---	---
	Ac	1.94	22.13
**Gal**	H1	4.34	103.01
	H2	3.43	70.84
	H3	3.56	72.30
	H4	3.82	68.45
	H5	3.62	75.18
	H6-H6´	---	---
**GalB**	H1	4.34	103.01
	H2	3.43	70.84
	H3	3.56	72.30
	H4	3.82	68.45
	H5	3.62	75.18
	H6-H6´	---	---
**Gal´**	H1	4.34	103.01
	H2	3.43	70.84
	H3	3.56	72.30
	H4	3.82	68.45
	H5	3.62	75.18
	H6-H6´	---	---

Chemical shifts of dysprosium complex **20b**.

Sugar Residue	Nucleus	δH (ppm)	δC (ppm)	Sugar Residue	Nucleus	δH (ppm)	δC (ppm)
**GlcNAc1**	H1	---	---	**GlcNAc5**	H1	4.34	99.50
	H2	---	---		H2	3.54	54.68
	H3	-0.53	72.11		H3	3.53	71.56
	H4	0.53	75.14		H4	3.53	78.29
	H5	0.09	68.58		H5	3.40	74.52
	H6-H6´	---	---		H6-H6´	---	---
	Ac	---	---		Ac	1.80	22.14
**GlcNAc2**	H1	2.74	99.53	**GlcNAcB**	H1	4.09	100.26
	H2	2.36	53.80		H2	3.42	55.16
	H3	2.64	70.99		H3	3.43	71.77
	H4	2.74	77.29		H4	3.23	79.99
	H5	2.39	73.36		H5	3.23	75.51
	H6-H6´	---	---		H6-H6´	3.80	60.58
	Ac	0.76	20.83		Ac	1.67	21.27
**Man3**	H1	3.99	99.43	**GlcNAc5´**	H1	4.01	98.90
	H2	3.65	69.67		H2	3.25	54.20
	H3	3.39	78.57		H3	3.01	71.83
	H4	3.63	70.78		H4	3.27	78.13
	H5	2.95	73.92		H5	2.98	74.17
	H6-H6´	3.38/3.34	64.61/64.75		H6-H6´	---	---
**Man4**	H1	4.70	99.81		Ac	1.46	21.86
	H2	3.98	76.06	**Gal**	H1	4.27	102.93
	H3	3.63	69.24		H2	3.32	70.70
	H4	3.25	67.36		H3	3.48	72.24
	H5	3.36	73.25		H4	3.75	68.34
	H6-H6´	---	---		H5	3.55	75.23
**Man4´**	H1	4.45	97.26		H6-H6´	---	---
	H2	3.61	75.72	**GalB**	H1	4.19	102.99
	H3	3.34	69.11		H2	3.32	70.70
	H4	3.02	66.90		H3	3.48	72.24
	H5	3.15	72.88		H4	3.75	68.34

	H6-H6´	---	---		H5	3.50	75.16

**Sugar Residue**	**Nucleus**	**δ_H (ppm)**	**δ_C (ppm)**
**GalB**	H6-H6´	---	---
**Gal´**	H1	4.04	102.52
	H2	3.01	70.51
	H3	3.32	72.15
	H4	3.60	68.10
	H5	3.37	74.94
	H6-H6´	---	---

Pseudo contact shifts

**Sugar Residue**	**Nucleus**	**PCS exp. (ppm)**	**Sugar Residue**	**Nucleus**	**PCS exp. (ppm)**
**GlcNAc1**	H1	---	**Man4´**	H6-H6´	**---**
	H2	---	**GlcNAc5**	H1	0.17
	H3	3.62		H2	0.10
	H4	3.11		H3	0.10
	H5	4.09		H4	0.09
	H6-H6´	---		H5	0.11
	Ac	---		H6-H6´	---
**GlcNAc2**	H1	1.78		Ac	0.14
	H2	1.38	**GlcNAcB**	H1	0.28
	H3	1.00		H2	0.20
	H4	0.89		H3	0.21
	H5	1.13		H4	0.16
	H6-H6´	---		H5	0.22
	Ac	1.22		H6-H6´	---
**Man3**	H1	0.59		Ac	0.27
	H2	0.43	**GlcNAc5´**	H1	0.46
	H3	0.36		H2	0.40
	H4	0.33		H3	0.62

	H5	0.48		H4	0.36
	H6-H6′	0.42-0.46		H5	0.44
**Man4**	H1	0.25		H6-H6′	---
	H2	0.18		Ac	0.48
	H3	0.18	**Gal**	H1	0.08
	H4	0.12		H2	0.11
	H5	0.24		H3	0.09
	H6-H6′	---		H4	0.06
**Man4′**	H1	0.47		H5	0.06
	H2	0.42		H6-H6′	---
	H3	0.38	**GalB**	H1	0.15
	H4	0.35		H2	0.11
	H5	0.36		H3	0.09

**Sugar Residue**	**Nucleus**	**PCS exp. (ppm)**
**GalB**	H4	0.06
	H5	0.12
	H6-H6′	---
**Gal′**	H1	0.30
	H2	0.41
	H3	0.24
	H4	0.21
	H5	0.25
	H6-H6′	---

Once the experimental values were obtained, Mspin software [2] was used to back-calculate the expected PCSs from the different oligosaccharide conformations. Initially, four conformations for the Man$^{4'}$α1,6-Man^{3} linkage were considered in the analysis: extended *gauche-gauche* (ψ= 180°, ω = 60°), extended *gauche-trans* (ψ= 180°, ω = 180°), folded *gauche-gauche* (ψ= 90°, ω = 60°) and folded *gauche-gauche* (ψ= 60°, ω = 60°). These conformations have been described as the most populated for a biantennary N-glycan with a

bisecting GlcNAc according to Molecular Dynamics simulations. [3] The conformations of the trigalactosylated bisected N-glycan were built with the server www.glycam.org and energy minimized in explicit water with AMBER16 (force field GLYCAM_06). [4] The system was equilibrated in an octaedric water box of 10 Å. TIP3P model for water molecules was used.

ω (O_1-O_6-C_6-C_5)	ψ (O_6-C_6-C_5-C_4)	Minimized Energy (kcal/mol)
+60° *gauche-gauche*, *gg*	180 (*extended*)	-3.26E+04
	90 (*backfold*)	-2.95E+04
	60 (*half backfold*)	-3.05E+04
+180° *gauche-trans*, *gt*	180 (*extended*)	-3.65E+04

Angle definition and energies of the minimized conformations of the trigalactosylated bisected N-glycan considered in the conformational analysis.

The geometry of the phenylene diamino tetraacetic chelating unit was obtained from the reported X-ray coordinates for this moiety. [5] Two conformations of the amide respect to the aromatic ring attached were considered for each glycan geometry to take into account the effect of the rotation of the chelating unit.

Then, combinations of the four glycan geometries with different populations were considered and their corresponding fittings were calculated by Mspin. The quality of the fitting between experimental and back-calculated values is given by the Q factor, which is defined by the following expression:

$$Q = \sqrt{\frac{\sum(PCS_{calc} - PCS_{\exp})^2}{\sum PCS_{\exp}{}^2}}$$

Iterative calculations were carried out with Mspin to obtain the number of conformers and the population of each conformer that better fit the experimental PCS values. The best correlation was obtained when three of the starting geometries were included in the calculation: extended *gauche-gauche*, extended *gauche-trans* and folded 90 *gauche-gauche* conformations. The

populations obtained with MSpin were 40 %, 28 % and 32 %, respectively, and the Q factor is 0.09.

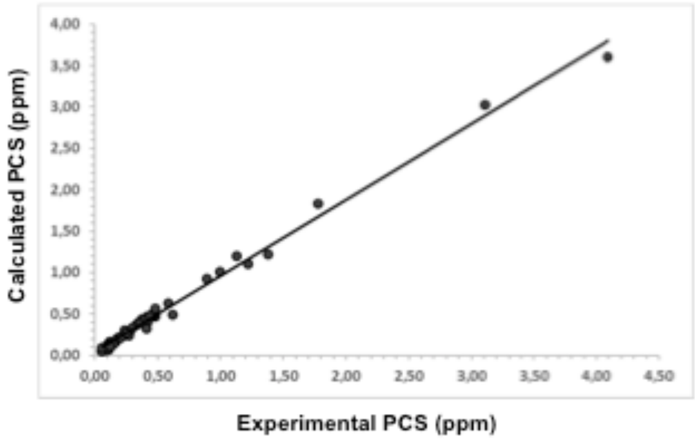

Glycan coordinates that better fit the experimental data according to Mspin calculations.

```
302
extended_gg_tagrot1
C  0.8408 -2.4822 23.2448
H  0.6152 -3.4449 23.6297
O  2.1881 -2.4370 22.6640
C  2.4358 -3.2994 21.4816
H  2.2702 -4.3425 21.7606
C  3.8921 -3.1140 21.0366
H  4.0902 -2.0705 20.7904
H  4.1006 -3.7206 20.1540
O  4.7631 -3.5274 22.0967
H  4.6124 -2.9720 22.8660
C  1.4279 -2.8948 20.3963
H  1.6292 -1.8551 20.1239
C -0.0201 -2.9876 20.9048
H -0.2595 -4.0226 21.1572
O -0.9113 -2.5426 19.8648
H -1.8193 -2.6086 20.1702
C -0.1994 -2.0834 22.1381
H -0.0145 -1.0497 21.8335
N -1.5735 -2.1885 22.6538
H -1.8121 -3.0559 23.1139
C -2.5186 -1.2392 22.5231
O -2.3385 -0.1602 21.9537
C -3.8904 -1.5870 23.1332
H -3.7651 -2.0809 24.0978
H -4.4739 -0.6767 23.2822
H -4.4331 -2.2480 22.4568
O  1.5944 -3.7736 19.2180
C  1.9781 -3.0481 17.9964
H  2.9221 -2.5293 18.1712
O  0.9341 -2.0588 17.6458
C  1.1265 -1.2739 16.4175
H  2.0588 -0.7097 16.4922
C -0.0507 -0.2974 16.2805
H -0.9961 -0.8398 16.2259
H  0.0548  0.3017 15.3754
O -0.0709  0.5773 17.4159
H -0.1999  0.0598 18.2140
C  1.2242 -2.2899 15.2753
H  0.2842 -2.8484 15.2360
C  2.3644 -3.2941 15.5017
H  3.3227 -2.7715 15.5207
O  2.3668 -4.2380 14.4144
H  3.0950 -4.8525 14.5258
C  2.1329 -4.0427 16.8294
H  1.2051 -4.6129 16.7469
N  3.2398 -4.9729 17.0903
H  4.1024 -4.5625 17.4158
C  3.1947 -6.2992 16.8590
O  2.1979 -6.8911 16.4366
C  4.5028 -7.0631 17.1350
H  4.9911 -6.6785 18.0319
H  4.2906 -8.1236 17.2814
H  5.1741 -6.9525 16.2824
O  1.4413 -1.5583 14.0214
C  0.3074 -1.6812 13.1005
H -0.6131 -1.4756 13.6502
O  0.2332 -3.0483 12.5382
C -0.8377 -3.3092 11.5577
H -1.8020 -3.1447 12.0421
C -0.7380 -4.7880 11.1658
H -1.5790 -5.0741 10.5337
H -0.7517 -5.4115 12.0615
O  0.5166 -4.9921 10.4321
C -0.6600 -2.2970 10.4139
H  0.3115 -2.4762  9.9462
O -1.7425 -2.4709  9.4311
C -0.6645 -0.8541 10.9357
H -1.6293 -0.6494 11.4046
C  0.4407 -0.6392 11.9813
H  0.3572  0.3624 12.4031
O  1.7366 -0.7699 11.3579
H  2.4243 -0.6490 12.0176
O -0.4336  0.0790  9.8219
C  0.9770 -6.3744 10.2528
H  0.9868 -6.8702 11.2240
C  2.4133 -6.2949  9.7192
H  2.9554 -5.5529 10.3107
C  2.4660 -5.8620  8.2406
H  2.1736 -4.8117  8.1813
C  1.5386 -6.7015  7.3446
H  1.9272 -7.7168  7.2468
C  0.0974 -6.7419  7.8909
H -0.3256 -5.7422  7.8277
C -0.8279 -7.7072  7.1403
H -0.8109 -7.4975  6.0702
H -0.5171 -8.7412  7.2946
O -2.1666 -7.5305  7.6217
H -2.4435 -6.6236  7.4698
O  0.1240 -7.1459  9.3183
O  1.4941 -6.0763  6.0495
H  2.3836 -6.0226  5.6917
O  3.8181 -5.9843  7.7626
H  4.4058 -5.4875  8.3370
O  3.0486 -7.6217  9.8953
C  4.2240 -7.6488 10.7869
H  3.9210 -7.3399 11.7889
O  5.2809 -6.7398 10.2890
C  6.5591 -6.7044 11.0313
H  6.3638 -6.4034 12.0629
C  7.4660 -5.6651 10.3621
H  7.6395 -5.9226  9.3162
H  8.4287 -5.6145 10.8698
O  6.8352 -4.3794 10.4347
H  5.9932 -4.4109  9.9745
C  7.1314 -8.1298 11.0101
H  7.3299 -8.4028  9.9691
C  6.1272 -9.1363 11.5945
H  5.9564 -8.9174 12.6504
O  6.6853 -10.4585 11.4767
H  6.0802 -11.0964 11.8610
C  4.7949 -9.0812 10.8185
H  4.9882 -9.3836  9.7867
N  3.8198 -10.0092 11.4094
H  3.4126 -9.7306 12.2907
C  3.4949 -11.2090 10.8913
O  3.9498 -11.6481  9.8326
C  2.5094 -12.0453 11.7297
H  1.7111 -11.4151 12.1242
H  2.0639 -12.8271 11.1121
H  3.0434 -12.5125 12.5580
O  8.3850 -8.1641 11.7939
C  9.5765 -8.5066 11.0009
H  9.6179 -7.8519 10.1282
O  9.5175 -9.9108 10.5435
C 10.6663 -10.3850  9.7421
H 10.7842 -9.7237  8.8810
C 10.3433 -11.7905  9.2169
H 10.1292 -12.4732 10.0408
H 11.1850 -12.1858  8.6492
O  9.1958 -11.7115  8.3605
H  8.4520 -11.3578  8.8548
C 11.9379 -10.2736 10.6088
H 12.8118 -10.5589 10.0227
O 11.8095 -11.1762 11.7300
H 12.5918 -11.1113 12.2829
C 12.0979 -8.8261 11.1243
H 12.2981 -8.1859 10.2628
O 13.2372 -8.7566 12.0009
H 13.3693 -7.8489 12.2827
C 10.8421 -8.3041 11.8538
H 10.7262 -8.8051 12.8160
O 10.9956 -6.8898 12.0667
H 10.2225 -6.5499 12.5243
C -1.2396 -2.9789  8.1496
H -0.6423 -3.8674  8.3428
O -0.3889 -1.9702  7.4879
C  0.2057 -2.3753  6.2019
H  0.7933 -3.2861  6.3470
C  1.1326 -1.2436  5.7497
H  0.5861 -0.3022  5.7040
H  1.5483 -1.4565  4.7644
O  2.2063 -1.1221  6.6932
H  1.8464 -0.9390  7.5645
C -0.9656 -2.6659  5.2516
H -1.5448 -1.7449  5.1384
C -1.8869 -3.7543  5.8318
H -1.3397 -4.6967  5.9059
O -3.0081 -3.9359  4.9497
H -3.5727 -4.6333  5.2904
C -2.4113 -3.3347  7.2202
H -3.0363 -2.4467  7.1064
N -3.2053 -4.4161  7.8159
H -2.6914 -5.2271  8.1293
C -4.5458 -4.4071  7.9409
O -5.2644 -3.4752  7.5779
C -5.1555 -5.6762  8.5664
H -4.5480 -6.0194  9.4065
H -6.1634 -5.4671  8.9280
H -5.2061 -6.4635  7.8142
O -0.4349 -3.1034  3.9443
C -0.8186 -2.2166  2.8360
H -0.5708 -1.1893  3.1072
O -2.2682 -2.3072  2.5737
C -2.7842 -1.4404  1.4945
H -2.5002 -0.4117  1.7186
C -4.3172 -1.5217  1.4908
H -4.6512 -2.5396  1.2839
H -4.7287 -0.8585  0.7303
O -4.8095 -1.1234  2.7764
H -4.4408 -1.6982  3.4526
C -2.0911 -1.8477  0.1776
H -2.4035 -1.1805 -0.6263
O -2.4910 -3.1970 -0.1500
H -2.0668 -3.4657 -0.9684
C -0.5569 -1.7883  0.3491
H -0.2760 -0.7429  0.4880
O  0.0743 -2.2606 -0.8549
H  1.0275 -2.1979 -0.7616
C -0.0534 -2.6042  1.5593
H -0.1596 -3.6744  1.3696
O  1.3335 -2.2924  1.7768
H  1.6638 -2.8091  2.5157
C -1.5658  0.9436  9.4715
H -2.4274  0.3151  9.2447
C -1.1768  1.7271  8.2142
H -0.6999  1.0377  7.5132
C -0.1936  2.8812  8.4965
H  0.7830  2.4493  8.7236
C -0.6423  3.7696  9.6698
H -1.5286  4.3457  9.3944
C -0.9326  2.9262 10.9252
H -0.0079  2.4381 11.2412
C -1.5031  3.7204 12.1068
H -0.8677  4.5745 12.3387
H -2.5053  4.0862 11.8810
O -1.5540  2.8614 13.2546
H -0.6663  2.5725 13.4766
O -1.9190  1.8797 10.5661
O  0.4344  4.6707  9.9830
H  0.6340  5.2083  9.2138
O -0.0686  3.6914  7.3121
```

```
H  0.1869  3.1372  6.5719
O -2.4241  2.2531  7.6217
C -2.7660  1.7033  6.3016
H -2.8909  0.6220  6.3833
O -1.6967  2.0050  5.3221
C -1.9238  1.5929  3.9242
H -2.0693  0.5116  3.8902
C -0.6815  1.9694  3.1045
H -0.4998  3.0441  3.1562
H -0.8162  1.6906  2.0603
O  0.4575  1.2733  3.6238
H  0.5975  1.5266  4.5390
C -3.2004  2.3043  3.4543
H -3.0230  3.3838  3.4904
C -4.3915  1.9807  4.3670
H -4.6270  0.9179  4.3068
O -5.5302  2.7372  3.9173
H -6.2904  2.5269  4.4643
C -4.0769  2.3594  5.8251
H -3.9495  3.4426  5.8810
N -5.1906  1.9643  6.7005
H -5.2747  0.9774  6.8990
C -6.1256  2.8058  7.1796
O -6.1225  4.0233  6.9840
C -7.2544  2.1432  7.9938
H -6.8488  1.3816  8.6610
H -7.7730  2.8928  8.5931
H -7.9696  1.6812  7.3120
O -3.5053  1.8817  2.0742
C -3.4300  2.9717  1.0930
H -2.4862  3.5040  1.2264
O -4.5464  3.9194  1.2836
C -4.6037  5.0596  0.3454
H -3.6562  5.6000  0.3993
C -5.7180  6.0076  0.8088
H -6.6746  5.4865  0.8608
H -5.8147  6.8452  0.1180
O -5.3849  6.5106  2.1104
H -5.2922  5.7765  2.7228
C -4.7540  4.4966 -1.0844
H -4.7390  5.3138 -1.8062
O -6.0231  3.8125 -1.1752
H -6.1208  3.4386 -2.0541
C -3.6081  3.5045 -1.3837
H -2.6743  4.0702 -1.4059
O -3.8064  2.9271 -2.6866
H -3.0674  2.3510 -2.8956
C -3.4885  2.3859 -0.3284
H -4.3264  1.6913 -0.4107
O -2.2573  1.6761 -0.5505
H -2.1631  0.9841  0.1075
N  2.0349  1.7968 29.9491
O  3.2075  2.8977 31.9853
O  3.3196  4.8967 31.0245
O  1.1747  0.6253 32.2214
O -0.9292  1.2948 31.9449
C  1.8795  0.9040 28.8220
C  1.3083  1.3562 27.6069
H  1.0475  2.4078 27.4864
C  1.0816  0.4679 26.5749
H  0.6443  0.8210 25.6410
C  2.8325  3.0073 29.6337
H  2.2552  3.6850 29.0047
H  3.7521  2.7284 29.1196
C  3.1540  3.6699 30.9592
C  0.6932  2.1892 30.4574
H -0.0308  2.1014 29.6474
H  0.7335  3.2235 30.7989
C  0.2477  1.3030 31.6161
N  2.9192 -0.8484 30.1519
O  3.4052 -1.0438 32.6929
```

```
O  2.9682 -3.2203 32.7571
O  4.8500  0.9075 30.8337
O  6.3922 -0.0683 29.5611
C  2.2324 -0.4148 28.9550
C  1.9780 -1.3175 27.8929
H  2.2346 -2.3715 28.0014
C  1.4060 -0.8652 26.7210
C  2.2536 -1.9867 30.8313
H  2.3948 -2.9022 30.2571
H  1.1876 -1.7875 30.9422
C  2.9061 -2.1177 32.1937
C  4.3225 -1.2070 29.8136
H  4.3714 -1.4875 28.7615
H  4.6293 -2.0519 30.4302
C  5.2770 -0.0431 30.0599
N  0.9234 -1.5010 24.3236
C  1.1964 -1.8897 25.6038
O  1.2858 -3.0703 25.8571
X  3.1044  0.8166 31.7817
H  0.8510 -0.5408 24.0599
```

302

extended_gg_tagrot2

```
C  0.8408 -2.4822 23.2448
H  0.6152 -3.4449 23.6297
O  2.1881 -2.4370 22.6640
C  2.4358 -3.2994 21.4816
H  2.2702 -4.3425 21.7606
C  3.8921 -3.1140 21.0366
H  4.0902 -2.0705 20.7904
H  4.1006 -3.7206 20.1540
O  4.7631 -3.5274 22.0967
H  4.6124 -2.9720 22.8660
C  1.4279 -2.8948 20.3963
H  1.6292 -1.8551 20.1239
C -0.0201 -2.9876 20.9048
H -0.2595 -4.0226 21.1572
O -0.9113 -2.5426 19.8648
H -1.8193 -2.6086 20.1702
C -0.1994 -2.0834 22.1381
H -0.0145 -1.0497 21.8335
N -1.5735 -2.1885 22.6538
H -1.8121 -3.0559 23.1139
C -2.5186 -1.2392 22.5231
O -2.3385 -0.1602 21.9537
C -3.8904 -1.5870 23.1332
H -3.7651 -2.0809 24.0978
H -4.4739 -0.6767 23.2822
H -4.4331 -2.2480 22.4568
O  1.5944 -3.7736 19.2180
C  1.9781 -3.0481 17.9964
H  2.9221 -2.5293 18.1712
O  0.9341 -2.0588 17.6458
C  1.1265 -1.2739 16.4175
H  2.0588 -0.7097 16.4922
C -0.0507 -0.2974 16.2805
H -0.9961 -0.8398 16.2259
H  0.0548  0.3017 15.3754
O -0.0709  0.5773 17.4159
H -0.1999  0.0598 18.2140
C  1.2242 -2.2899 15.2753
H  0.2842 -2.8484 15.2360
C  2.3644 -3.2941 15.5017
H  3.3227 -2.7715 15.5207
O  2.3668 -4.2380 14.4144
H  3.0950 -4.8525 14.5258
C  2.1329 -4.0427 16.8294
H  1.2051 -4.6129 16.7469
N  3.2398 -4.9729 17.0903
H  4.1024 -4.5625 17.4158
C  3.1947 -6.2992 16.8590
O  2.1979 -6.8911 16.4366
C  4.5028 -7.0631 17.1350
H  4.9911 -6.6785 18.0319
H  4.2906 -8.1236 17.2814
H  5.1741 -6.9525 16.2824
O  1.4413 -1.5583 14.0214
C  0.3074 -1.6812 13.1005
H -0.6131 -1.4756 13.6502
O  0.2332 -3.0483 12.5382
C -0.8377 -3.3092 11.5577
H -1.8020 -3.1447 12.0421
C -0.7380 -4.7880 11.1658
H -1.5790 -5.0741 10.5337
H -0.7517 -5.4115 12.0615
O  0.5166 -4.9921 10.4321
C -0.6600 -2.2970 10.4139
H  0.3115 -2.4762  9.9462
O -1.7425 -2.4709  9.4311
C -0.6645 -0.8541 10.9357
H -1.6293 -0.6494 11.4046
C  0.4407 -0.6392 11.9813
H  0.3572  0.3624 12.4031
O  1.7366 -0.7699 11.3579
H  2.4243 -0.6490 12.0176
O -0.4336  0.0790  9.8219
C  0.9770 -6.3744 10.2528
H  0.9868 -6.8702 11.2240
C  2.4133 -6.2949  9.7192
H  2.9554 -5.5529 10.3107
C  2.4660 -5.8620  8.2406
H  2.1736 -4.8117  8.1813
C  1.5386 -6.7015  7.3446
H  1.9272 -7.7168  7.2468
C  0.0974 -6.7419  7.8909
H -0.3256 -5.7422  7.8277
C -0.8279 -7.7072  7.1403
H -0.8109 -7.4975  6.0702
H -0.5171 -8.7412  7.2946
O -2.1666 -7.5305  7.6217
H -2.4435 -6.6236  7.4698
O  0.1240 -7.1459  9.3183
O  1.4941 -6.0763  6.0495
H  2.3836 -6.0226  5.6917
O  3.8181 -5.9843  7.7626
H  4.4058 -5.4875  8.3370
O  3.0486 -7.6217  9.8953
C  4.2240 -7.6488 10.7869
H  3.9210 -7.3399 11.7889
O  5.2809 -6.7398 10.2890
C  6.5591 -6.7044 11.0313
H  6.3638 -6.4034 12.0629
C  7.4660 -5.6651 10.3621
H  7.6395 -5.9226  9.3162
H  8.4287 -5.6145 10.8698
O  6.8352 -4.3794 10.4347
H  5.9932 -4.4109  9.9745
C  7.1314 -8.1298 11.0101
H  7.3299 -8.4028  9.9691
C  6.1272 -9.1363 11.5945
H  5.9564 -8.9174 12.6504
O  6.6853 -10.4585 11.4767
H  6.0802 -11.0964 11.8610
C  4.7949 -9.0812 10.8185
H  4.9882 -9.3836  9.7867
N  3.8198 -10.0092 11.4094
H  3.4126 -9.7306 12.2907
C  3.4949 -11.2090 10.8913
O  3.9498 -11.6481  9.8326
C  2.5094 -12.0453 11.7297
H  1.7111 -11.4151 12.1242
H  2.0639 -12.8271 11.1121
H  3.0434 -12.5125 12.5580
O  8.3850 -8.1641 11.7939
C  9.5765 -8.5066 11.0009
H  9.6179 -7.8519 10.1282
O  9.5175 -9.9108 10.5435
C 10.6663 -10.3850  9.7421
H 10.7842 -9.7237  8.8810
C 10.3433 -11.7905  9.2169
H 10.1292 -12.4732 10.0408
H 11.1850 -12.1858  8.6492
O  9.1958 -11.7115  8.3605
H  8.4520 -11.3578  8.8548
C 11.9379 -10.2736 10.6088
H 12.8118 -10.5589 10.0227
O 11.8095 -11.1762 11.7300
H 12.5918 -11.1113 12.2829
C 12.0979 -8.8261 11.1243
H 12.2981 -8.1859 10.2628
O 13.2372 -8.7566 12.0009
H 13.3693 -7.8489 12.2827
C 10.8421 -8.3041 11.8538
H 10.7262 -8.8051 12.8160
O 10.9956 -6.8898 12.0667
H 10.2225 -6.5499 12.5243
C -1.2396 -2.9789  8.1496
H -0.6423 -3.8674  8.3428
O -0.3889 -1.9702  7.4879
C  0.2057 -2.3753  6.2019
H  0.7933 -3.2861  6.3470
C  1.1326 -1.2436  5.7497
H  0.5861 -0.3022  5.7040
H  1.5483 -1.4565  4.7644
O  2.2063 -1.1221  6.6932
H  1.8464 -0.9390  7.5645
C -0.9656 -2.6659  5.2516
H -1.5448 -1.7449  5.1384
C -1.8869 -3.7543  5.8318
H -1.3397 -4.6967  5.9059
O -3.0081 -3.9359  4.9497
H -3.5727 -4.6333  5.2904
C -2.4113 -3.3347  7.2202
H -3.0363 -2.4467  7.1064
N -3.2053 -4.4161  7.8159
H -2.6914 -5.2271  8.1293
C -4.5458 -4.4071  7.9409
O -5.2644 -3.4752  7.5779
C -5.1555 -5.6762  8.5664
H -4.5480 -6.0194  9.4065
H -6.1634 -5.4671  8.9280
H -5.2061 -6.4635  7.8142
O -0.4349 -3.1034  3.9443
C -0.8186 -2.2166  2.8360
H -0.5708 -1.1893  3.1072
O -2.2682 -2.3072  2.5737
C -2.7842 -1.4404  1.4945
H -2.5002 -0.4117  1.7186
C -4.3172 -1.5217  1.4908
H -4.6512 -2.5396  1.2839
H -4.7287 -0.8585  0.7303
O -4.8095 -1.1234  2.7764
H -4.4408 -1.6982  3.4526
C -2.0911 -1.8477  0.1776
H -2.4035 -1.1805 -0.6263
O -2.4910 -3.1970 -0.1500
H -2.0668 -3.4657 -0.9684
C -0.5569 -1.7883  0.3491
H -0.2760 -0.7429  0.4880
O  0.0743 -2.2606 -0.8549
H  1.0275 -2.1979 -0.7616
C -0.0534 -2.6042  1.5593
H -0.1596 -3.6744  1.3696
O  1.3335 -2.2924  1.7768
H  1.6638 -2.8091  2.5157
C -1.5658  0.9436  9.4715
H -2.4274  0.3151  9.2447
C -1.1768  1.7271  8.2142
H -0.6999  1.0377  7.5132
C -0.1936  2.8812  8.4965
H  0.7830  2.4493  8.7236
C -0.6423  3.7696  9.6698
H -1.5286  4.3457  9.3944
C -0.9326  2.9262 10.9252
H -0.0079  2.4381 11.2412
C -1.5031  3.7204 12.1068
H -0.8677  4.5745 12.3387
H -2.5053  4.0862 11.8810
O -1.5540  2.8614 13.2546
H -0.6663  2.5725 13.4766
O -1.9190  1.8797 10.5661
O  0.4344  4.6707  9.9830
H  0.6340  5.2083  9.2138
O -0.0686  3.6914  7.3121
```

H	0.1869	3.1372	6.5719
O	-2.4241	2.2531	7.6217
C	-2.7660	1.7033	6.3016
H	-2.8909	0.6220	6.3833
O	-1.6967	2.0050	5.3221
C	-1.9238	1.5929	3.9242
H	-2.0693	0.5116	3.8902
C	-0.6815	1.9694	3.1045
H	-0.4998	3.0441	3.1562
H	-0.8162	1.6906	2.0603
O	0.4575	1.2733	3.6238
H	0.5975	1.5266	4.5390
C	-3.2004	2.3043	3.4543
H	-3.0230	3.3838	3.4904
C	-4.3915	1.9807	4.3670
H	-4.6270	0.9179	4.3068
O	-5.5302	2.7372	3.9173
H	-6.2904	2.5269	4.4643
C	-4.0769	2.3594	5.8251
H	-3.9495	3.4426	5.8810
N	-5.1906	1.9643	6.7005
H	-5.2747	0.9774	6.8990
C	-6.1256	2.8058	7.1796
O	-6.1225	4.0233	6.9840
C	-7.2544	2.1432	7.9938
H	-6.8488	1.3816	8.6610
H	-7.7730	2.8928	8.5931
H	-7.9696	1.6812	7.3120
O	-3.5053	1.8817	2.0742
C	-3.4300	2.9717	1.0930
H	-2.4862	3.5040	1.2264
O	-4.5464	3.9194	1.2836
C	-4.6037	5.0596	0.3454
H	-3.6562	5.6000	0.3993
C	-5.7180	6.0076	0.8088
H	-6.6746	5.4865	0.8608
H	-5.8147	6.8452	0.1180
O	-5.3849	6.5106	2.1104
H	-5.2922	5.7765	2.7228
C	-4.7540	4.4966	-1.0844
H	-4.7390	5.3138	-1.8062
O	-6.0231	3.8125	-1.1752
H	-6.1208	3.4386	-2.0541
C	-3.6081	3.5045	-1.3837
H	-2.6743	4.0702	-1.4059
O	-3.8064	2.9271	-2.6866
H	-3.0674	2.3510	-2.8956
C	-3.4885	2.3859	-0.3284
H	-4.3264	1.6913	-0.4107
O	-2.2573	1.6761	-0.5505
H	-2.1631	0.9841	0.1075
N	2.0311	2.1216	29.6519
O	1.8264	4.4104	30.8572
O	1.0085	3.5204	32.7202
O	4.0108	3.7540	28.8203
O	5.6235	2.4619	29.6453
C	1.7521	1.1079	28.6589
C	1.6147	-0.2537	29.0257
H	1.6451	-0.5397	30.0772
C	1.4422	-1.2190	28.0542
H	1.3345	-2.2650	28.3414
C	1.0263	2.1678	30.7424
H	1.1415	1.3039	31.3971
H	0.0192	2.1768	30.3258
C	1.2850	3.4473	31.5140
C	3.3880	1.8979	30.2189
H	3.6470	0.8449	30.1096
H	3.3769	2.1620	31.2763
C	4.4418	2.7421	29.5095
N	1.7086	2.8637	26.9751
O	2.5131	5.3299	27.0157
O	3.2135	5.2886	24.9085
O	0.1158	4.3002	28.6108
O	-1.6995	3.9829	27.3643
C	1.6830	1.4632	27.3359
C	1.5222	0.4589	26.3494
H	1.4903	0.7326	25.2946
C	1.4060	-0.8652	26.7210
C	2.7610	3.1957	25.9839
H	2.4882	2.8096	25.0019
H	3.7154	2.7677	26.2909
C	2.8545	4.7086	25.9437
C	0.3740	3.2640	26.4545
H	-0.1289	2.3824	26.0571
H	0.5108	3.9952	25.6578
C	-0.4957	3.8794	27.5460
N	0.9234	-1.5010	24.3236
C	1.1964	-1.8897	25.6038
O	1.2858	-3.0703	25.8571
X	2.0967	4.2912	28.7847
H	0.8510	-0.5408	24.0599

302
extended_gt_tagrot1

```
C  0.8409 -2.4821 23.2447
H  0.6155 -3.4452 23.6298
O  2.1886 -2.4372 22.6640
C  2.4341 -3.2980 21.4797
H  2.2658 -4.3402 21.7564
C  3.8927 -3.1166 21.0370
H  4.0945 -2.0732 20.7923
H  4.1001 -3.7233 20.1543
O  4.7597 -3.5347 22.0986
H  4.6088 -2.9811 22.8680
C  1.4281 -2.8886 20.3945
H  1.6279 -1.8465 20.1277
C -0.0190 -2.9858 20.9044
H -0.2562 -4.0219 21.1541
O -0.9132 -2.5419 19.8651
H -1.8196 -2.6129 20.1708
C -0.1998 -2.0832 22.1377
H -0.0153 -1.0499 21.8345
N -1.5731 -2.1893 22.6542
H -1.8125 -3.0577 23.1114
C -2.5165 -1.2364 22.5310
O -2.3363 -0.1567 21.9651
C -3.8870 -1.5822 23.1470
H -3.7580 -2.0756 24.1118
H -4.4692 -0.6715 23.2965
H -4.4326 -2.2436 22.4721
O  1.5994 -3.7598 19.2121
C  1.9772 -3.0273 17.9930
H  2.9060 -2.4852 18.1755
O  0.9134 -2.0655 17.6310
C  1.1089 -1.2725 16.4079
H  2.0297 -0.6941 16.4984
C -0.0837 -0.3162 16.2622
H -1.0192 -0.8732 16.2044
H  0.0173  0.2828 15.3568
O -0.1217  0.5611 17.3963
H -0.2394  0.0427 18.1954
C  1.2383 -2.2790 15.2608
H  0.3031 -2.8443 15.1984
C  2.3820 -3.2769 15.4977
H  3.3368 -2.7479 15.5073
O  2.3877 -4.2357 14.4231
H  3.1141 -4.8498 14.5460
C  2.1692 -4.0200 16.8308
H  1.2633 -4.6254 16.7471
N  3.3111 -4.9036 17.0949
H  4.2267 -4.4947 16.9669
C  3.2230 -6.1974 17.4423
O  2.1590 -6.7746 17.6846
C  4.5634 -6.9511 17.5074
H  5.1615 -6.5706 18.3365
H  4.3901 -8.0186 17.6555
H  5.1136 -6.8158 16.5744
O  1.4729 -1.5254 14.0189
C  0.3833 -1.6980 13.0558
H -0.5671 -1.5766 13.5794
O  0.4307 -3.0420 12.4529
C -0.6581 -3.3597 11.5117
H -1.6159 -3.2414 12.0228
C -0.4966 -4.8233 11.0989
H  0.4322 -4.9708 10.5465
H -1.3336 -5.1374 10.4752
O -0.4703 -5.6151 12.3321
C -0.5575 -2.3457 10.3676
H  0.4067 -2.4857  9.8717
O -1.6657 -2.5687  9.4232
C -0.6201 -0.9024 10.8961
H -1.5978 -0.7394 11.3561
C  0.4626 -0.6312 11.9558
H  0.3146  0.3569 12.3918
O  1.7699 -0.6772 11.3454
H  2.4425 -0.5420 12.0171
O -0.4223  0.0383  9.7829
C -0.4466 -7.0722 12.1995
H -1.2984 -7.3820 11.5915
C -0.6071 -7.6368 13.6176
H -1.3594 -7.0340 14.1327
C  0.6849 -7.5795 14.4577
H  0.8576 -6.5410 14.7449
C  1.9122 -8.1093 13.7010
H  1.8359 -9.1905 13.5655
C  2.0697 -7.4192 12.3338
H  2.2521 -6.3543 12.4941
C  3.2000 -7.9955 11.4705
H  4.1379 -8.0131 12.0272
H  2.9663 -9.0137 11.1572
O  3.3652 -7.1639 10.3153
H  3.6092 -6.2774 10.5934
O  0.8034 -7.5711 11.5794
O  3.0803 -7.8062 14.4845
H  3.0003 -8.2189 15.3471
O  0.5034 -8.3567 15.6559
H -0.2647 -8.0351 16.1330
O -1.0952 -9.0287 13.5052
C -2.4335 -9.2569 14.0799
H -3.1629 -8.6512 13.5388
O -2.4534 -8.8906 15.5145
C -3.7112 -9.1119 16.2554
H -4.5106 -8.5373 15.7822
C -3.5056 -8.6118 17.6898
H -2.6826 -9.1440 18.1684
H -4.4085 -8.7683 18.2811
O -3.2092 -7.2085 17.6587
H -2.4052 -7.0634 17.1540
C -4.0261 -10.6113 16.1562
H -3.2156 -11.1609 16.6449
C -4.1044 -11.0699 14.6893
H -4.9402 -10.5746 14.1912
O -4.3210 -12.4919 14.6638
H -4.3892 -12.7889 13.7532
C -2.7845 -10.7515 13.9581
H -1.9833 -11.3206 14.4344
N -2.8695 -11.1404 12.5435
H -3.4605 -10.5690 11.9564
C -2.2548 -12.2110 12.0054
O -1.5288 -12.9799 12.6400
C -2.5270 -12.4427 10.5070
H -2.4700 -11.5010  9.9584
H -1.7869 -13.1311 10.0947
H -3.5201 -12.8753 10.3807
O -5.3098 -10.8851 16.8339
C -5.1828 -11.7738 17.9996
H -4.4163 -11.3732 18.6663
O -4.7892 -13.1336 17.5782
C -4.6376 -14.1337 18.6563
H -3.9199 -13.7518 19.3846
C -4.0556 -15.4150 18.0435
H -4.6939 -15.7867 17.2412
H -3.9648 -16.1910 18.8035
O -2.7547 -15.1234 17.5132
H -2.8273 -14.4334 16.8495
C -6.0021 -14.2887 19.3598
H -5.9095 -14.9782 20.1994
O -6.9493 -14.8274 18.4118
H -7.8088 -14.9098 18.8319
C -6.4945 -12.9109 19.8595
H -5.8152 -12.5801 20.6474
O -7.8053 -13.0556 20.4370
H -8.0939 -12.2098 20.7881
C -6.5277 -11.8423 18.7467
H -7.3345 -12.0517 18.0430
O -6.7462 -10.5578 19.3554
H -6.7680 -9.8810 18.6752
C -1.2019 -3.0839  8.1296
H -0.6161 -3.9899  8.2977
O -0.3588 -2.0760  7.4573
C  0.2010 -2.4600  6.1513
H  0.7941 -3.3706  6.2679
C  1.1107 -1.3161  5.6935
H  0.5553 -0.3794  5.6681
H  1.5092 -1.5165  4.6982
O  2.2013 -1.1916  6.6168
H  1.8562 -1.0209  7.4974
C -0.9930 -2.7373  5.2273
H -1.5647 -1.8101  5.1236
C -1.9182 -3.8159  5.8205
H -1.3850 -4.7678  5.8728
O -3.0616 -3.9705  4.9611
H -3.6240 -4.6702  5.3019
C -2.4035 -3.4051  7.2257
H -3.0128 -2.5027  7.1364
N -3.2089 -4.4775  7.8259
H -2.7062 -5.3040  8.1156
C -4.5454 -4.4423  7.9792
O -5.2520 -3.4890  7.6458
C -5.1687 -5.7066  8.6024
H -4.5642 -6.0587  9.4398
H -6.1743 -5.4881  8.9653
H -5.2304 -6.4914  7.8466
O -0.4855 -3.1808  3.9127
C -0.8697 -2.2869  2.8113
H -0.6153 -1.2622  3.0868
O -2.3213 -2.3709  2.5580
C -2.8397 -1.5000  1.4837
H -2.5490 -0.4734  1.7086
C -4.3723 -1.5758  1.4926
H -4.7111 -2.5914  1.2880
H -4.7879 -0.9104  0.7361
O -4.8534 -1.1766  2.7828
H -4.4784 -1.7510  3.4550
C -2.1560 -1.9088  0.1634
H -2.4715 -1.2419 -0.6388
O -2.5616 -3.2575 -0.1620
H -2.1358 -3.5297 -0.9779
C -0.6201 -1.8544  0.3253
H -0.3355 -0.8094  0.4641
O  0.0019 -2.3269 -0.8838
H  0.9565 -2.2711 -0.7935
C -0.1118 -2.6739  1.5305
H -0.2217 -3.7429  1.3393
O  1.2764 -2.3653  1.7419
H  1.6100 -2.8813  2.4794
C -1.5653  0.8984  9.4585
H -2.4270  0.2661  9.2391
C -1.1942  1.6923  8.2036
H -0.7171  1.0093  7.4963
C -0.2149  2.8491  8.4825
H  0.7655  2.4210  8.7004
C -0.6595  3.7305  9.6634
H -1.5503  4.3025  9.3964
C -0.9342  2.8821 10.9179
H -0.0028  2.4038 11.2297
C -1.5066  3.6706 12.1027
H -0.8768  4.5310 12.3314
H -2.5124  4.0294 11.8797
O -1.5472  2.8118 13.2512
H -0.6559  2.5344 13.4732
O -1.9106  1.8241 10.5647
O  0.4160  4.6358  9.9712
H  0.6103  5.1729  9.1996
O -0.1015  3.6635  7.3010
```

H	0.1524	3.1132	6.5569
O	-2.4489	2.2116	7.6223
C	-2.7956	1.6585	6.3043
H	-2.9210	0.5771	6.3896
O	-1.7285	1.9561	5.3209
C	-1.9563	1.5358	3.9257
H	-2.1016	0.4540	3.8982
C	-0.7167	1.9098	3.1029
H	-0.5362	2.9843	3.1473
H	-0.8521	1.6247	2.0600
O	0.4245	1.2177	3.6236
H	0.5629	1.4747	4.5381
C	-3.2340	2.2451	3.4546
H	-3.0570	3.3235	3.4870
C	-4.4242	1.9256	4.3715
H	-4.6594	0.8631	4.3187
O	-5.5638	2.6804	3.9196
H	-6.3234	2.4726	4.4688
C	-4.1068	2.3132	5.8277
H	-3.9786	3.3970	5.8772
N	-5.2188	1.9240	6.7068
H	-5.3040	0.9380	6.9105
C	-6.1519	2.7688	7.1852
O	-6.1481	3.9849	6.9829
C	-7.2797	2.1120	8.0040
H	-6.8746	1.3505	8.6732
H	-7.7944	2.8643	8.6037
H	-7.9983	1.6506	7.3268
O	-3.5407	1.8166	2.0783
C	-3.4637	2.9029	1.0929
H	-2.5184	3.4333	1.2239
O	-4.5764	3.8532	1.2828
C	-4.6318	4.9908	0.3414
H	-3.6819	5.5290	0.3929
C	-5.7419	5.9432	0.8049
H	-6.6997	5.4247	0.8592
H	-5.8390	6.7798	0.1125
O	-5.4051	6.4480	2.1045
H	-5.3120	5.7153	2.7174
C	-4.7863	4.4243	-1.0860
H	-4.7707	5.2387	-1.8109
O	-6.0571	3.7423	-1.1740
H	-6.1565	3.3639	-2.0499
C	-3.6434	3.4290	-1.3845
H	-2.7082	3.9924	-1.4107
O	-3.8445	2.8473	-2.6863
H	-3.1074	2.2686	-2.8932
C	-3.5244	2.3119	-0.3262
H	-4.3630	1.6184	-0.4063
O	-2.2943	1.6012	-0.5479
H	-2.2005	0.9100	0.1125
N	2.0349	1.7968	29.9491
O	3.2075	2.8977	31.9853
O	3.3196	4.8967	31.0245
O	1.1747	0.6253	32.2214
O	-0.9292	1.2948	31.9449
C	1.8795	0.9040	28.8220
C	1.3083	1.3562	27.6069
H	1.0475	2.4078	27.4864
C	1.0816	0.4679	26.5749
H	0.6443	0.8210	25.6410
C	2.8325	3.0073	29.6337
H	2.2552	3.6850	29.0047
H	3.7521	2.7284	29.1196
C	3.1540	3.6699	30.9592
C	0.6932	2.1892	30.4574
H	-0.0308	2.1014	29.6474
H	0.7335	3.2235	30.7989
C	0.2477	1.3030	31.6161
N	2.9192	-0.8484	30.1519
O	3.4052	-1.0438	32.6929
O	2.9682	-3.2203	32.7571
O	4.8500	0.9075	30.8337
O	6.3922	-0.0683	29.5611
C	2.2324	-0.4148	28.9550
C	1.9780	-1.3175	27.8929
H	2.2346	-2.3715	28.0014
C	1.4060	-0.8652	26.7210
C	2.2536	-1.9867	30.8313
H	2.3948	-2.9022	30.2571
H	1.1876	-1.7875	30.9422
C	2.9061	-2.1177	32.1937
C	4.3225	-1.2070	29.8136
H	4.3714	-1.4875	28.7615
H	4.6293	-2.0519	30.4302
C	5.2770	-0.0431	30.0599
N	0.9234	-1.5011	24.3236
C	1.1964	-1.8897	25.6038
O	1.2858	-3.0703	25.8571
X	3.1044	0.8166	31.7817
H	0.8510	-0.5408	24.0599

302
extended_gt_tagrot2

```
C  0.8409 -2.4821 23.2447
H  0.6155 -3.4452 23.6298
O  2.1886 -2.4372 22.6640
C  2.4341 -3.2980 21.4797
H  2.2658 -4.3402 21.7564
C  3.8927 -3.1166 21.0370
H  4.0945 -2.0732 20.7923
H  4.1001 -3.7233 20.1543
O  4.7597 -3.5347 22.0986
H  4.6088 -2.9811 22.8680
C  1.4281 -2.8886 20.3945
H  1.6279 -1.8465 20.1277
C -0.0190 -2.9858 20.9044
H -0.2562 -4.0219 21.1541
O -0.9132 -2.5419 19.8651
H -1.8196 -2.6129 20.1708
C -0.1998 -2.0832 22.1377
H -0.0153 -1.0499 21.8345
N -1.5731 -2.1893 22.6542
H -1.8125 -3.0577 23.1114
C -2.5165 -1.2364 22.5310
O -2.3363 -0.1567 21.9651
C -3.8870 -1.5822 23.1470
H -3.7580 -2.0756 24.1118
H -4.4692 -0.6715 23.2965
H -4.4326 -2.2436 22.4721
O  1.5994 -3.7598 19.2121
C  1.9772 -3.0273 17.9930
H  2.9060 -2.4852 18.1755
O  0.9134 -2.0655 17.6310
C  1.1089 -1.2725 16.4079
H  2.0297 -0.6941 16.4984
C -0.0837 -0.3162 16.2622
H -1.0192 -0.8732 16.2044
H  0.0173  0.2828 15.3568
O -0.1217  0.5611 17.3963
H -0.2394  0.0427 18.1954
C  1.2383 -2.2790 15.2608
H  0.3031 -2.8443 15.1984
C  2.3820 -3.2769 15.4977
H  3.3368 -2.7479 15.5073
O  2.3877 -4.2357 14.4231
H  3.1141 -4.8498 14.5460
C  2.1692 -4.0200 16.8308
H  1.2633 -4.6254 16.7471
N  3.3111 -4.9036 17.0949
H  4.2267 -4.4947 16.9669
C  3.2230 -6.1974 17.4423
O  2.1590 -6.7746 17.6846
C  4.5634 -6.9511 17.5074
H  5.1615 -6.5706 18.3365
H  4.3901 -8.0186 17.6555
H  5.1136 -6.8158 16.5744
O  1.4729 -1.5254 14.0189
C  0.3833 -1.6980 13.0558
H -0.5671 -1.5766 13.5794
O  0.4307 -3.0420 12.4529
C -0.6581 -3.3597 11.5117
H -1.6159 -3.2414 12.0228
C -0.4966 -4.8233 11.0989
H  0.4322 -4.9708 10.5465
H -1.3336 -5.1374 10.4752
O -0.4703 -5.6151 12.3321
C -0.5575 -2.3457 10.3676
H  0.4067 -2.4857  9.8717
O -1.6657 -2.5687  9.4232
C -0.6201 -0.9024 10.8961
H -1.5978 -0.7394 11.3561
C  0.4626 -0.6312 11.9558
H  0.3146  0.3569 12.3918
O  1.7699 -0.6772 11.3454
H  2.4425 -0.5420 12.0171
O -0.4223  0.0383  9.7829
C -0.4466 -7.0722 12.1995
H -1.2984 -7.3820 11.5915
C -0.6071 -7.6368 13.6176
H -1.3594 -7.0340 14.1327
C  0.6849 -7.5795 14.4577
H  0.8576 -6.5410 14.7449
C  1.9122 -8.1093 13.7010
H  1.8359 -9.1905 13.5655
C  2.0697 -7.4192 12.3338
H  2.2521 -6.3543 12.4941
C  3.2000 -7.9955 11.4705
H  4.1379 -8.0131 12.0272
H  2.9663 -9.0137 11.1572
O  3.3652 -7.1639 10.3153
H  3.6092 -6.2774 10.5934
O  0.8034 -7.5711 11.5794
O  3.0803 -7.8062 14.4845
H  3.0003 -8.2189 15.3471
O  0.5034 -8.3567 15.6559
H -0.2647 -8.0351 16.1330
O -1.0952 -9.0287 13.5052
C -2.4335 -9.2569 14.0799
H -3.1629 -8.6512 13.5388
O -2.4534 -8.8906 15.5145
C -3.7112 -9.1119 16.2554
H -4.5106 -8.5373 15.7822
C -3.5056 -8.6118 17.6898
H -2.6826 -9.1440 18.1684
H -4.4085 -8.7683 18.2811
O -3.2092 -7.2085 17.6587
H -2.4052 -7.0634 17.1540
C -4.0261 -10.6113 16.1562
H -3.2156 -11.1609 16.6449
C -4.1044 -11.0699 14.6893
H -4.9402 -10.5746 14.1912
O -4.3210 -12.4919 14.6638
H -4.3892 -12.7889 13.7532
C -2.7845 -10.7515 13.9581
H -1.9833 -11.3206 14.4344
N -2.8695 -11.1404 12.5435
H -3.4605 -10.5690 11.9564
C -2.2548 -12.2110 12.0054
O -1.5288 -12.9799 12.6400
C -2.5270 -12.4427 10.5070
H -2.4700 -11.5010  9.9584
H -1.7869 -13.1311 10.0947
H -3.5201 -12.8753 10.3807
O -5.3098 -10.8851 16.8339
C -5.1828 -11.7738 17.9996
H -4.4163 -11.3732 18.6663
O -4.7892 -13.1336 17.5782
C -4.6376 -14.1337 18.6563
H -3.9199 -13.7518 19.3846
C -4.0556 -15.4150 18.0435
H -4.6939 -15.7867 17.2412
H -3.9648 -16.1910 18.8035
O -2.7547 -15.1234 17.5132
H -2.8273 -14.4334 16.8495
C -6.0021 -14.2887 19.3598
H -5.9095 -14.9782 20.1994
O -6.9493 -14.8274 18.4118
H -7.8088 -14.9098 18.8319
C -6.4945 -12.9109 19.8595
H -5.8152 -12.5801 20.6474
O -7.8053 -13.0556 20.4370
H -8.0939 -12.2098 20.7881
C -6.5277 -11.8423 18.7467
H -7.3345 -12.0517 18.0430
O -6.7462 -10.5578 19.3554
H -6.7680 -9.8810 18.6752
C -1.2019 -3.0839  8.1296
H -0.6161 -3.9899  8.2977
O -0.3588 -2.0760  7.4573
C  0.2010 -2.4600  6.1513
H  0.7941 -3.3706  6.2679
C  1.1107 -1.3161  5.6935
H  0.5553 -0.3794  5.6681
H  1.5092 -1.5165  4.6982
O  2.2013 -1.1916  6.6168
H  1.8562 -1.0209  7.4974
C -0.9930 -2.7373  5.2273
H -1.5647 -1.8101  5.1236
C -1.9182 -3.8159  5.8205
H -1.3850 -4.7678  5.8728
O -3.0616 -3.9705  4.9611
H -3.6240 -4.6702  5.3019
C -2.4035 -3.4051  7.2257
H -3.0128 -2.5027  7.1364
N -3.2089 -4.4775  7.8259
H -2.7062 -5.3040  8.1156
C -4.5454 -4.4423  7.9792
O -5.2520 -3.4890  7.6458
C -5.1687 -5.7066  8.6024
H -4.5642 -6.0587  9.4398
H -6.1743 -5.4881  8.9653
H -5.2304 -6.4914  7.8466
O -0.4855 -3.1808  3.9127
C -0.8697 -2.2869  2.8113
H -0.6153 -1.2622  3.0868
O -2.3213 -2.3709  2.5580
C -2.8397 -1.5000  1.4837
H -2.5490 -0.4734  1.7086
C -4.3723 -1.5758  1.4926
H -4.7111 -2.5914  1.2880
H -4.7879 -0.9104  0.7361
O -4.8534 -1.1766  2.7828
H -4.4784 -1.7510  3.4550
C -2.1560 -1.9088  0.1634
H -2.4715 -1.2419 -0.6388
O -2.5616 -3.2575 -0.1620
H -2.1358 -3.5297 -0.9779
C -0.6201 -1.8544  0.3253
H -0.3355 -0.8094  0.4641
O  0.0019 -2.3269 -0.8838
H  0.9565 -2.2711 -0.7935
C -0.1118 -2.6739  1.5305
H -0.2217 -3.7429  1.3393
O  1.2764 -2.3653  1.7419
H  1.6100 -2.8813  2.4794
C -1.5653  0.8984  9.4585
H -2.4270  0.2661  9.2391
C -1.1942  1.6923  8.2036
H -0.7171  1.0093  7.4963
C -0.2149  2.8491  8.4825
H  0.7655  2.4210  8.7004
C -0.6595  3.7305  9.6634
H -1.5503  4.3025  9.3964
C -0.9342  2.8821 10.9179
H -0.0028  2.4038 11.2297
C -1.5066  3.6706 12.1027
H -0.8768  4.5310 12.3314
H -2.5124  4.0294 11.8797
O -1.5472  2.8118 13.2512
H -0.6559  2.5344 13.4732
O -1.9106  1.8241 10.5647
O  0.4160  4.6358  9.9712
H  0.6103  5.1729  9.1996
O -0.1015  3.6635  7.3010
```

H	0.1524	3.1132	6.5569
O	-2.4489	2.2116	7.6223
C	-2.7956	1.6585	6.3043
H	-2.9210	0.5771	6.3896
O	-1.7285	1.9561	5.3209
C	-1.9563	1.5358	3.9257
H	-2.1016	0.4540	3.8982
C	-0.7167	1.9098	3.1029
H	-0.5362	2.9843	3.1473
H	-0.8521	1.6247	2.0600
O	0.4245	1.2177	3.6236
H	0.5629	1.4747	4.5381
C	-3.2340	2.2451	3.4546
H	-3.0570	3.3235	3.4870
C	-4.4242	1.9256	4.3715
H	-4.6594	0.8631	4.3187
O	-5.5638	2.6804	3.9196
H	-6.3234	2.4726	4.4688
C	-4.1068	2.3132	5.8277
H	-3.9786	3.3970	5.8772
N	-5.2188	1.9240	6.7068
H	-5.3040	0.9380	6.9105
C	-6.1519	2.7688	7.1852
O	-6.1481	3.9849	6.9829
C	-7.2797	2.1120	8.0040
H	-6.8746	1.3505	8.6732
H	-7.7944	2.8643	8.6037
H	-7.9983	1.6506	7.3268
O	-3.5407	1.8166	2.0783
C	-3.4637	2.9029	1.0929
H	-2.5184	3.4333	1.2239
O	-4.5764	3.8532	1.2828
C	-4.6318	4.9908	0.3414
H	-3.6819	5.5290	0.3929
C	-5.7419	5.9432	0.8049
H	-6.6997	5.4247	0.8592
H	-5.8390	6.7798	0.1125
O	-5.4051	6.4480	2.1045
H	-5.3120	5.7153	2.7174
C	-4.7863	4.4243	-1.0860
H	-4.7707	5.2387	-1.8109
O	-6.0571	3.7423	-1.1740
H	-6.1565	3.3639	-2.0499
C	-3.6434	3.4290	-1.3845
H	-2.7082	3.9924	-1.4107
O	-3.8445	2.8473	-2.6863
H	-3.1074	2.2686	-2.8932
C	-3.5244	2.3119	-0.3262
H	-4.3630	1.6184	-0.4063
O	-2.2943	1.6012	-0.5479
H	-2.2005	0.9100	0.1125
N	2.0311	2.1216	29.6519
O	1.8264	4.4104	30.8572
O	1.0085	3.5204	32.7202
O	4.0108	3.7540	28.8203
O	5.6235	2.4619	29.6453
C	1.7521	1.1079	28.6589
C	1.6147	-0.2537	29.0257
H	1.6451	-0.5397	30.0772
C	1.4422	-1.2190	28.0542
H	1.3345	-2.2650	28.3414
C	1.0263	2.1678	30.7424
H	1.1415	1.3039	31.3971
H	0.0192	2.1768	30.3258
C	1.2850	3.4473	31.5140
C	3.3880	1.8979	30.2189
H	3.6470	0.8449	30.1096
H	3.3769	2.1620	31.2763
C	4.4418	2.7421	29.5095
N	1.7086	2.8637	26.9751
O	2.5131	5.3299	27.0157
O	3.2135	5.2886	24.9085
O	0.1158	4.3002	28.6108
O	-1.6995	3.9829	27.3643
C	1.6830	1.4632	27.3359
C	1.5222	0.4589	26.3494
H	1.4903	0.7326	25.2946
C	1.4060	-0.8652	26.7210
C	2.7610	3.1957	25.9839
H	2.4882	2.8096	25.0019
H	3.7154	2.7677	26.2909
C	2.8545	4.7086	25.9437
C	0.3740	3.2640	26.4545
H	-0.1289	2.3824	26.0571
H	0.5108	3.9952	25.6578
C	-0.4957	3.8794	27.5460
N	0.9234	-1.5011	24.3236
C	1.1964	-1.8897	25.6038
O	1.2858	-3.0703	25.8571
X	2.0967	4.2912	28.7847
H	0.8510	-0.5408	24.0599

302

Fold90_gg_tagrot1

```
C  0.8411 -2.4821 23.2445
H  0.6154 -3.4451 23.6299
O  2.1892 -2.4371 22.6639
C  2.4359 -3.2943 21.4778
H  2.2731 -4.3391 21.7525
C  3.8912 -3.1054 21.0292
H  4.0865 -2.0605 20.7839
H  4.0976 -3.7096 20.1440
O  4.7663 -3.5190 22.0849
H  4.6199 -2.9637 22.8541
C  1.4251 -2.8874 20.3962
H  1.6215 -1.8446 20.1310
C -0.0215 -2.9884 20.9057
H -0.2543 -4.0242 21.1592
O -0.9158 -2.5502 19.8668
H -1.8228 -2.6248 20.1718
C -0.2002 -2.0830 22.1375
H -0.0146 -1.0496 21.8324
N -1.5748 -2.1875 22.6508
H -1.8108 -3.0471 23.1272
C -2.5285 -1.2510 22.4865
O -2.3509 -0.1831 21.8966
C -3.9057 -1.6010 23.0824
H -3.7912 -2.0899 24.0506
H -4.4947 -0.6925 23.2187
H -4.4368 -2.2669 22.4011
O  1.5917 -3.7564 19.2132
C  1.9702 -3.0167 18.0005
H  2.9064 -2.4866 18.1843
O  0.9131 -2.0383 17.6478
C  1.0946 -1.2455 16.4193
H  2.0073 -0.6522 16.5056
C -0.1101 -0.3057 16.2671
H -1.0377 -0.8754 16.2085
H -0.0139  0.2922 15.3602
O -0.1618  0.5744 17.3978
H -0.2702  0.0557 18.1985
C  1.2396 -2.2534 15.2756
H  0.3232 -2.8478 15.2203
C  2.4158 -3.2049 15.5315
H  3.3338 -2.6243 15.6162
O  2.5415 -4.1141 14.4285
H  3.2926 -4.6940 14.5739
C  2.1487 -3.9969 16.8263
H  1.2184 -4.5574 16.7054
N  3.2350 -4.9487 17.0957
H  4.0733 -4.5701 17.5105
C  3.2059 -6.2534 16.7534
O  2.2464 -6.7997 16.2078
C  4.4851 -7.0528 17.0697
H  4.9634 -6.6805 17.9766
H  4.2374 -8.1055 17.2135
H  5.1805 -6.9670 16.2337
O  1.4522 -1.5181 14.0219
C  0.3405 -1.6805 13.0778
H -0.5914 -1.4306 13.5863
O  0.2629 -3.0781 12.5971
C -0.7179 -3.4077 11.5478
H -1.7154 -3.2215 11.9499
C -0.6097 -4.9123 11.2477
H -1.3987 -5.1854 10.5459
H -0.7714 -5.4907 12.1582
O  0.6883 -5.2798 10.6442
C -0.4835 -2.4600 10.3616
H  0.4997 -2.6659  9.9318
O -1.5424 -2.6764  9.3617
C -0.5301 -0.9957 10.8280
H -1.5136 -0.8035 11.2651
C  0.5301 -0.7154 11.9011
H  0.4336  0.3112 12.2566
O  1.8487 -0.8866 11.3375
H  2.5101 -0.7204 12.0132
O -0.2995 -0.0791  9.6993
C  1.8374 -5.4336 11.5587
H  1.9763 -4.5050 12.1006
C  3.1132 -5.6583 10.7423
H  3.1140 -4.9524  9.9091
C  3.2170 -7.0857 10.1740
H  2.4719 -7.1968  9.3853
C  2.9921 -8.1625 11.2499
H  3.8296 -8.1766 11.9506
C  1.6749 -7.9317 12.0154
H  0.8365 -8.0545 11.3247
C  1.4819 -8.8728 13.2119
H  1.5664 -9.9137 12.8964
H  2.2333 -8.6823 13.9789
O  0.1738 -8.6620 13.7597
H -0.4867 -8.8595 13.0915
O  1.6577 -6.5401 12.5283
O  2.8997 -9.4366 10.5900
H  3.7113 -9.6026 10.1034
O  4.5234 -7.2652  9.5967
H  4.6855 -6.5741  8.9495
O  4.2673 -5.3796 11.6354
C  5.1215 -4.2441 11.2417
H  4.5417 -3.3214 11.2982
O  5.6223 -4.4366  9.8610
C  6.5565 -3.4313  9.3146
H  6.0674 -2.4551  9.3107
C  6.8977 -3.8384  7.8760
H  7.3582 -4.8270  7.8554
H  7.5914 -3.1244  7.4326
O  5.6923 -3.8607  7.0976
H  5.0880 -4.5093  7.4666
C  7.7765 -3.3944 10.2461
H  8.2647 -4.3722 10.2117
C  7.3540 -3.1134 11.6971
H  6.9118 -2.1177 11.7658
O  8.5293 -3.1585 12.5283
H  8.2884 -2.9496 13.4339
C  6.3436 -4.1749 12.1828
H  6.8355 -5.1493 12.1537
N  5.9504 -3.8879 13.5726
H  5.3413 -3.0952 13.7159
C  6.4963 -4.4969 14.6455
O  7.2030 -5.5045 14.5765
C  6.2541 -3.8184 16.0083
H  5.3321 -3.2415 16.0053
H  6.1953 -4.5709 16.7950
H  7.0846 -3.1473 16.2276
O  8.7054 -2.3412  9.7861
C 10.0282 -2.8456  9.3832
H  9.8918 -3.6695  8.6808
O 10.7837 -3.3363 10.5552
C 12.1405 -3.8596 10.2860
H 12.0621 -4.6559  9.5423
C 12.6905 -4.4727 11.5807
H 12.7073 -3.7338 12.3835
H 13.7048 -4.8398 11.4254
O 11.8492 -5.5688 11.9674
H 10.9538 -5.2518 12.1032
C 12.9853 -2.7190  9.6786
H 13.9737 -3.0939  9.4111
O 13.1315 -1.6785 10.6698
H 13.6468 -0.9567 10.3006
C 12.2802 -2.1492  8.4278
H 12.2799 -2.9273  7.6614
O 13.0354 -1.0318  7.9240
H 12.6235 -0.7062  7.1204
C 10.8240 -1.7180  8.7026
H 10.8047 -0.8205  9.3234
O 10.1868 -1.4406  7.4445
H  9.2789 -1.1690  7.5986
C -1.0351 -3.1678  8.0743
H -0.4547 -4.0756  8.2436
O -0.1773 -2.1491  7.4364
C  0.3929 -2.4957  6.1207
H  0.9912 -3.4044  6.2194
C  1.2969 -1.3356  5.6908
H  0.7349 -0.4024  5.6743
H  1.7065 -1.5172  4.6967
O  2.3785 -1.2186  6.6261
H  2.0255 -1.0556  7.5042
C -0.7896 -2.7602  5.1755
H -1.3632 -1.8343  5.0787
C -1.7155 -3.8543  5.7369
H -1.1778 -4.8044  5.7799
O -2.8482 -4.0003  4.8623
H -3.4143 -4.7035  5.1876
C -2.2199 -3.4688  7.1412
H -2.8240 -2.5636  7.0576
N -3.0402 -4.5476  7.7102
H -2.5486 -5.3840  7.9878
C -4.3777 -4.5002  7.8567
O -5.0727 -3.5341  7.5324
C -5.0175 -5.7653  8.4608
H -4.4292 -6.1232  9.3075
H -6.0284 -5.5448  8.8067
H -5.0672 -6.5457  7.7001
O -0.2656 -3.1818  3.8596
C -0.6559 -2.2900  2.7561
H -0.4138 -1.2625  3.0342
O -2.1047 -2.3878  2.4912
C -2.6230 -1.5262  1.4088
H -2.3459 -0.4963  1.6322
C -4.1559 -1.6176  1.4010
H -4.4822 -2.6375  1.1952
H -4.5700 -0.9581  0.6388
O -4.6539 -1.2201  2.6849
H -4.2833 -1.7910  3.3628
C -1.9260 -1.9312  0.0943
H -2.2417 -1.2681 -0.7114
O -2.3140 -3.2839 -0.2307
H -1.8850 -3.5520 -1.0468
C -0.3919 -1.8582  0.2687
H -0.1204 -0.8106  0.4066
O  0.2447 -2.3281 -0.9345
H  1.1972 -2.2611 -0.8376
C  0.1158 -2.6692  1.4798
H  0.0186 -3.7403  1.2902
O  1.4982 -2.3458  1.6995
H  1.8339 -2.8613  2.4370
C -1.4285  0.8007  9.3656
H -2.2940  0.1784  9.1341
C -1.0467  1.6005  8.1162
H -0.5675  0.9211  7.4066
C -0.0716  2.7586  8.4064
H  0.9064  2.3332  8.6375
C -0.5353  3.6409  9.5795
H -1.4293  4.2036  9.3025
C -0.8155  2.7915 10.8326
H  0.1170  2.3233 11.1554
C -1.4104  3.5799 12.0081
H -0.7952  4.4500 12.2384
H -2.4183  3.9237 11.7733
O -1.4507  2.7266 13.1599
H -0.5574  2.4716 13.4003
O -1.7798  1.7251 10.4711
O  0.5274  4.5566  9.8964
H  0.7157  5.1036  9.1308
O  0.0528  3.5744  7.2266
```

H	0.3140	3.0241	6.4837	O	2.9682	-3.2203	32.7571
O	-2.2993	2.1263	7.5337	O	4.8500	0.9075	30.8337
C	-2.6397	1.5950	6.2064	O	6.3922	-0.0683	29.5611
H	-2.7677	0.5133	6.2727	C	2.2324	-0.4148	28.9550
O	-1.5661	1.9064	5.2350	C	1.9780	-1.3175	27.8929
C	-1.7838	1.4996	3.8336	H	2.2346	-2.3715	28.0014
H	-1.9302	0.4184	3.7968	C	1.4060	-0.8652	26.7210
C	-0.5356	1.8764	3.0262	C	2.2536	-1.9867	30.8313
H	-0.3549	2.9511	3.0794	H	2.3948	-2.9022	30.2571
H	-0.6598	1.5973	1.9801	H	1.1876	-1.7875	30.9422
O	0.5996	1.1816	3.5574	C	2.9061	-2.1177	32.1937
H	0.7312	1.4384	4.4727	C	4.3225	-1.2070	29.8136
C	-3.0575	2.2132	3.3594	H	4.3714	-1.4875	28.7615
H	-2.8789	3.2920	3.3949	H	4.6293	-2.0519	30.4302
C	-4.2546	1.8917	4.2664	C	5.2770	-0.0431	30.0599
H	-4.4939	0.8307	4.2006	N	0.9236	-1.5010	24.3235
O	-5.3869	2.6559	3.8143	C	1.1964	-1.8897	25.6038
H	-6.1518	2.4436	4.3545	O	1.2858	-3.0703	25.8571
C	-3.9457	2.2615	5.7288	X	3.1044	0.8166	31.7817
H	-3.8127	3.3438	5.7910	H	0.8510	-0.5408	24.0599
N	-5.0658	1.8689	6.5964				
H	-5.1501	0.8822	6.7973				
C	-6.0065	2.7109	7.0636				
O	-6.0030	3.9271	6.8636				
C	-7.1429	2.0492	7.8677				
H	-6.7439	1.2906	8.5419				
H	-7.6687	2.8006	8.4603				
H	-7.8513	1.5856	7.1805				
O	-3.3572	1.7896	1.9792				
C	-3.2786	2.8768	0.9950				
H	-2.3380	3.4128	1.1339				
O	-4.4002	3.8197	1.1755				
C	-4.4566	4.9553	0.2317				
H	-3.5118	5.5012	0.2905				
C	-5.5790	5.9008	0.6836				
H	-6.5328	5.3745	0.7307				
H	-5.6754	6.7343	-0.0107				
O	-5.2570	6.4094	1.9858				
H	-5.1656	5.6790	2.6013				
C	-4.5958	4.3863	-1.1966				
H	-4.5801	5.2005	-1.9217				
O	-5.8603	3.6952	-1.2940				
H	-5.9505	3.3173	-2.1716				
C	-3.4428	3.3995	-1.4846				
H	-2.5121	3.9697	-1.5030				
O	-3.6286	2.8156	-2.7871				
H	-2.8869	2.2418	-2.9877				
C	-3.3249	2.2855	-0.4242				
H	-4.1582	1.5864	-0.5107				
O	-2.0882	1.5808	-0.6347				
H	-1.9957	0.8907	0.0255				
N	2.0349	1.7968	29.9491				
O	3.2075	2.8977	31.9853				
O	3.3196	4.8967	31.0245				
O	1.1747	0.6253	32.2214				
O	-0.9292	1.2948	31.9449				
C	1.8795	0.9040	28.8220				
C	1.3083	1.3562	27.6069				
H	1.0475	2.4078	27.4864				
C	1.0816	0.4679	26.5749				
H	0.6443	0.8210	25.6410				
C	2.8325	3.0073	29.6337				
H	2.2552	3.6850	29.0047				
H	3.7521	2.7284	29.1196				
C	3.1540	3.6699	30.9592				
C	0.6932	2.1892	30.4574				
H	-0.0308	2.1014	29.6474				
H	0.7335	3.2235	30.7989				
C	0.2477	1.3030	31.6161				
N	2.9192	-0.8484	30.1519				
O	3.4052	-1.0438	32.6929				

302

Fold90_gg_tagrot2

```
C  0.8411 -2.4821 23.2445
H  0.6154 -3.4451 23.6299
O  2.1892 -2.4371 22.6639
C  2.4359 -3.2943 21.4778
H  2.2731 -4.3391 21.7525
C  3.8912 -3.1054 21.0292
H  4.0865 -2.0605 20.7839
H  4.0976 -3.7096 20.1440
O  4.7663 -3.5190 22.0849
H  4.6199 -2.9637 22.8541
C  1.4251 -2.8874 20.3962
H  1.6215 -1.8446 20.1310
C -0.0215 -2.9884 20.9057
H -0.2543 -4.0242 21.1592
O -0.9158 -2.5502 19.8668
H -1.8228 -2.6248 20.1718
C -0.2002 -2.0830 22.1375
H -0.0146 -1.0496 21.8324
N -1.5748 -2.1875 22.6508
H -1.8108 -3.0471 23.1272
C -2.5285 -1.2510 22.4865
O -2.3509 -0.1831 21.8966
C -3.9057 -1.6010 23.0824
H -3.7912 -2.0899 24.0506
H -4.4947 -0.6925 23.2187
H -4.4368 -2.2669 22.4011
O  1.5917 -3.7564 19.2132
C  1.9702 -3.0167 18.0005
H  2.9064 -2.4866 18.1843
O  0.9131 -2.0383 17.6478
C  1.0946 -1.2455 16.4193
H  2.0073 -0.6522 16.5056
C -0.1101 -0.3057 16.2671
H -1.0377 -0.8754 16.2085
H -0.0139  0.2922 15.3602
O -0.1618  0.5744 17.3978
H -0.2702  0.0557 18.1985
C  1.2396 -2.2534 15.2756
H  0.3232 -2.8478 15.2203
C  2.4158 -3.2049 15.5315
H  3.3338 -2.6243 15.6162
O  2.5415 -4.1141 14.4285
H  3.2926 -4.6940 14.5739
C  2.1487 -3.9969 16.8263
H  1.2184 -4.5574 16.7054
N  3.2350 -4.9487 17.0957
H  4.0733 -4.5701 17.5105
C  3.2059 -6.2534 16.7534
O  2.2464 -6.7997 16.2078
C  4.4851 -7.0528 17.0697
H  4.9634 -6.6805 17.9766
H  4.2374 -8.1055 17.2135
H  5.1805 -6.9670 16.2337
O  1.4522 -1.5181 14.0219
C  0.3405 -1.6805 13.0778
H -0.5914 -1.4306 13.5863
O  0.2629 -3.0781 12.5971
C -0.7179 -3.4077 11.5478
H -1.7154 -3.2215 11.9499
C -0.6097 -4.9123 11.2477
H -1.3987 -5.1854 10.5459
H -0.7714 -5.4907 12.1582
O  0.6883 -5.2798 10.6442
C -0.4835 -2.4600 10.3616
H  0.4997 -2.6659  9.9318
O -1.5424 -2.6764  9.3617
C -0.5301 -0.9957 10.8280
H -1.5136 -0.8035 11.2651
C  0.5301 -0.7154 11.9011
H  0.4336  0.3112 12.2566
O  1.8487 -0.8866 11.3375
H  2.5101 -0.7204 12.0132
O -0.2995 -0.0791  9.6993
C  1.8374 -5.4336 11.5587
H  1.9763 -4.5050 12.1006
C  3.1132 -5.6583 10.7423
H  3.1140 -4.9524  9.9091
C  3.2170 -7.0857 10.1740
H  2.4719 -7.1968  9.3853
C  2.9921 -8.1625 11.2499
H  3.8296 -8.1766 11.9506
C  1.6749 -7.9317 12.0154
H  0.8365 -8.0545 11.3247
C  1.4819 -8.8728 13.2119
H  1.5664 -9.9137 12.8964
H  2.2333 -8.6823 13.9789
O  0.1738 -8.6620 13.7597
H -0.4867 -8.8595 13.0915
O  1.6577 -6.5401 12.5283
O  2.8997 -9.4366 10.5900
H  3.7113 -9.6026 10.1034
O  4.5234 -7.2652  9.5967
H  4.6855 -6.5741  8.9495
O  4.2673 -5.3796 11.6354
C  5.1215 -4.2441 11.2417
H  4.5417 -3.3214 11.2982
O  5.6223 -4.4366  9.8610
C  6.5565 -3.4313  9.3146
H  6.0674 -2.4551  9.3107
C  6.8977 -3.8384  7.8760
H  7.3582 -4.8270  7.8554
H  7.5914 -3.1244  7.4326
O  5.6923 -3.8607  7.0976
H  5.0880 -4.5093  7.4666
C  7.7765 -3.3944 10.2461
H  8.2647 -4.3722 10.2117
C  7.3540 -3.1134 11.6971
H  6.9118 -2.1177 11.7658
O  8.5293 -3.1585 12.5283
H  8.2884 -2.9496 13.4339
C  6.3436 -4.1749 12.1828
H  6.8355 -5.1493 12.1537
N  5.9504 -3.8879 13.5726
H  5.3413 -3.0952 13.7159
C  6.4963 -4.4969 14.6455
O  7.2030 -5.5045 14.5765
C  6.2541 -3.8184 16.0083
H  5.3321 -3.2415 16.0053
H  6.1953 -4.5709 16.7950
H  7.0846 -3.1473 16.2276
O  8.7054 -2.3412  9.7861
C 10.0282 -2.8456  9.3832
H  9.8918 -3.6695  8.6808
O 10.7837 -3.3363 10.5552
C 12.1405 -3.8596 10.2860
H 12.0621 -4.6559  9.5423
C 12.6905 -4.4727 11.5807
H 12.7073 -3.7338 12.3835
H 13.7048 -4.8398 11.4254
O 11.8492 -5.5688 11.9674
H 10.9538 -5.2518 12.1032
C 12.9853 -2.7190  9.6786
H 13.9737 -3.0939  9.4111
O 13.1315 -1.6785 10.6698
H 13.6468 -0.9567 10.3006
C 12.2802 -2.1492  8.4278
H 12.2799 -2.9273  7.6614
O 13.0354 -1.0318  7.9240
H 12.6235 -0.7062  7.1204
C 10.8240 -1.7180  8.7026
H 10.8047 -0.8205  9.3234
O 10.1868 -1.4406  7.4445
H  9.2789 -1.1690  7.5986
C -1.0351 -3.1678  8.0743
H -0.4547 -4.0756  8.2436
O -0.1773 -2.1491  7.4364
C  0.3929 -2.4957  6.1207
H  0.9912 -3.4044  6.2194
C  1.2969 -1.3356  5.6908
H  0.7349 -0.4024  5.6743
H  1.7065 -1.5172  4.6967
O  2.3785 -1.2186  6.6261
H  2.0255 -1.0556  7.5042
C -0.7896 -2.7602  5.1755
H -1.3632 -1.8343  5.0787
C -1.7155 -3.8543  5.7369
H -1.1778 -4.8044  5.7799
O -2.8482 -4.0003  4.8623
H -3.4143 -4.7035  5.1876
C -2.2199 -3.4688  7.1412
H -2.8240 -2.5636  7.0576
N -3.0402 -4.5476  7.7102
H -2.5486 -5.3840  7.9878
C -4.3777 -4.5002  7.8567
O -5.0727 -3.5341  7.5324
C -5.0175 -5.7653  8.4608
H -4.4292 -6.1232  9.3075
H -6.0284 -5.5448  8.8067
H -5.0672 -6.5457  7.7001
O -0.2656 -3.1818  3.8596
C -0.6559 -2.2900  2.7561
H -0.4138 -1.2625  3.0342
O -2.1047 -2.3878  2.4912
C -2.6230 -1.5262  1.4088
H -2.3459 -0.4963  1.6322
C -4.1559 -1.6176  1.4010
H -4.4822 -2.6375  1.1952
H -4.5700 -0.9581  0.6388
O -4.6539 -1.2201  2.6849
H -4.2833 -1.7910  3.3628
C -1.9260 -1.9312  0.0943
H -2.2417 -1.2681 -0.7114
O -2.3140 -3.2839 -0.2307
H -1.8850 -3.5520 -1.0468
C -0.3919 -1.8582  0.2687
H -0.1204 -0.8106  0.4066
O  0.2447 -2.3281 -0.9345
H  1.1972 -2.2611 -0.8376
C  0.1158 -2.6692  1.4798
H  0.0186 -3.7403  1.2902
O  1.4982 -2.3458  1.6995
H  1.8339 -2.8613  2.4370
C -1.4285  0.8007  9.3656
H -2.2940  0.1784  9.1341
C -1.0467  1.6005  8.1162
H -0.5675  0.9211  7.4066
C -0.0716  2.7586  8.4064
H  0.9064  2.3332  8.6375
C -0.5353  3.6409  9.5795
H -1.4293  4.2036  9.3025
C -0.8155  2.7915 10.8326
H  0.1170  2.3233 11.1554
C -1.4104  3.5799 12.0081
H -0.7952  4.4500 12.2384
H -2.4183  3.9237 11.7733
O -1.4507  2.7266 13.1599
H -0.5574  2.4716 13.4003
O -1.7798  1.7251 10.4711
O  0.5274  4.5566  9.8964
H  0.7157  5.1036  9.1308
O  0.0528  3.5744  7.2266
```

H	0.3140	3.0241	6.4837
O	-2.2993	2.1263	7.5337
C	-2.6397	1.5950	6.2064
H	-2.7677	0.5133	6.2727
O	-1.5661	1.9064	5.2350
C	-1.7838	1.4996	3.8336
H	-1.9302	0.4184	3.7968
C	-0.5356	1.8764	3.0262
H	-0.3549	2.9511	3.0794
H	-0.6598	1.5973	1.9801
O	0.5996	1.1816	3.5574
H	0.7312	1.4384	4.4727
C	-3.0575	2.2132	3.3594
H	-2.8789	3.2920	3.3949
C	-4.2546	1.8917	4.2664
H	-4.4939	0.8307	4.2006
O	-5.3869	2.6559	3.8143
H	-6.1518	2.4436	4.3545
C	-3.9457	2.2615	5.7288
H	-3.8127	3.3438	5.7910
N	-5.0658	1.8689	6.5964
H	-5.1501	0.8822	6.7973
C	-6.0065	2.7109	7.0636
O	-6.0030	3.9271	6.8636
C	-7.1429	2.0492	7.8677
H	-6.7439	1.2906	8.5419
H	-7.6687	2.8006	8.4603
H	-7.8513	1.5856	7.1805
O	-3.3572	1.7896	1.9792
C	-3.2786	2.8768	0.9950
H	-2.3380	3.4128	1.1339
O	-4.4002	3.8197	1.1755
C	-4.4566	4.9553	0.2317
H	-3.5118	5.5012	0.2905
C	-5.5790	5.9008	0.6836
H	-6.5328	5.3745	0.7307
H	-5.6754	6.7343	-0.0107
O	-5.2570	6.4094	1.9858
H	-5.1656	5.6790	2.6013
C	-4.5958	4.3863	-1.1966
H	-4.5801	5.2005	-1.9217
O	-5.8603	3.6952	-1.2940
H	-5.9505	3.3173	-2.1716
C	-3.4428	3.3995	-1.4846
H	-2.5121	3.9697	-1.5030
O	-3.6286	2.8156	-2.7871
H	-2.8869	2.2418	-2.9877
C	-3.3249	2.2855	-0.4242
H	-4.1582	1.5864	-0.5107
O	-2.0882	1.5808	-0.6347
H	-1.9957	0.8907	0.0255
N	2.0311	2.1216	29.6519
O	1.8264	4.4104	30.8572
O	1.0085	3.5204	32.7202
O	4.0108	3.7540	28.8203
O	5.6235	2.4619	29.6453
C	1.7521	1.1079	28.6589
C	1.6147	-0.2537	29.0257
H	1.6451	-0.5397	30.0772
C	1.4422	-1.2190	28.0542
H	1.3345	-2.2650	28.3414
C	1.0263	2.1678	30.7424
H	1.1415	1.3039	31.3971
H	0.0192	2.1768	30.3258
C	1.2850	3.4473	31.5140
C	3.3880	1.8979	30.2189
H	3.6470	0.8449	30.1096
H	3.3769	2.1620	31.2763
C	4.4418	2.7421	29.5095
N	1.7086	2.8637	26.9751
O	2.5131	5.3299	27.0157
O	3.2135	5.2886	24.9085
O	0.1158	4.3002	28.6108
O	-1.6995	3.9829	27.3643
C	1.6830	1.4632	27.3359
C	1.5222	0.4589	26.3494
H	1.4903	0.7326	25.2946
C	1.4060	-0.8652	26.7210
C	2.7610	3.1957	25.9839
H	2.4882	2.8096	25.0019
H	3.7154	2.7677	26.2909
C	2.8545	4.7086	25.9437
C	0.3740	3.2640	26.4545
H	-0.1289	2.3824	26.0571
H	0.5108	3.9952	25.6578
C	-0.4957	3.8794	27.5460
N	0.9236	-1.5010	24.3235
C	1.1964	-1.8897	25.6038
O	1.2858	-3.0703	25.8571
X	2.0967	4.2912	28.7847
H	0.8510	-0.5408	24.0599

Modelling of bovine galactosyltransferase complexed with compound 12.

In an attempt to understand why the tri- and tetraantennary bisected substrates **14-16** were not converted by galactosyltransferase, a 3D model of the complex with the bisecting GlcNAc of the biantennary glycan **12** bound to the acceptor site of bovine β(1-4) galactosyltransferase was built based on the X-ray structure of the Met344His mutant (PDB code: 1TW5). This structure was selected since this mutation allowed the crystallization of the enzyme with a chitobiose in the acceptor site and UDP-hexanolamine in the donor site. [6]

The bisecting GlcNAc moiety of glycan **12** was overlaid with the GlcNAc acceptor unit of chitobiose in the enzyme binding site by using Biovia Discovery Studio software. The 1,6-arm was well accommodated in the enzyme in an extended *gauche-trans* conformation, as shown in Scheme 5d. For the 1,3-arm, the ψ angle of the $Man^4\alpha1,3$-Man^3 glycosidic linkage (C1-O-C3-C2) was moved from -120º (main value of this torsion for all conformers obtained in the free state) to values around -80º, (overlay of both conformations in Scheme S8) in order to avoid steric clashes with the diphosphate unit located in the donor binding site. According to molecular dynamics simulations and PDB data, the ψ angle of the $Man^4\alpha1,3$-Man^3 linkage is widely distributed from -180º to -60º, [7] thus the conformation used for modelling the complex should be well accessible. It is important to note that both OH-6 of Man4´and OH-4 of Man4, highlighted in red, are pointing towards the enzyme surface in the model (Scheme S7). An additional GlcNAc moiety at these positions should lead to steric clashes with the protein precluding the recognition of the bisecting GlcNAc in the tri- and tetraantennary acceptors **14-16** by the enzyme. This provides an explanation why the bisecting GlcNAc in complex N-glycans with more than two antennae is not galactosylated by the enzyme.

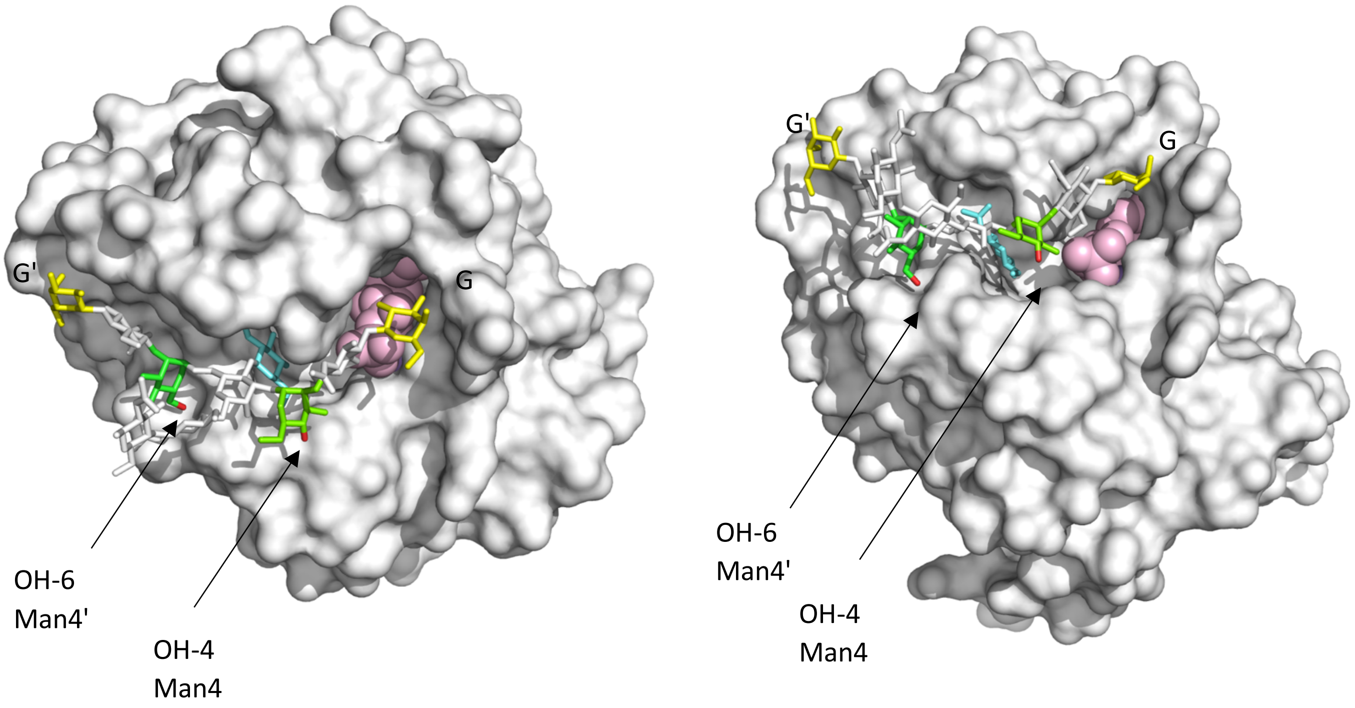

Scheme S7: *Top and side view of a model showing galactosyltransferase in gray (pdb: 1TW5) in complex with the acceptor* ***12****. The inhibitor UDP is shown as spheres in pink. The bisecting GlcNAc of* ***12*** *is colored in cyan, the α-mannoses in green and the terminal galactoses in yellow. OH-6 of Man4' and OH-4 of Man4 are highlighted in red.*

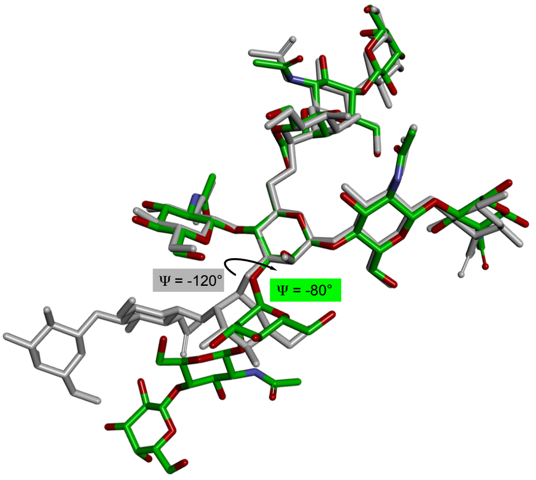

Scheme S8: *Superimposition of the extended gauche-trans conformation of glycan* ***12*** *(in gray) and the adjusted conformation of* ***12*** *(in green) used in the complex with galactosyltransferase. The ψ angles for the Man^{4}α1-3Man^3(C1-O-C3-C2) glycosidic linkage are -120° and -80° respectively.*

[1] H. Kessler, M. Gehrke, C. Griesinger, *Angew. Chem.* **1988**, *100*, 507–554; *Angew. Chem. Int. Ed. Engl.* **1988**, *27*, 490-536.

[2] a) http://www.mestrelab.com Program Mspin. b) Navarro-Vazquez A. *Magn. Reson. Chem*. **2012**, 50 Suppl 1:S73-9.

[3] W. Nishima, N. Miyashita, Y. Yamaguchi, Y. Sugita and S. Re *J.Phys. Res*. B. **2012**, *116*, 8504-8512.

[4] D.A. Case, R.M. Betz, D.S. Cerutti, T.E. Cheatham, III, T.A. Darden, R.E. Duke, T.J. Giese, H. Gohlke, A.W. Goetz, N. Homeyer, S. Izadi, P. Janowski, J. Kaus, A. Kovalenko, T.S. Lee, S. LeGrand, P. Li, C. Lin, T. Luchko, R. Luo, B. Madej, D. Mermelstein, K.M. Merz, G. Monard, H. Nguyen, H.T. Nguyen, I. Omelyan, A. Onufriev, D.R. Roe, A. Roitberg, C. Sagui, C.L. Simmerling, W.M. Botello-Smith, J. Swails, R.C. Walker, J. Wang, R.M. Wolf, X. Wu, L. Xiao and P.A. Kollman (**2016**), AMBER 2016, University of California, San Francisco.

[5] Mizuno M., Funahashi S., Nakasuka N. & Tanaka M. *Inorg. Chem.* **1991**, *30*, 1550-1553.

[6] B. Ramakrishnan, E. Boeggeman, P. K. Qasba, *Biochemistry* **2004**, *43*, 12513-12522.

[7] a) S. Hanashima, A. Suga, Y. Yamaguchi, *Carbohydr. Res*., **2018**, 456 2018, 53e60. b) W. Nishima, N. Miyashita, Y. Yamaguchi, Y. Sugita, S. Re, *J. Phys. Chem. B* **2012**, *116*, 8504−8512).
